# On the Mechanism of Soot Nucleation. IV. Molecular Growth of the Flattened E-Bridge

**DOI:** 10.1021/acs.jpca.2c06819

**Published:** 2022-12-01

**Authors:** Michael Frenklach, Alexander M. Mebel

**Affiliations:** † Department of Mechanical Engineering, University of California, Berkeley, California 94720-1740, United States; ‡ Department of Chemistry and Biochemistry, Florida International University, Miami, Florida 33199, United States

## Abstract

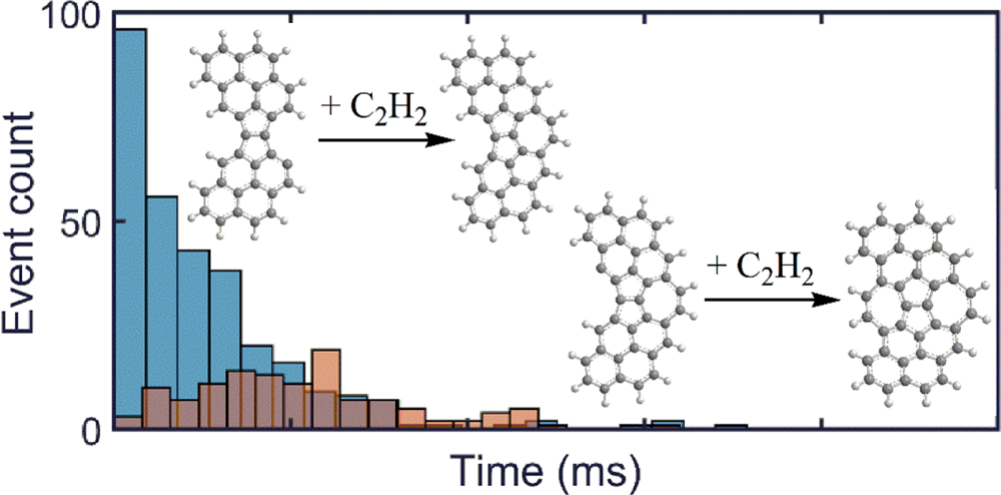

Rotationally excited dimerization of aromatic moieties, a mechanism proposed recently to explain the initial steps of soot particle inception in combustion and pyrolysis of hydrocarbons, produces a molecular structure, termed E-bridge, combining the two aromatics via five-membered aromatic rings sharing a common bond. The present study investigates a hydrogen-mediated addition of acetylene to the fused five-membered ring part of the E-bridge forming a seven-membered ring. The carried out quantum-mechanical and rate theoretical calculations indicate the plausibility of such capping reactions, and kinetic Monte Carlo simulations demonstrate their frequent occurrence. The capping frequency, however, is limited by “splitting” the fused five-membered bridge due to five-membered ring migration. A similar migration of edge seven-membered rings is shown to be also rapid but short, as their encounter with five-membered rings converts them both into six-membered rings.

## 1 Introduction

Recent proposals on combating climate change include utilization of hydrogen.^1^ Technological strategies aimed at production of hydrogen in required amounts include thermal^2^ or plasma-assisted^3^ pyrolysis of natural gas (or, more generally, biomass) that decomposes a hydrocarbon into hydrogen and solid carbon, with the latter then used for production of, say, construction materials. This vision brings to the center the formation of carbonaceous particles in high-temperature environments that has thus far been primarily associated with their negative impact on human health^4^ and the environment^5^ and, hence, has been extensively studied by the combustion community.

Scientifically, the formation of carbonaceous particles in a flame is a fascinating phenomenon: the molecular transition from a few-atom hydrocarbon to a solid material composed of thousands of carbon atoms involves multiple physical and chemical processes, all occurring within a thin layer of a flame on a time scale of a millisecond, with the structure of the forming solid particles exhibiting patterns of self-organization.^6^ Years of research, spanning multiple disciplines and scientific communities, provided a general understanding of the phenomena involved.^7^ Yet, some questions, especially in the area of elementary chemical reactions, still remain. One of them is the specific pathway responsible for particle inception,^7−9^ i.e., the transformation of polycyclic aromatic hydrocarbons (PAH)s, the established precursors,^6,10^ into a “solid” phase.

**Figure 1:**
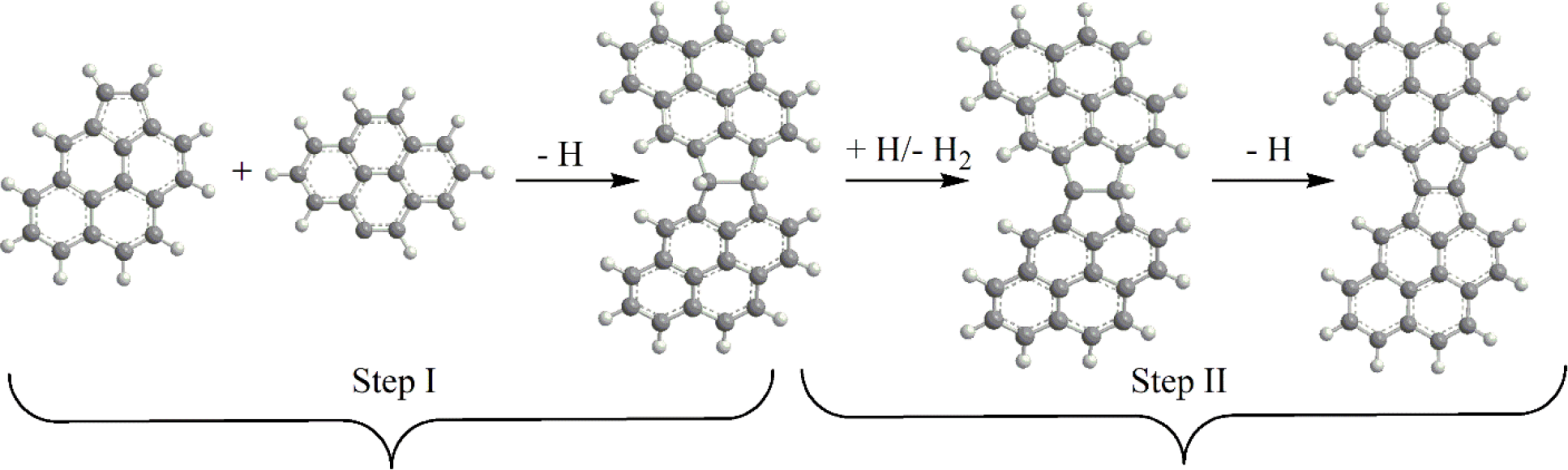
Formation of the flattened E-bridge.

Examination of various proposals made toward the particle inception mechanism^9^ led us to focus on one of the most promising of them, the pathway proceeding through rotationally excited dimerization of the two colliding PAH forming E-bridge, a structure composed of two aromatic rings sharing a common bond (see Figure 1). The initially forming E-bridge, in step I of Figure 1, has the two precursor PAHs at an angle. The immediately following H-abstraction transforms the angled structure into a flattened one,^11^ shown as step II in Figure 1.

The question we pose now is what happens next. Will the flattened E-bridge structure grow just at the outer aromatic structures, i.e., at the E-bridge “wings”? Or could the 5–5 bay of the flattened E-bridge be capped, thus propagating a more balanced growth? (By “capping” we imply the formation of an aromatic ring by addition of a CC moiety, such as acetylene, to a bay zone of a PAH.) With this in mind, in the present study, we investigate one of the immediate possibilities, H-mediated addition of C_2_H_2_ forming a seven-membered ring, and analyze implication of this capping to the follow-up growth of the flattened E-bridge.

## 2 Calculation Methods

### 2.1 Quantum Chemistry

We employed here our standard theoretical approach used earlier in the studies of the E-bridge formation, with the difference in the method for energy refinement by higher-level single-point calculations (see below). In particular, geometries of the reactants, intermediates, transition state (first-order saddle point) structures (referred to as “transition states” in the text), and products of the C_34_H_15_ + C_2_H_2_ and C_36_H_15_ + C_2_H_2_ reactions featuring two consecutive seven-membered ring closures in the bay areas of the flattened E-bridge with pyrene wings (C_34_H_16_, Figure 1) were optimized using the density functional theory (DFT) B3LYP method with the 6-311G(d,p) basis set.^12,13^ Here, the C_34_H_15_ radical is obtained by H abstraction from one of the bay areas of the E-bridge. In turn, the C_36_H_15_ radical, which serves as the reactant in the second reaction, is obtained by H abstraction from the bay area in the C_36_H_16_ product of the first reaction. Vibrational frequencies of all stationary structures were computed at the same B3LYP/6-311G(d,p) level of theory and used to evaluate zero-point vibrational energy (ZPE) corrections and for rate constant calculations. The B3LYP calculations were carried out using the GAUSSIAN 16 software package.^14^ The use of B3LYP geometries and vibrational frequencies in conjunction with higher-level chemically accurate single-point energies has been repeatedly shown to provide kinetically accurate rate constants for PAH growth reactions, see e.g., refs (15) and (16). In a recent paper,^17^ we have also tested the performance of B3LYP for a related ring-closure reaction vs a modern ωB97XD functional which includes dispersion corrections. It appeared that the B3LYP- and ωB97XD-optimized geometries were nearly identical, and the difference in vibrational frequencies of local minima and transition states was under 4% and only ∼1% on average. Moreover, single-point G3(MP2,CC)//B3LYP and G3(MP2,CC)//ωB97XD relative energies agreed with each other within 0.3 kcal/mol. Although the B3LYP and ωB97XD relative energies with the same basis set occasionally disagreed by up to ∼3 kcal/mol, the difference practically disappeared upon the higher-level single-point energy refinement. Here, G3(MP2,CC) calculations are not feasible, and single-point energies of the optimized structures were recalculated using domain based local pair-natural orbital singles and doubles coupled cluster method perturbatively included connected triple excitations (DLPNO-CCSD(T))^18,19^ with Dunning’s cc-pVDZ basis set.^20^ In recent works,^21,22^ we compared the performance of the DLPNO-CCSD(T)/cc-pVDZ method for relative energies in PAH growth (benzannulation) and E-bridge formation reactions with the chemically accurate G3(MP2,CC) approach and found a close agreement, with deviations of less than 1 kcal/mol on average and the maximal deviation of ∼2 kcal/mol. Furthermore, the DLPNO-CCSD(T) results with the cc-pVDZ and cc-pVQZ basis sets were very close to one another with very few exceptions, where the deviation was ∼2 kcal/mol. This comparison indicated that the chemical accuracy of about ∼2 kcal/mol can be normally achieved for molecules of this type by the DLPNO-CCSD(T) method even with the cc-pVDZ basis set, opening the opportunity to carry out accurate calculations for much larger systems, like the systems considered in the present study, than those treatable by the model chemistry G3/G4-type schemes. The DLPNO-CCSD(T)/cc-pVDZ calculations were carried out using the ORCA quantum chemistry code.^23^

### 2.2 Reaction Rate Coefficients

Temperature- and pressure-dependent rate constants for the C_34_H_15_ + C_2_H_2_ and C_36_H_15_ + C_2_H_2_ reactions were evaluated using the Rice-Ramsperger-Kassel-Marcus Master Equation (RRKM-ME) approach.^24^ The MESS software package^25^ was utilized for the RRKM-ME calculations, where partition functions and densities of states for local mimima and numbers of states for transition states were computed within the Rigid-Rotor, Harmonic-Oscillator (RRHO) model. The Lennard-Jones parameters were estimated using the general expressions for ε and σ depending on the molecular mass proposed by Wang and Frenklach^26^ for PAH molecules, whereas the parameters for the bath gas (N_2_) were taken from the papers by Vishnyakov et al.^27,28^ Our previous calculations showed that moderate changes in the Lennard-Jones parameters within the ranges corresponding to four- to six-ring PAHs result in insignificant changes of the calculated pressure-dependent rate constants of less than 10%.^29^ The collisional energy transfer parameters in RRKM-ME calculations were described within the “exponential down” model,^30^ where the temperature dependence of the parameter α for the deactivating wing of the energy transfer function was expressed as α(*T*) = α_300_(*T*/300)^*n*^, where *n* = 0.85 and α_300_ = 247 cm^–1^ are “universal” values proposed by Jasper and Miller for hydrocarbons.^31^

### 2.3 PAH Structure Evolution

PAH structure evolution was examined in kinetic Monte Carlo (kMC) simulations employing a recently developed model^32^ augmented with the E-bridge capping reactions described above and seven-ring migrations discussed in Section 3.3. Briefly, the kMC simulations tracked a single PAH molecular structure evolving in a sooting environment of an atmospheric burner-stabilized flame of ethylene, a stagnation 16.3% C_2_H_4_–23.7% O_2_–Ar flame of Wang and co-workers^33^ (cold gas velocity 8.0 cm/s and burner-to-stagnation surface separation 0.8 cm). The flame was simulated with the FFCM1^34^ gaseous reaction model using Cantera.^35^ The stochastic evolution of PAH structure was simulated using the Gillespie algorithm.^36,37^ The PAH-growth reaction rate constants were calculated using the time-dependent temperature and gaseous species profiles (H, H_2_, C_2_H_2_, CH_3_, O, OH, O_2_) obtained in the flame simulations.

## 3 Results

### 3.1 Potential Energy Surface

**Figure 2:**
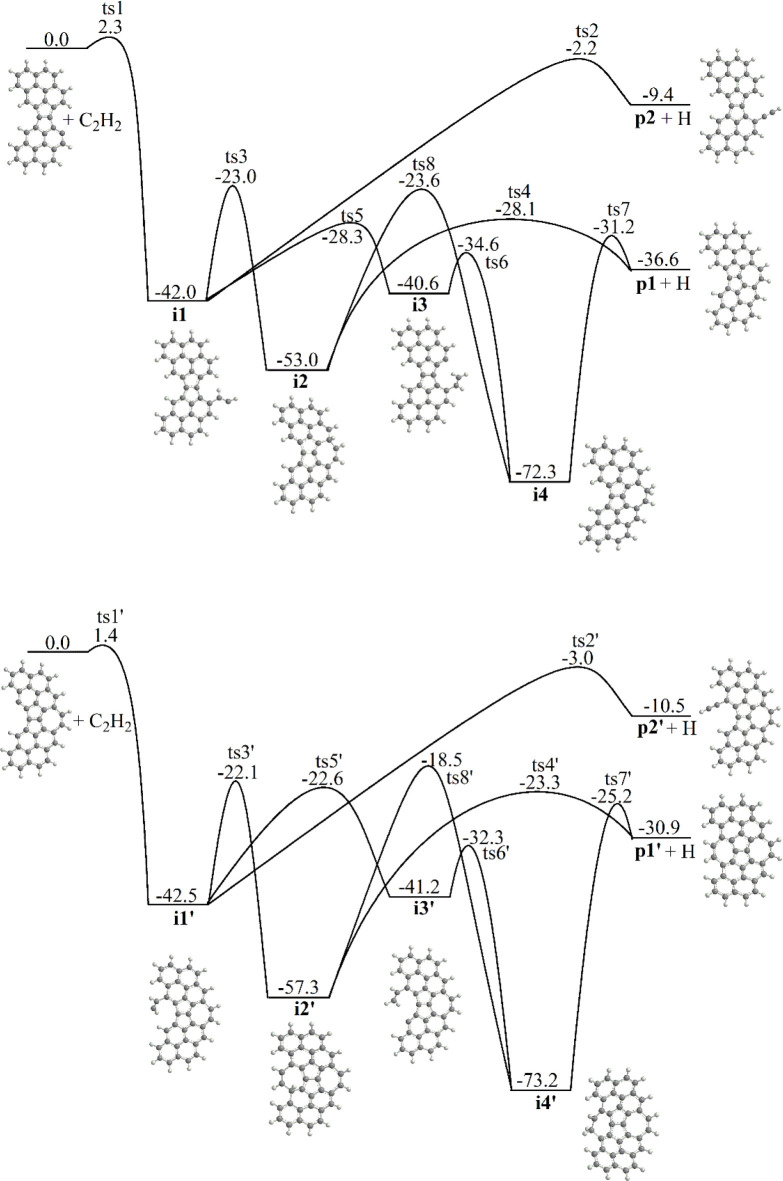
Potential energy diagrams for the C_34_H_15_ + C_2_H_2_ (top) and C_36_H_15_ + C_2_H_2_ (bottom) reactions calculated at the DLPNO-CCSD(T)/cc-pVDZ//B3LYP/6-311G(d,p) + ZPE(B3LYP/6-311G(d,p)) level of theory. Relative energies are shown in kcal/mol with respect to the initial reactants.

Calculated potential energy diagram for the C_34_H_15_ + C_2_H_2_ reaction achieving a seven-membered ring closure in one of the bay areas of the flattened E-bridge with pyrene wings, following its activation through H abstraction is shown in Figure 2. At the initial step, acetylene adds to the radical site producing an initial complex **i1**, C_36_H_17_, overcoming a relatively low barrier of 2.3 kcal/mol at ts1. The complex is stabilized by 42.0 kcal/mol. Next, **i1** can either split an H atom from the C_2_H_2_ moiety producing the ethynyl-substituted E-bridge molecule **p2** via ts2 or undergo a seven-membered ring closure. The H loss occurs via ts2, which resides only 2.2 kcal/mol below the initial reactants, and the product **p2** is computed to be 9.4 kcal/mol exothermic relative to C_34_H_15_ + C_2_H_2_. On the other hand, the ring closure process, which can take place either directly in **i1** or be preceded by 1,7-H migration to form **i3**, is more favorable in terms of enthalpy. The **i1** → **i2** → **p1** + H pathway, where the ring closure is followed by the H loss, leads to the bay-capped product **p1**, C_36_H_16_, exothermic by 36.6 kcal/mol, via transition states ts3 and ts4 positioned 23.0 and 28.1 kcal/mol lower in energy than the reactants. The second three-step pathway to **p1**, **i1** → **i3** → **i4** → **p1** + H, involving the 1,7-H migration, the seven-membered ring closure, and the H elimination, is even more favorable, as the corresponding transition states ts5, ts6, and ts7 are located 28.3, 34.6, and 31.2 kcal/mol below the reactants. The intermediates **i2** and **i4** are connected by a 1,2-H shift via ts8, but this process is less competitive than the H losses from either of the two intermediates. It is apparent from the potential energy diagram that the formation of the thermodynamically favorable bay-capped product **p1** through the well-skipping channels would compete with the production of **p2**, which is preferable by the entropic factor, and, depending on the pressure, with collisional stabilization of the C_36_H_17_ intermediates.

Once the C_36_H_16_**p1** product is formed, it can undergo the HACA sequence (i.e., H abstraction followed by acetylene addition) in the second bay area next to the E-bridge. The corresponding potential energy diagram for the C_36_H_15_ + C_2_H_2_ reaction is illustrated in the bottom half of Figure 2. The reaction mechanism and energetics are rather similar to those for C_34_H_15_ + C_2_H_2_, and the product channels include the well-skipping pathways to the bay-capped product **p1′**, C_36_H_15_ + C_2_H_2_ → **i1′** → **i2′** → **p1′** + H and C_36_H_15_ + C_2_H_2_ → **i1′** → **i3′** → **i4′** → **p1′** + H, competing with the path to the ethynyl-substituted product **p2′**, C_36_H_15_ + C_2_H_2_ → **i1′** → **p2′** + H. The quantitative differences in the relative energies of various intermediates and transition states are mostly minor, within the error margins of the computational method used, but with few notable exceptions. For instance, the energy of the bay-capped product **p1′** with respect to the reactants increases by 5.7 kcal/mol as compared to **p1** making the reaction less exothermic. This can be attributed to the fact that C_36_H_16_**p1** product is still planar, whereas C_38_H_16_**p1′** loses its planarity and attains a bowl-like shape due to the accumulation of the strain energy caused by the presence of two adjacent defects in the graphenic structure consisting of fused seven- and five-membered rings. The energies of the critical transition states on the pathways to **p1′**, ts4′, ts5′, and ts7′, also increase by 5–6 kcal/mol as compared to ts4, ts5, and ts7 in the first bay-capping reaction C_34_H_15_ + C_2_H_2_. This apparently would make the second bay capping of the E-bridge area less favorable than the capping of the first bay area.

### 3.2 Reaction Rate Coefficients

**Figure 3:**
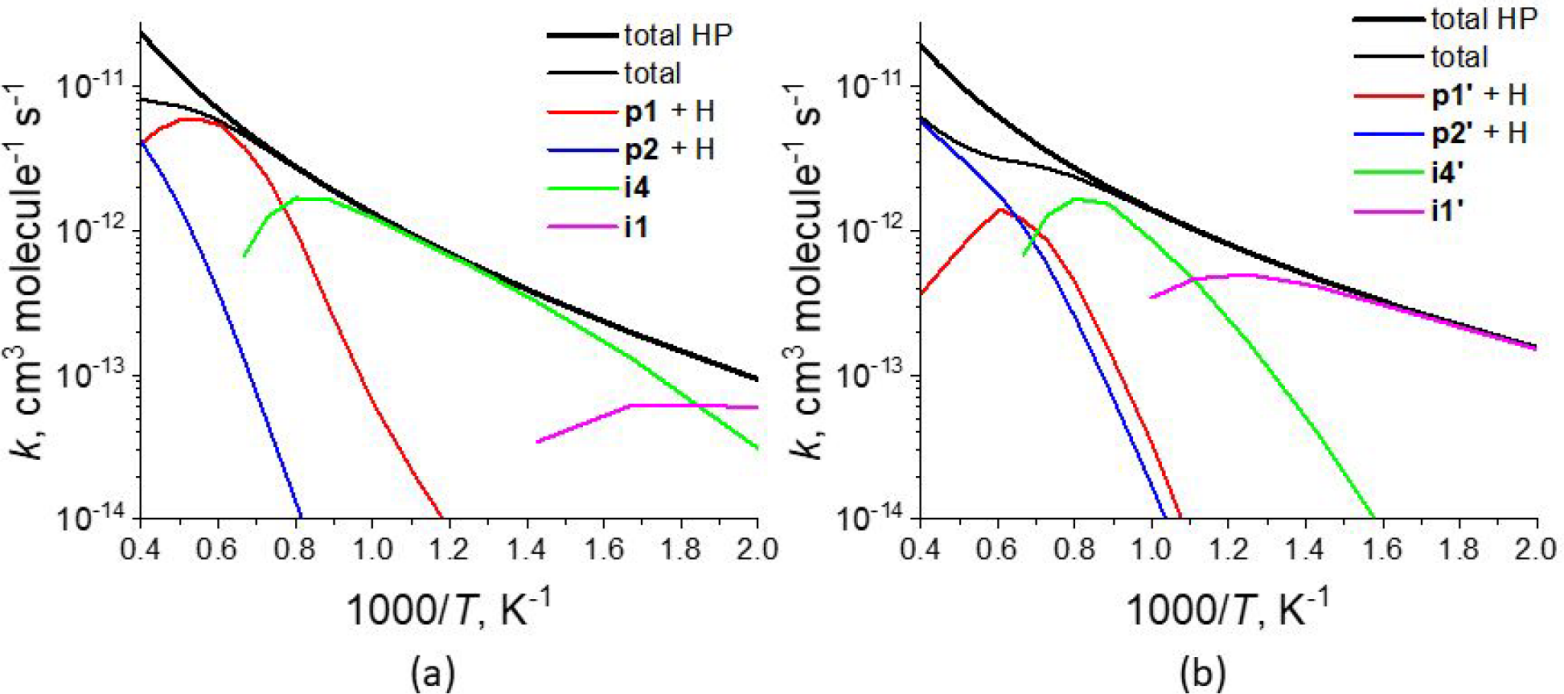
Calculated total and individual channel rate constants for the C_34_H_15_ + C_2_H_2_ (a) and C_34_H_16_ + C_2_H_2_ (b) reactions at 1 atm.

Figure 3 illustrates the calculated rate constants for the C_34_H_15_ + C_2_H_2_ and C_34_H_16_ + C_2_H_2_ reactions at 1 atm. They show a behavior similar to that for other bay-capping reactions occurring via acetylene addition to a σ PAH radical. In particular, the C_34_H_15_ + C_2_H_2_ is fast overall, with the total rate constant at the high-pressure limit (HP) rising from 9.4 × 10^–14^ to 2.3 × 10^–11^ cm^3^ molecule^–1^ s^–1^ in the considered 500–2500 K range. At 1 atm, a falloff of the total rate constant from the HP limit can be seen above 1200 K reaching a factor of ∼2.9 at 2500 K. At lower temperatures, the reaction is dominated by collisional stabilization of the intermediate **i1** and then **i4**, but at *T* > 1250 K, the well-skipping channels leading to the bimolecular products become predominant; **i4** becomes unstable above 1500 K. In the 1300–2500 K temperature range, the bay-capped product **p1** has the highest yield with the rate constant for its formation reaching a maximum at 2000 K. The rate constant for the formation of the ethynyl-substituted product **p2** steadily grows with temperature and nearly equalizes with that for the formation of **p1** at 2500 K. At 1500 K, the bay-capping rate constant involving the reaction of a seven-membered ring closure next to the E-bridge, 3.8 × 10^–12^ cm^3^ molecule^–1^ s^–1^, is similar to those for bay-capping processes with a six-membered ring formation, which were found to vary in the 8.9 × 10^–13^–7.5 × 10^–12^ cm^3^ molecule^–1^ s^–1^ range.^38^

As mentioned above, the second seven-membered closure in the C_36_H_15_ + C_2_H_2_ reaction is less favorable than the closure of the first bay due to the lower exothermicity and higher barriers along the reaction path. This is indeed reflected in the calculated rate constants, displayed in Figure 3. Clearly, the collisional stabilization of **i1′** and **i4′** persists up to higher temperatures, **p1′** is the preferred product only in the narrow temperature range around 1500 K, and the formation of **p2′** prevails at 1650 K and above. The calculated bay-capping rate constant at 1500 K, 1.2 × 10^–12^ cm^3^ molecule^–1^ s^–1^, is about a factor of 3 lower than that for the formation of **p1** in the C_34_H_15_ + C_2_H_2_ reaction.

### 3.3 PAH Structure Evolution

The kMC simulations followed the evolution of the flattened E-bridge “placed” at a flame height corresponding to 1600 K, a likely location of the E-bridge formation. The simulations were carried out for a duration of 3 ms, reaching a temperature of about 1800 K. The statistics of the reaction events were collected from 1,000 kMC runs. For comparison, a similar set of calculations was performed starting with pyrene, as the E-bridge is produced from the derivatives of pyrene, in a reaction between pyrenyl and acepyrene.

**Figure 4:**
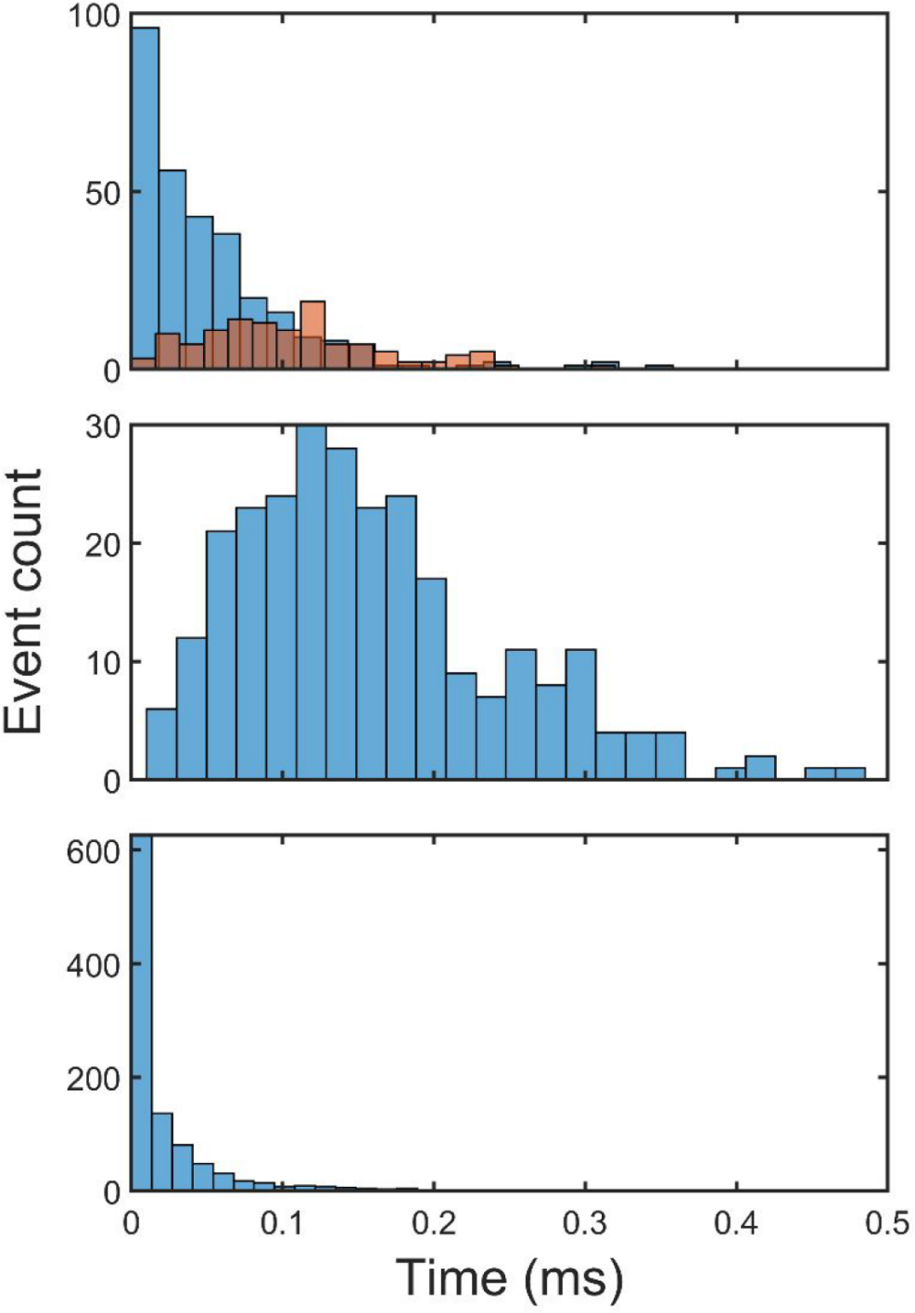
Histograms of reaction events obtained in 1,000 kMC runs. Top panel: first-step capping, reaction C_34_H_15_ + C_2_H_2_ → **p1** + H (blue), and second-step capping, reaction C_36_H_15_ + C_2_H_2_ → **p1′** + H (brown); middle panel: conversion of the seven-membered rings into six-membered rings in R7• + R5 → R6 + R6• (Figure 7, top); bottom panel: split of the “fused” five-membered E-bridge by migration of one of its five-membered rings away from the other.

Out of 1,000 runs, the first E-bridge capping, reaction C_34_H_15_ + C_2_H_2_ → **p1** + H (Figure 2, top), occurred in 302 runs with the second capping event, reaction C_36_H_15_ + C_2_H_2_ → **p1′** + H (Figure 2, bottom), occurring in 122 of the 302 runs; the histogram of these reaction events is displayed in the top panel of Figure 4. These results demonstrate that the capping took place in a considerable number, roughly one-third, of the cases; still, why the capping does not occur in all the cases? The analysis of the reaction pathways revealed that the reason for this is embedded ring migration.

**Figure 5:**
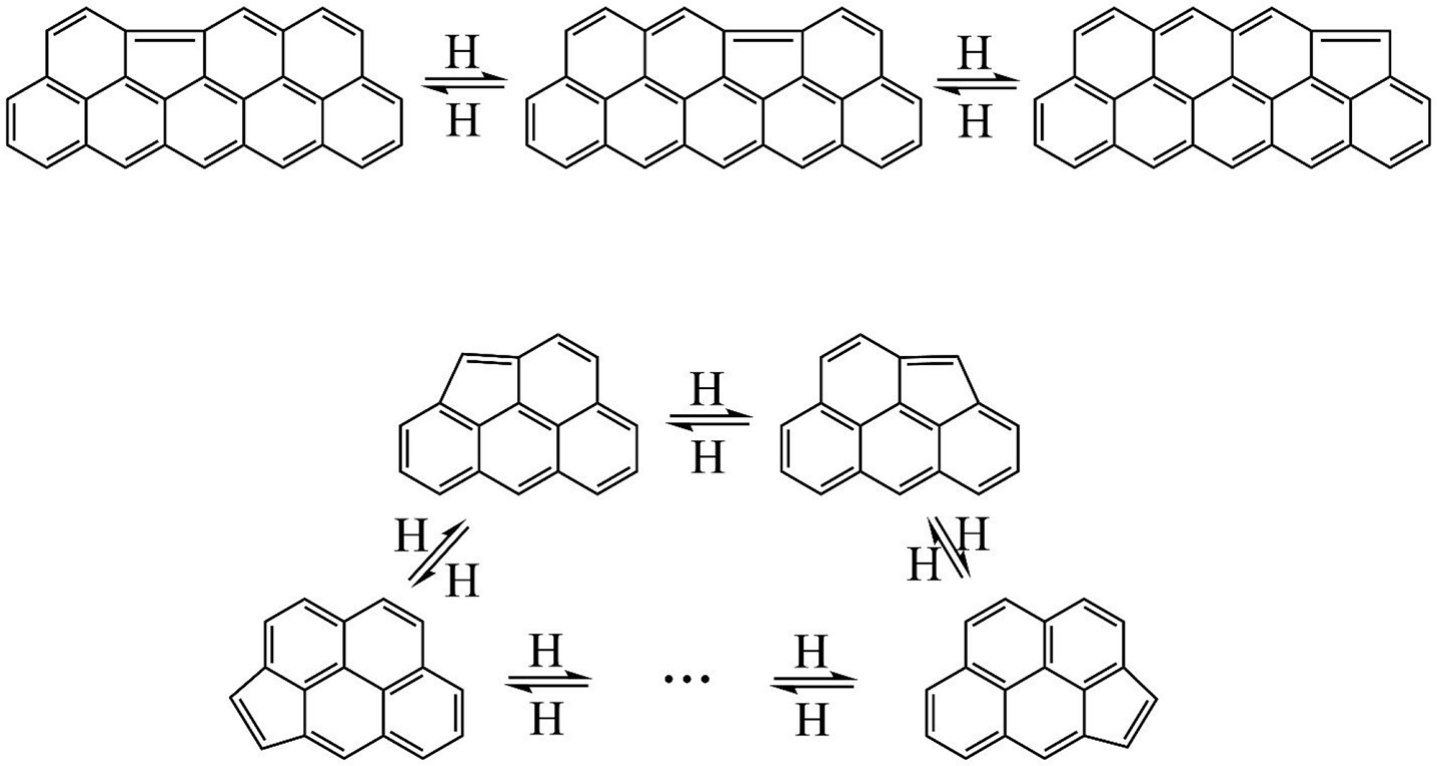
Illustration of the embedded five-membered ring migration. Top panel: migration along a zigzag edge,^39^ bottom panel: perimeter migration.^32^

A theoretical study of Whitesides et al.^39^ suggested that five-membered rings embedded at the graphene edges can undergo rapid H-activated migration along a zigzag edge, as illustrated in the top panel of Figure 5. Tested in kMC simulations,^37,40^ this type of migration was indeed shown to be a frequent event. More recently,^32^ the same mechanism of the five-membered ring migration was shown to be one of the dominant events in the structural evolution of PAHs, exhibiting interconversion among embedded, partially embedded, and zigzag-edge five-membered rings. For a small to a moderate size PAH, such transformations have an appearance of a five-membered ring “rotation” over the perimeter of the evolving structure, as illustrated at the bottom panel of Figure 5.

**Figure 6:**
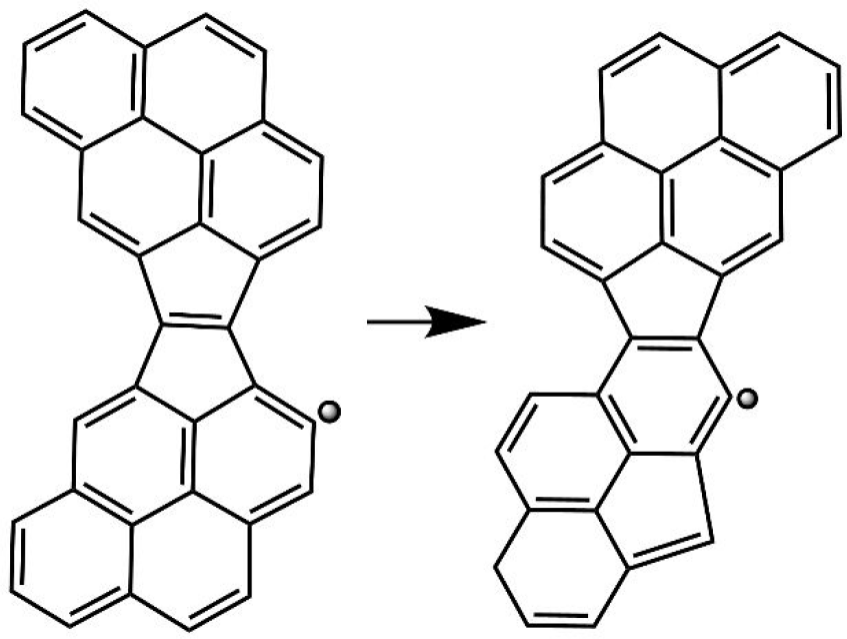
Migration of one of the two fused five-membered rings away from the other.

The present kMC simulations revealed that the embedded five-membered ring migration is causing the split of the two “fused” five-membered rings forming the E-bridge with one of the two five-membered rings moving away from the other, as illustrated in Figure 6. This split happens to occur rather early in the simulation runs, as can be witnessed by the statistics displayed in the bottom panel of Figure 4. The early split of the “fused” five-membered ring structure destroys the E-bridge, which thus prevents the capping reaction from occurring. Still, the results show that roughly in one-third of the cases, the capping, reaction C_34_H_15_ + C_2_H_2_ → **p1** + H, has an opportunity to take place. The second step of capping, reaction C_36_H_15_ + C_2_H_2_ → **p1′** + H, which is slower than the first one to begin with, occurs more rarely as the five-membered ring migration does not cease with the capping.

**Figure 7:**
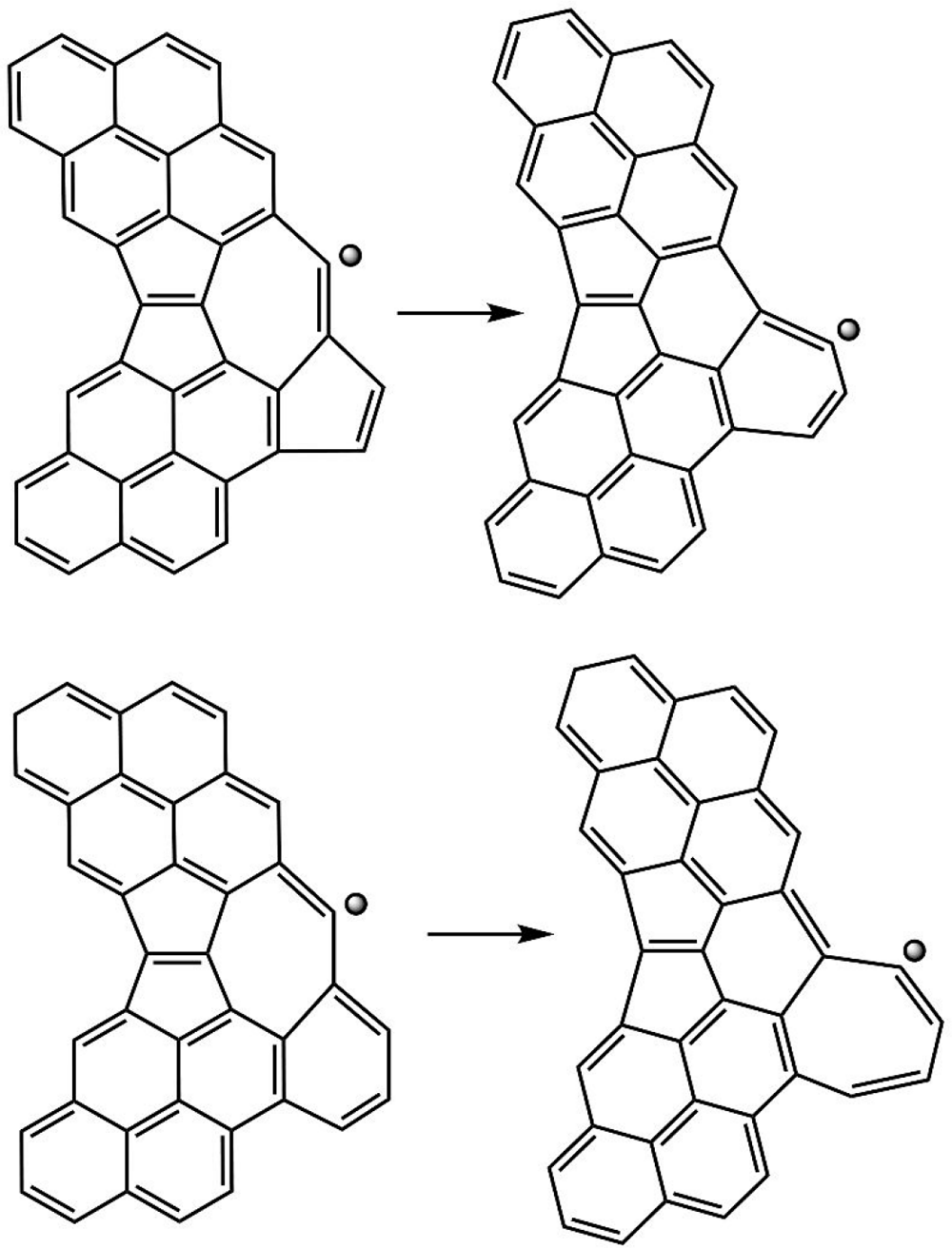
Flip reactions of edge seven-membered rings. Top panel: “collision” of seven- and five-membered rings forming two six-membered rings, R7• + R5 → R6 + R6•, bottom panel: seven-membered ring migration, R7• + R6 → R6 + R7•.

The elementary process underlying the migration of five-membered rings over the aromatic edge is a “flip” reaction,^41^ where the bond common to two adjacent rings switches from one ring to another, induced by the presence of a radical vacancy on the flipped-over ring. A reaction similar to edge five-membered rings can be envisioned for edge seven-membered rings, as illustrated in Figure 7. The top panel of this figure demonstrates a “collision” of edge seven- and five-membered rings forming two edge six-membered rings, and the bottom panel shows a migration step of an edge seven-membered ring.

The flip reactions of edge seven-membered rings were included in the present kMC simulations. Considering the similarity in the underlying mechanism of the flip transformation, we assumed that the seven-membered-ring flip reactions occur with rates similar to those of five-membered rings. The latter were computed^39,41^ to be on the order of 10^9^–10^10^ s^–1^, i.e., occurring much faster than other aromatic edge processes, and therefore could be modeled by partial equilibrium.^37,40^ The same was assumed for the flip reactions of the seven-membered rings in the present kMC simulations.

**Figure 8:**
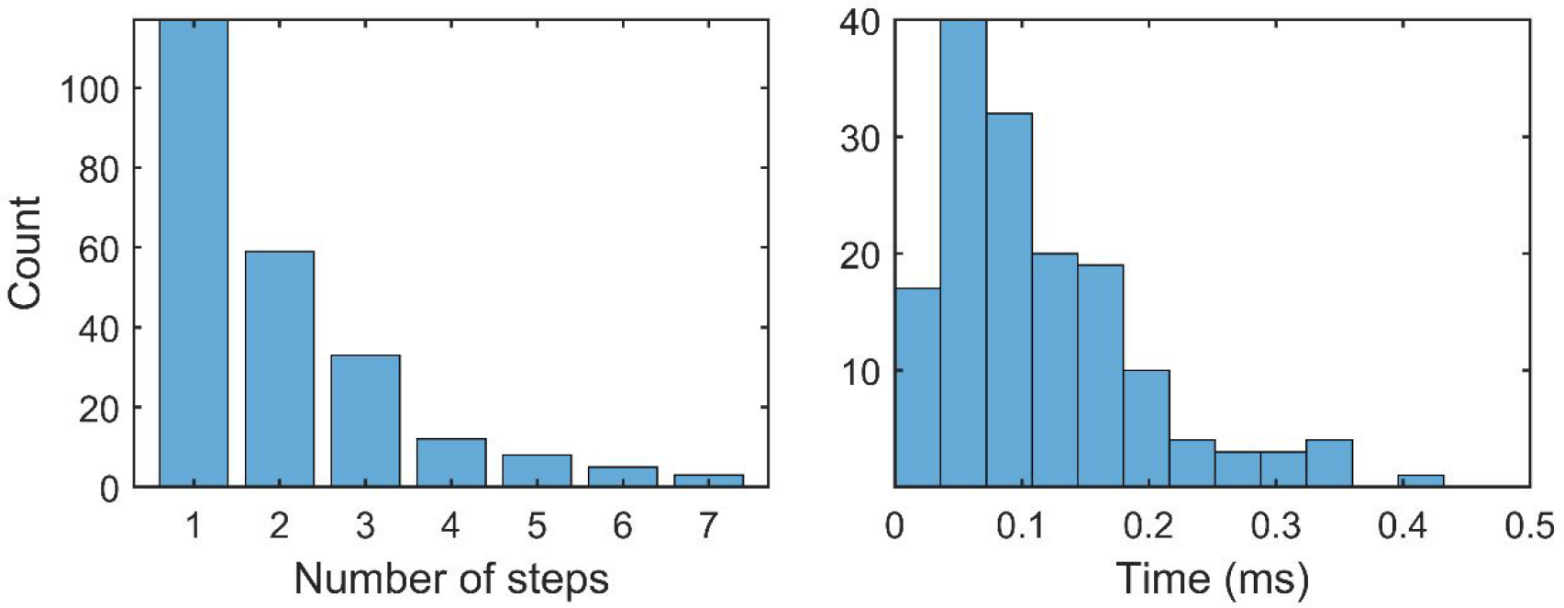
Histograms of seven-membered rings. Left panel: number of migration steps, reaction R7• + R6 → R6 + R7• (Figure 7, bottom); right panel: lifetime of the seven-membered rings.

The computed statistics for the flip reactions of seven-membered ring are shown in Figure 8. As can be seen from the displayed histograms, the edge seven-membered rings clearly undergo migration, yet the length of the migration is rather moderate, just a few steps. The migration of seven-membered rings is rather rapidly terminated by them encountering five-membered rings and thus forming two six-membered rings. This is also collaborated by the relatively short lifetime of seven-membered rings reported in the right panel of Figure 8.

**Figure 9:**
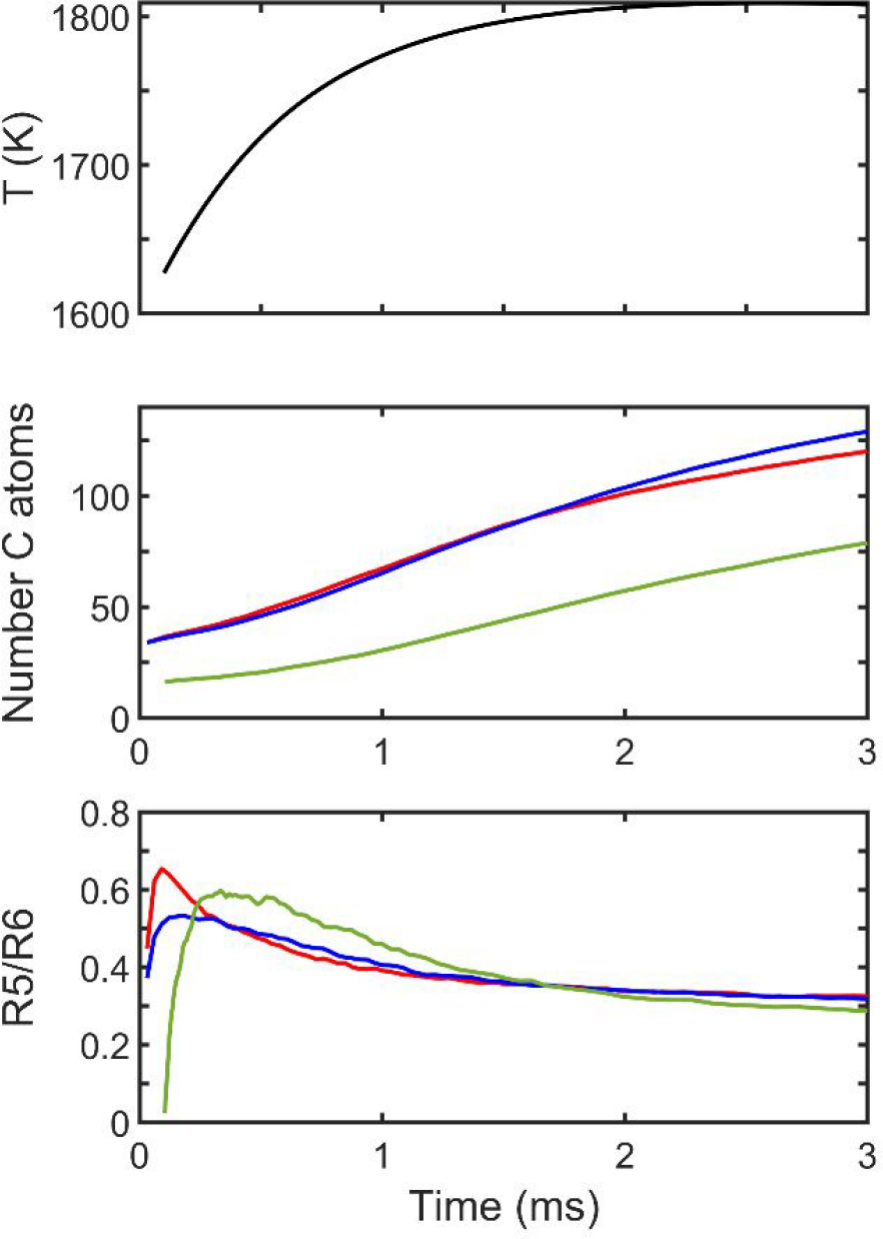
Time evolution of the PAH structure in the modeled flame. Top panel: flame temperature, middle panel: PAH size, and bottom panel: ratio of five- to six-membered rings. Line designation in the middle and bottom panels: with (blue) and without (red) E-bridge capping included starting with the flattened E-bridge, starting with pyrene (green).

The migration of five-membered rings and, as a consequence, the splitting of the fused five-membered rings of the E-bridge, along with the migration of the capping seven-membered rings all lead rapidly to the “memory loss” of the initial E-bridge structure. This explains the computed closeness in the size of PAH structures computed with and without the E-bridge capping included in the kMC simulations and the similarity of their growth rate to that obtained with pyrene as the starting structure, shown in the middle panel of Figure 9. Also, the “collision” of migrating and chemisorbed five- and seven-membered rings forming six-membered rings during their encounters should lead to lower curvature. This, in fact, can be observed in the bottom panel of Figure 9, which displays a lower fraction of five-membered rings during the initial period of simulation, i.e., during the time period when such “collisions” and transformations occur.

## 4 Conclusions

At the conditions simulated in the present study, an H-mediated capping by C_2_H_2_ of the fused five-membered-ring E-bridge occurred relatively frequently but not in all of the runs. It turned out that the embedded five-membered ring migration “split” the fused five-membered rings of the E-bridge rather quickly, before the capping takes place.

Assuming similar elementary flip reactions occurring among edge seven-membered rings and edge five- and six-membered rings revealed rapid but short migration of the edge seven-membered rings at their encounters with six-membered rings and the conversion of seven- and five-membered rings during their mutual encounters into a pair of six-membered rings.

The observed seven-membered ring flip and migration reactions along PAH edges obviously cannot be limited to only seven-membered rings formed in the capping considered in the present study but could happen to any edge seven-membered rings formed in other possible reactions.^42^ While such reactions could be infrequent,^43^ the forming seven-membered rings can be mutually “destroying” themselves and the encountered five-membered rings, by converting both into six-membered rings and thus reducing PAH curvature.

## Supporting Information Available

Table S1 containing calculated rate constants for relevant channels of C_34_H_15_/C_36_H_15_ + C_2_H_2_ reactions at 1 atm and input file for RRKM-ME calculations for C_34_H_15_/C_36_H_15_ + C_2_H_2_ reactions using MESS package, which includes optimized Cartesian coordinates, vibrational frequencies, and relative energies of all structures (PDF)
