# Supplementary material for: On the Mechanism of Soot Nucleation. IV. Molecular Growth of the Flattened E-Bridge
Source: J Phys Chem A. 2022 Dec 1;126(49):9259–67. doi: 10.1021/acs.jpca.2c06819 (PMC9761665; doi:10.1021/acs.jpca.2c06819)
Supplement: Supplementary file 1 — jp2c06819_si_001.pdf [file jp2c06819_si_001.pdf]

Supporting Information for

On the Mechanism of Soot Nucleation. IV. Molecular Growth of the Flatten E-Bridge

Michael Frenklach[*1] *and Alexander M. Mebel*[*2]

[1] *Department of Mechanical Engineering, University of California, Berkeley, California 94720-1740, USA;* orcid.org/0000-0002-9174-3306; Email: frenklach@berkeley.edu

[2] *Department of Chemistry and Biochemistry, Florida International University, Miami, Florida 33199, USA;* orcid.org/0000-0002-7233-3133; Email: mebela@fiu.edu

Table S1. Parameters of fitted modified Arrhenius expressions $k = A\ T^n \exp(-E_a/RT)$ or $k = A_1\ T^{n1} \exp(-E_a^1/RT) + A_2\ T^{n2} \exp(-E_a^2/RT)$ for the considered reactions at 1 atm. Pre-exponential factors A are in cm^3 mol^{-1} s^{-1} and E_a are in cal mol^{-1}.

	A_1	n_1	E_a^1	A_2	n_2	E_a^2
$C_{34}H_{15}$ + C_2H_2 →						
$C_{36}H_{16}$ (**p1**) + H	1.55E+86	-19.798	75473	1.34E+54	-11.794	38277
$C_{36}H_{16}$ (**p2**) + H	2.34E+54	-10.724	65586	8.48E+10	0.95603	24880
$C_{36}H_{16}$ (**p1**) + H →						
$C_{34}H_{15}$ + C_2H_2	1.23E+95	-21.249	114430	2.73E+60	-12.472	75887
$C_{36}H_{16}$ (**p2**) + H	2.19E+95	-21.536	112440	4.65E+57	-11.865	71842
$C_{36}H_{16}$ (**p2**) + H →						
$C_{34}H_{15}$ + C_2H_2	1.26E+61	-12.192	75094	1.05E+18	-0.61124	34626
$C_{36}H_{16}$ (**p1**) + H	2.89E+92	-21.359	82282	9.64E+59	-13.225	44585
$C_{36}H_{15}$ + C_2H_2 →						
$C_{38}H_{16}$ (**p1'**) + H	1.89E+91	-21.678	72822	1.31E+95	-24.997	57079
$C_{38}H_{16}$ (**p2'**) + H	5.73E+46	-8.9079	45818			
$C_{38}H_{16}$ (**p1'**) + H →						
$C_{36}H_{15}$ + C_2H_2	3.00E+99	-22.696	104800			
$C_{38}H_{16}$ (**p2'**) + H	1.04E+99	-22.73	101940			
$C_{38}H_{16}$ (**p2'**) + H →						
$C_{36}H_{15}$ + C_2H_2	1.34E+54	-10.537	56728	1.31E+105	-28.246	62482
$C_{38}H_{16}$ (**p1'**) + H	9.29E+97	-23.282	80704	1.04E+90	-22.888	60825

Input file for RRKM-ME calculations for the $C_{34}H_{15} + C_2H_2$ reaction

```
TemperatureList[K]                    500. 600. 700. 800. 900. 1000. 1125.
1250. 1375. 1500. 1650. 1800. 2000. 2250. 2500.
PressureList[atm]                    0.01 0.03 0.03947368 0.1 0.3 1. 3. 10.
30. 100.
EnergyStepOverTemperature            0.2         #Ratio of discretization
energy step to T
ExcessEnergyOverTemperature          250
ModelEnergyLimit[kcal/mol]           1500
WellCutoff                           10
ChemicalEigenvalueMax                0.2
ChemicalEigenvalueMin                1.e-6         #only for direct
diagonalization method
CalculationMethod                    direct
EigenvalueOutput                                  eigenvalue.out
Reactant        #ground energy of bimolecular species will be used as a
reference.
Model
  EnergyRelaxation
    Exponential
      Factor[1/cm]                   247    ! Jasper universal
      Power                           0.85
      ExponentCutoff                 15
    End
  CollisionFrequency
    LennardJones
         Epsilons[1/cm]          101.5  1283.3  ! N2 chrysene-Frenklach
         Sigmas[angstrom]        3.6154 9.26  ! N2 chrysene-Frenklach
        Masses[amu]            28.  449.13303
    End
OutputTemperatureStep[K]  100
OutputTemperatureSize  20
OutputReferenceEnergy[kcal/mol]  0.
  Well       i1  # i1
  Species
    RRHO
      Geometry[angstrom]    53
 C   -3.4721348893   -1.8278661066   0.0401167414
 C   -4.2400845645   -0.655282349   0.0628692415
 C   -3.549388747   0.589606193   0.0297705115
 C   -2.1673036621   0.57811856   -0.0048425391
 C   -1.3819042713   -0.5914729491   -0.0091212276
 C   -2.0599834815   -1.829791917   -0.0039031364
 C   -4.2186766051   1.8396446145   0.0296161469
 C   -3.452217256   3.0459688491   -0.0080135149
 C   -2.0096031636   2.9870527585   -0.0446631625
 C   -1.368956363   1.7748879946   -0.0408714214
 C   -5.6767493986   -0.5996635351   0.0997545242
 C   -6.3340549694   0.5951216952   0.1032591115
```

```
C   -5.6360353148   1.858509457   0.0679597209
C   -6.2848837393   3.1017361492   0.0679948423
C   -5.5471578246   4.2843635719   0.0309736563
C   -4.1550182823   4.2627996071   -0.0066911491
C   4.1434527706   4.2857369085   -0.1066135093
C   3.4484377337   3.065648801   -0.0742911284
C   4.2229250798   1.8648273526   -0.0175392888
C   5.6401147204   1.8935664491   0.0098441549
C   6.2815983955   3.1411807406   -0.0255064033
C   5.5364282123   4.3170940829   -0.083438843
C   3.5610294845   0.6101370083   0.0174235019
C   4.2608749331   -0.6269496722   0.0911516269
C   5.6953951608   -0.561364248   0.1167260409
C   6.3463127564   0.6372564058   0.0760119855
C   2.0046141406   2.999823678   -0.0909702267
C   1.3705300281   1.785296277   -0.0598695155
C   2.177367235   0.5948475907   -0.0116461476
C   1.3991342453   -0.5776072268   0.0255003153
C   2.0919749052   -1.7934336306   0.1166019338
C   3.4926393697   -1.8066525716   0.1436226752
C   0.0062957362   -0.1098890689   -0.0185518891
C   0.0020262228   1.2931732307   -0.0536479438
C   -1.334220273   -3.1096199321   -0.0794471945
C   -1.7942704579   -4.300651584   0.2346060538
H   -3.9770221585   -2.7879941394   0.0331055968
H   -1.4614873848   3.9232714984   -0.0732830573
H   -6.2374615603   -1.5281900582   0.1254209763
H   -7.4185928849   0.6141454619   0.1321401891
H   -7.3688111606   3.1402960008   0.0965619565
H   -6.0653287563   5.2366449297   0.0313502961
H   -3.6020923103   5.1960142299   -0.035119834
H   3.5845940485   5.2147870735   -0.1496254442
H   7.3654542195   3.1865967187   -0.0063494838
H   6.0480330482   5.2725546775   -0.1097365468
H   6.2627490202   -1.4848073137   0.1704526643
H   7.4308707919   0.6634581748   0.0961268517
H   1.4518868402   3.9331306536   -0.1261610997
H   1.569108008   -2.7389958306   0.1858127897
H   4.0041412281   -2.7609931552   0.2138214177
H   -0.3115407008   -3.0452658332   -0.4588386549
H   -1.434737155   -5.3180682729   0.2388688384
     Core   RigidRotor
       SymmetryFactor          0.5
     End
     Frequencies[1/cm]          153
 29.3185               37.3965                54.3502
 69.6693               94.4200               107.6993
119.1156              150.7090               160.2191
184.7325              194.7870               211.1289
221.4383              238.6536               255.0590
```

```
 275.4214               296.5711               310.7716
 330.9539               353.1250               385.7955
 399.0932               402.3356               413.2824
 423.9045               450.2331               461.5638
 476.7136               495.6808               503.4592
 504.4322               513.4076               525.8261
 532.8792               545.4036               553.9658
 565.0909               574.6328               594.3030
 598.3154               602.6976               610.5021
 612.3213               618.5653               637.4706
 685.5082               688.2779               692.5538
 694.3697               723.2416               747.2536
 754.9872               759.0477               763.6786
 765.7858               775.1311               779.1547
 788.4529               801.5422               813.9988
 818.0810               825.4961               828.4157
 831.0978               850.2449               855.8435
 857.5279               875.5535               889.1020
 899.8192               902.1718               923.0995
 925.4389               928.2354               954.0629
 976.1239               976.1780               981.2561
 986.2049               986.6112               995.3212
1008.3814              1032.0899              1068.5854
1094.4087              1106.0044              1118.5699
1144.1314              1154.5779              1164.9351
1175.6450              1195.0060              1201.1455
1208.3134              1219.5346              1228.2703
1245.6242              1249.6554              1251.1318
1255.4914              1266.3816              1286.4321
1302.9799              1315.7773              1332.8097
1347.8949              1357.8534              1366.7587
1372.6040              1391.9572              1402.7899
1406.3831              1428.4285              1435.5381
1446.4765              1456.1290              1459.9554
1463.5405              1464.2848              1470.8345
1486.5424              1515.9277              1518.4704
1537.5995              1541.9382              1579.5913
1581.5799              1610.5406              1615.8585
1632.9598              1635.2753              1642.3260
1646.5063              1650.5667              1666.3393
1672.4781              3067.0787              3157.5002
3158.0690              3159.8948              3160.1813
3164.1775              3164.5326              3166.3178
3168.0748              3168.4498              3168.8968
3175.9038              3176.5314              3185.2299
3185.4227              3193.9777              3248.1820
      ZeroEnergy[kcal/mol]            -42.0
      ElectronicLevels[1/cm]        1
           0    2
    End
```

```
 End
 Well       i2  # i2
 Species
   RRHO
     Geometry[angstrom]    53
C   -3.3290182751   -1.7941790303   -0.0015919213
C   -4.1479095899   -0.6391305248   -0.059169793
C   -3.5704386406   0.6347546977   0.2320756338
C   -2.2538274317   0.6745418969   0.6422478934
C   -1.5003867815   -0.4832055946   0.7724473918
C   -1.9680020479   -1.7397906303   0.3777828893
C   -4.2382567303   1.8678513685   0.0476429369
C   -3.5115858235   3.0901002743   0.2460716073
C   -2.102967342   3.0746704697   0.5886326224
C   -1.444220698   1.8784794696   0.7821517299
C   -5.5265748712   -0.6139100696   -0.4677461799
C   -6.2080225823   0.5640276679   -0.5968955554
C   -5.5935287975   1.8494619669   -0.3629985742
C   -6.2482596505   3.0797223043   -0.5384752625
C   -5.5629852304   4.2746217661   -0.3291469249
C   -4.2194257867   4.2873751249   0.0469366742
C   4.4809713924   3.6312913901   0.2746204535
C   3.5968925572   2.5545178715   0.4972857778
C   4.1437386622   1.233148274   0.4045319652
C   5.4971609052   1.0062063602   0.0492733022
C   6.325405168   2.1086292692   -0.1579246164
C   5.8151457574   3.408756716   -0.035270802
C   3.2980191827   0.1206432153   0.6344280646
C   3.7092909049   -1.2669963828   0.4131219301
C   5.1063295339   -1.4353899358   0.0400788053
C   5.9358791887   -0.3737328667   -0.1091683716
C   2.1874800736   2.7562599537   0.7441882896
C   1.3551217707   1.6762958886   0.9386364024
C   1.988751495   0.3676751761   0.9484758201
C   1.0356961587   -0.6620490934   1.0528791295
C   1.3740810615   -2.1074055083   1.0311651654
C   2.8071909351   -2.2915312936   0.5273461382
C   -0.1838062845   -0.0368008835   1.0526054415
C   -0.0844184768   1.3887024397   1.0157082397
C   -1.0348445811   -2.8731139708   0.2060583231
C   0.301224588   -3.007703092   0.3941844192
H   -3.7556456877   -2.7444085707   -0.3094688657
H   -1.5895781213   4.028079352   0.6596874783
H   -6.0288819077   -1.5513616215   -0.6838154528
H   -7.2477103347   0.5486608792   -0.9073963873
H   -7.2884786987   3.0972711631   -0.8462179235
H   -6.0811590067   5.2164327111   -0.4712696791
H   -3.7096677858   5.2354170008   0.1835996855
H   4.1046087797   4.6469374534   0.3391062032
H   7.3663668224   1.9594921524   -0.4247905811
```

```
 H   6.4723827157   4.2550831902   -0.200134037
 H   5.4813525355   -2.4415277759   -0.1162569351
 H   6.9736596323   -0.5399692903   -0.3801389429
 H   1.8093788066   3.7733362219   0.7262551536
 H   1.419123197   -2.4455059616   2.0895579399
 H   3.1380142627   -3.3104534646   0.3478326583
 H   -1.5123371705   -3.7548496825   -0.2172203708
 H   0.7033972471   -3.9765774417   0.1054830116
      Core   RigidRotor
        SymmetryFactor          0.5
      End
      Frequencies[1/cm]         153
  23.6104                 46.8070                 78.2273
  91.0370                115.2511                142.8684
 162.6866                172.4203                185.9484
 206.4512                247.8719                261.0755
 264.2090                273.0205                289.4704
 316.0040                324.0525                350.6828
 362.5259                379.6418                402.8451
 422.7163                423.7311                432.5958
 461.6189                482.7446                494.1692
 502.4263                503.5157                514.5190
 515.3594                523.8793                538.1941
 544.6046                549.0960                555.1405
 583.7504                588.0981                592.4013
 600.0983                601.0173                613.6808
 620.7690                686.5490                686.6417
 693.9674                713.4755                729.9100
 734.3391                739.0108                750.8852
 756.9129                765.8035                767.3726
 777.8508                786.5687                793.8951
 805.7528                809.7232                817.1159
 827.4857                829.9925                834.4902
 850.3702                871.5885                880.5420
 885.9093                890.1500                895.3415
 918.0921                919.4805                944.8452
 967.0801                973.0585                975.8090
 982.9864                983.4445                987.4115
 996.2324               1005.4541               1023.7485
1043.9340               1073.1255               1091.8618
1103.1070               1105.5212               1131.6339
1151.5509               1162.5079               1175.8816
1186.2054               1195.2646               1200.9048
1203.8623               1213.6166               1220.7425
1230.0372               1244.4457               1247.3579
1256.3978               1273.7127               1277.4667
1301.9253               1304.7759               1326.8088
1336.6386               1341.5227               1355.0021
1378.4179               1390.1716               1399.8414
1413.9898               1420.3338               1425.3799
```

```
1436.0060              1438.4060              1447.1680
1451.3816              1459.9286              1469.6116
1502.4544              1505.5508              1522.6334
1537.0966              1545.4017              1575.3017
1591.4703              1601.4470              1604.6703
1616.5944              1627.6789              1633.5649
1640.0613              1653.5715              1676.4501
1688.4801              2826.5402              3113.7003
3136.4242              3153.1898              3154.6701
3155.7894              3156.6839              3158.8925
3159.3288              3164.7998              3165.7026
3167.8688              3168.2445              3174.1618
3174.3895              3183.7719              3184.8297
      ZeroEnergy[kcal/mol]            -53.0
      ElectronicLevels[1/cm]        1
           0   2
    End
  End
  Well       i3  # i3
  Species
    RRHO
      Geometry[angstrom]    53
 C   -3.4666995741   -1.9498398898   -0.1537813837
 C   -4.2391347613   -0.7770999708   -0.136363698
 C   -3.5521050475   0.4631647305   -0.018089889
 C   -2.1710268405   0.4481924605   0.0618774889
 C   -1.3864452217   -0.7210096643   0.0429133681
 C   -2.058775342   -1.9619291018   -0.0383904284
 C   -4.2208988969   1.713292699   -0.0071417667
 C   -3.4559009715   2.9177201308   0.0833338196
 C   -2.013905356   2.8572095316   0.1465538373
 C   -1.372713723   1.6457660995   0.128621483
 C   -5.672459011   -0.7196391419   -0.2277306622
 C   -6.3311722655   0.474761398   -0.2042170518
 C   -5.6359039285   1.7348313543   -0.0966757798
 C   -6.2846120671   2.9786002477   -0.0815589524
 C   -5.548762744   4.1585318957   0.0143180853
 C   -4.1579260439   4.1344305061   0.09358456
 C   4.1559290227   4.1402634017   0.1063500097
 C   3.4562763621   2.9224282384   0.0776508154
 C   4.2251425378   1.7196663477   -0.0182227506
 C   5.641408935   1.7467521268   -0.0856268034
 C   6.2869028584   2.9923221698   -0.0525739984
 C   5.5474375828   4.1693451304   0.042564329
 C   3.5592406384   0.4667788681   -0.0509608561
 C   4.2585943216   -0.7710766385   -0.1564571452
 C   5.6909109418   -0.7071718279   -0.2220350399
 C   6.3433587877   0.4912808703   -0.1868273878
 C   2.0138964823   2.863585208   0.1380553156
 C   1.3703546165   1.6532064265   0.1064889784
```

```
C   2.1755867265   0.4631832877   0.0148601334
C   1.3794046983   -0.7070223151   -0.0117557298
C   2.109489151   -1.8747669644   -0.1187401558
C   3.4854456454   -1.9594153538   -0.1906134294
C   -0.0032967212   -0.2359955213   0.0681891833
C   -0.000524707   1.1657990691   0.1301695713
C   -1.3889890097   -3.2720699661   0.0093015016
C   -0.3303708784   -3.5827204065   0.7611462452
H   -3.9767838249   -2.9047705304   -0.236099009
H   -1.466288847   3.7927246298   0.1994895486
H   -6.2311261474   -1.645740554   -0.3149023677
H   -7.4139602065   0.4951761221   -0.2717511439
H   -7.3668934109   3.0189518122   -0.1467829541
H   -6.0664166308   5.1110215881   0.0244553183
H   -3.6059932618   5.0661455232   0.1620136121
H   3.6007944902   5.0697153895   0.1789810381
H   7.3698914498   3.0350769337   -0.1023148337
H   6.0621445058   5.1231323544   0.0668517038
H   6.255367013   -1.6303326143   -0.301304549
H   7.4269904316   0.5164061204   -0.2374980285
H   1.4679053158   3.7991381143   0.205998061
H   3.9828296841   -2.9207241251   -0.2742862005
H   -1.8525377344   -4.0537757038   -0.5887646308
H   0.1348480677   -2.865679487   1.4254582225
H   0.085066908   -4.5834360092   0.7538643963
     Core   RigidRotor
       SymmetryFactor          0.5
     End
     Frequencies[1/cm]         153
 29.7156               40.2286               61.5291
 73.9344               93.1707              121.2752
137.3377              149.4078              157.3303
182.8166              184.9781              207.4267
217.4781              246.8119              261.3771
277.5167              293.3097              300.6180
323.7318              356.6738              382.3273
394.7212              409.3600              413.7719
421.1000              445.5386              464.0395
473.2251              496.2445              501.2699
507.6045              515.3826              525.8682
529.6587              540.6627              550.4117
565.2286              573.5525              592.1136
597.0178              601.8977              606.1847
611.5761              617.0422              647.7350
683.4669              684.9819              694.0654
712.0126              723.2021              740.0871
752.7607              763.3816              767.5792
768.6189              774.7852              784.2730
791.6604              797.6421              804.5508
807.1570              825.1043              831.7985
```

```
 832.9229               859.3444               867.8798
 889.8805               893.3309               901.1871
 914.5276               925.9434               928.8244
 951.9940               973.6821               975.3200
 975.3933               984.5977               985.7294
 986.1460               998.3333              1018.6802
1021.4769              1055.6309              1090.0707
1098.6460              1105.6116              1119.5902
1150.9044              1160.5488              1163.7169
1180.8143              1197.6060              1201.6317
1206.3288              1220.9142              1241.5678
1248.4642              1250.5622              1256.2617
1279.5494              1283.1197              1304.2342
1313.1024              1331.1821              1335.7656
1351.2658              1358.4273              1366.2157
1386.7020              1392.5547              1403.0396
1421.7816              1428.8115              1431.6104
1448.0750              1453.3649              1458.2563
1461.1221              1469.1619              1471.7467
1480.3818              1507.1305              1516.3580
1522.3900              1540.1715              1555.2192
1581.4959              1608.4411              1610.5063
1631.6948              1633.6353              1642.3302
1646.3664              1652.8009              1668.3982
1681.5300              3128.2165              3149.7598
3156.1604              3156.9388              3158.6369
3158.8215              3160.0949              3160.4420
3163.8226              3165.9450              3168.1868
3168.7859              3176.2034              3176.7870
3185.4138              3185.8521              3233.8287
      ZeroEnergy[kcal/mol]            -40.6
      ElectronicLevels[1/cm]        1
          0   2
    End
  End
  Well       i4  # i4
  Species
    RRHO
      Geometry[angstrom]    53
 C   -3.3463769755   -2.1209407409   0.0866905254
 C   -4.1036832894   -0.9377983323   0.0849515488
 C   -3.4453409938   0.3281204381   -0.0946441291
 C   -2.0850158432   0.3269123521   -0.2971282806
 C   -1.3529275819   -0.8522504733   -0.3266400468
 C   -1.9161089941   -2.131136629   -0.1013485238
 C   -4.0945751924   1.5798235643   -0.0194879708
 C   -3.3097857926   2.7742524488   -0.1325603237
 C   -1.8661369974   2.7114700351   -0.2927982261
 C   -1.226247936   1.4993768057   -0.3697541884
 C   -5.5305573751   -0.8598502506   0.2871189855
```

```
C   -6.179680557   0.3402117097   0.3272849852
C   -5.4954093017   1.6079326555   0.1874826834
C   -6.1281594084   2.8564756512   0.2569093371
C   -5.3818416281   4.0293006853   0.1351229975
C   -3.9999625267   3.9950084052   -0.0504400476
C   4.8634956242   2.9894053635   0.0117472456
C   3.9188654322   1.9549820256   -0.0812228319
C   4.4180773184   0.6156833097   0.0241315696
C   5.787982714   0.326211507   0.2291998583
C   6.6845511969   1.4036448745   0.3114422069
C   6.2186703621   2.7120803913   0.2005231016
C   3.5054024953   -0.4603507858   -0.0625205774
C   3.8640764302   -1.8355871655   0.0809470319
C   5.2653389162   -2.0860213921   0.2867282994
C   6.1695922202   -1.0616217139   0.3500744834
C   2.4984485386   2.2169443009   -0.2549247723
C   1.6033681403   1.1785576625   -0.3378716695
C   2.1785822968   -0.1547045417   -0.2554261297
C   1.2088799795   -1.1477477913   -0.3127508222
C   1.4879540752   -2.5010066283   -0.1624071934
C   2.8458558539   -2.8249942814   0.0323767118
C   -0.0138299257   -0.444320523   -0.4300927636
C   0.1453890542   0.9444160759   -0.4441695309
C   -1.1121840328   -3.2910693704   -0.0087282189
C   0.3764291164   -3.5539920219   -0.2803717412
H   -3.8568937957   -3.0646682888   0.253460299
H   -1.3237176973   3.6510827677   -0.3278937893
H   -6.0930403956   -1.7791467428   0.4146360645
H   -7.2536387136   0.3605620719   0.4822808602
H   -7.2008825917   2.9102856944   0.4105626509
H   -5.884873377   4.9880126361   0.1924596259
H   -3.4468027277   4.92524919   -0.1293046858
H   4.5332853916   4.0204640056   -0.0616210213
H   7.7420081907   1.2154267665   0.4649936895
H   6.9222169716   3.5343808965   0.2669624909
H   5.6074967476   -3.1102341195   0.3955532933
H   7.2197785111   -1.2864440767   0.5059191831
H   2.1817165699   3.2541018788   -0.3017005245
H   3.1360428335   -3.8645476537   0.1570366212
H   -1.6468196178   -4.2140637823   0.2027878379
H   0.6556892188   -4.4086818793   0.345592181
H   0.429922069   -3.9506709847   -1.3096483599
     Core   RigidRotor
       SymmetryFactor          0.5
     End
     Frequencies[1/cm]         153
 14.9212               47.6897               77.8785
 89.3112              113.9805              118.4886
147.7255              176.5928              179.5796
206.9639              210.1455              251.5229
```

```
  253.8432              273.4092              284.3257
  303.2822              331.0200              347.0257
  359.2765              369.3222              386.5534
  408.1627              425.2003              426.2213
  464.2402              470.1124              499.1381
  501.2280              504.0159              508.9168
  524.6622              529.8254              536.1606
  542.4090              550.7517              561.1247
  580.3471              592.4418              598.8402
  605.5882              610.1601              615.1223
  617.5855              640.7232              689.6936
  692.4597              695.5425              715.1191
  734.6373              742.8949              746.2471
  754.3020              766.7081              769.2217
  771.4050              780.0923              798.4752
  798.7898              809.9036              812.2386
  828.2914              830.8419              833.0343
  865.5006              869.4160              874.8938
  884.1035              893.2665              899.3610
  918.2675              925.6052              927.4100
  936.2124              970.2675              973.5484
  973.6910              983.2728              984.6485
 1000.5438             1006.2451             1020.6417
 1065.3194             1089.4502             1099.1527
 1106.7306             1127.2252             1137.5606
 1161.0635             1170.5557             1188.4325
 1190.4775             1198.7968             1203.5316
 1219.6506             1229.3263             1239.7537
 1246.6032             1246.8474             1265.7464
 1275.6965             1294.2224             1296.1846
 1306.8125             1322.4178             1340.8270
 1343.3916             1361.4696             1384.1294
 1390.0628             1396.6091             1403.5302
 1424.4313             1433.1824             1439.5141
 1443.5605             1448.6087             1454.5631
 1455.6778             1459.1556             1464.7749
 1474.8985             1510.2386             1514.6482
 1520.4839             1543.5283             1560.6697
 1584.7245             1593.5422             1607.9968
 1619.0192             1626.8368             1634.9910
 1637.4505             1658.7103             1673.7179
 1689.9300             2929.1821             3033.1750
 3134.5082             3149.4578             3154.0676
 3154.4888             3157.3368             3157.9937
 3158.1003             3161.4905             3162.6811
 3166.4600             3166.7433             3172.5386
 3172.8136             3183.1570             3183.4378
       ZeroEnergy[kcal/mol]            -72.3
       ElectronicLevels[1/cm]        1
            0   2
```

```
    End
  End
  Bimolecular      R   # C34H15 + C2H2
    Fragment     C34H15
      RRHO
        Geometry[angstrom]     49
C   -3.4560237967   -3.042073633   0.
C   -4.2418333578   -1.8605723253   0.
C   -3.5561769448   -0.6102734377   0.
C   -2.1714879479   -0.5937262017   0.
C   -1.3639121844   -1.7563444849   0.
C   -2.0801249092   -2.9366434738   0.
C   -4.23670673   0.634997431   0.
C   -3.4798966273   1.8492057252   0.
C   -2.0351002144   1.8105019518   0.
C   -1.3755391119   0.608430106   0.
C   -5.6764718671   -1.8160308371   0.
C   -6.3428041729   -0.6246115219   0.
C   -5.6545677202   0.6424570678   0.
C   -6.3146183531   1.8809296109   0.
C   -5.5872492387   3.0691786527   0.
C   -4.193862324   3.0589150493   0.
C   4.1988489571   3.064946856   0.
C   3.483112515   1.8566677547   0.
C   4.2392896087   0.6424314779   0.
C   5.6566865151   0.6455267034   0.
C   6.3189161957   1.8829182878   0.
C   5.592712138   3.0720087777   0.
C   3.5570796081   -0.6009316072   0.
C   4.2318668333   -1.8547964285   0.
C   5.6676736903   -1.8137503145   0.
C   6.3396172036   -0.6254614594   0.
C   2.0367834541   1.8168076237   0.
C   1.380780805   0.6136590744   0.
C   2.1743494079   -0.5891397995   0.
C   1.3849541672   -1.7515490431   0.
C   2.0434002167   -2.9872309012   0.
C   3.4439564074   -3.0253174566   0.
C   0.0088917607   -1.265932413   0.
C   0.0025779242   0.1357778833   0.
H   -3.9440115453   -4.011547583   0.
H   -1.5008360778   2.7552684959   0.
H   -6.2316115973   -2.7481861353   0.
H   -7.4278710245   -0.6147001065   0.
H   -7.3992025072   1.9094052463   0.
H   -6.112879859   4.0173022869   0.
H   -3.64883964   3.9971676842   0.
H   3.6561041255   4.0045035645   0.
H   7.4035618405   1.9101364918   0.
H   6.1199580207   4.0193028272   0.
```

```
 H   6.2200196322   -2.7478062038   0.
 H   7.424682583   -0.6199252028   0.
 H   1.5016073472   2.7610278436   0.
 H   1.4854982232   -3.9170097176   0.
 H   3.9422905711   -3.9891591865   0.
         Core     RigidRotor
         SymmetryFactor       1
         End
      Frequencies[1/cm]         141
  28.4391               43.2545               75.3787
  93.2767              110.5270              133.1232
 156.9100              182.6531              208.0090
 210.8349              245.2605              255.8006
 260.9987              285.2551              297.1648
 328.8618              380.0700              393.6663
 407.9645              408.8285              425.3395
 426.3592              450.6004              459.5873
 493.2056              494.5905              502.4945
 509.3211              524.6813              526.0000
 528.3369              551.7579              568.0951
 583.5997              590.7293              596.4631
 601.5075              609.3748              611.5977
 617.3441              676.3165              684.3746
 694.0728              698.5645              731.8267
 737.4621              750.9730              755.6807
 757.7671              763.0881              769.4769
 776.5544              789.5776              805.8102
 812.9673              816.7299              825.0129
 826.6024              835.1167              856.5950
 859.5170              875.8544              890.3963
 900.8271              925.9698              925.9859
 928.9210              960.2324              975.5935
 976.2912              977.5196              986.5094
 986.6152              996.7592             1012.2350
1056.3620             1091.7567             1102.5041
1116.4723             1130.1423             1145.3676
1156.0145             1163.8636             1179.6160
1200.0464             1201.8160             1214.3870
1222.7458             1240.0140             1245.9582
1248.6618             1254.5381             1265.8904
1279.3841             1307.2874             1331.1587
1335.4696             1354.5625             1358.6110
1369.1991             1389.0308             1398.6499
1406.6705             1423.6114             1431.6097
1446.2222             1450.6903             1458.0511
1458.3632             1464.1342             1479.9209
1487.4723             1507.2397             1518.2271
1524.3732             1541.3981             1555.2940
1583.3791             1608.3664             1616.8843
1630.9869             1634.7525             1642.8878
```

```
1650.2638              1653.2203              1671.4194
3157.3687              3158.1552              3159.7687
3159.9335              3160.4661              3163.1432
3163.5083              3165.4235              3168.3901
3168.7069              3175.6779              3176.7170
3181.5980              3185.1890              3185.9184
         ZeroEnergy[kcal/mol]         0.
         ElectronicLevels[1/cm]        1
             0   2
    End
Fragment        c2h2
RRHO
Geometry[angstrom]   4
C     0.000000    0.000000    0.599070
C     0.000000    0.000000   -0.599070
H     0.000000    0.000000    1.661908
H     0.000000    0.000000   -1.661908
Core  RigidRotor
SymmetryFactor 2
End
Frequencies[1/cm]  7
642.0679               642.0679               772.6955
772.6955              2069.5209              3420.9273
3523.7963
ZeroEnergy[kcal/mol]     0.0
ElectronicLevels[1/cm]        1
0  1
End
      GroundEnergy[kcal/mol]       0.0
   End
   Bimolecular      p1    # p1 + H
     Fragment        C36H16
       RRHO
       Geometry[angstrom]      52
 C   -3.4145064684   -2.4167518784   -0.2360404359
 C   -4.2223732588   -1.2538121517   -0.2704431622
 C   -3.602785452   0.0384421076   -0.2455768819
 C   -2.2303350979   0.0974027308   -0.1885429061
 C   -1.4695079838   -1.0583616774   -0.1560358171
 C   -1.996115409   -2.3528648364   -0.1770387622
 C   -4.3019032985   1.2632423982   -0.2754594508
 C   -3.5511598943   2.4854079366   -0.2450273724
 C   -2.0979259798   2.4819713041   -0.1845625073
 C   -1.4026821129   1.2964903084   -0.1548753213
 C   -5.6612799384   -1.2364926937   -0.3302948393
 C   -6.3589437711   -0.0611104846   -0.3601044181
 C   -5.716341028   1.2337260572   -0.3342459165
 C   -6.3996086916   2.4575649048   -0.3634640491
 C   -5.6875562132   3.6569621133   -0.3346406843
 C   -4.294059819   3.6777442438   -0.2766974405
```

```
C   4.7372242765   2.8254848889   0.0987239971
C   3.784671165   1.7931441646   0.0601012087
C   4.2938678897   0.4520803493   0.082183941
C   5.6778755676   0.1584793139   0.1395938285
C   6.5777275279   1.2329165345   0.1760068868
C   6.1022809352   2.5443820916   0.1553832495
C   3.378272327   -0.6203461201   0.0450981097
C   3.7455418644   -2.0057284409   0.0611270061
C   5.1623335514   -2.2578970212   0.1198980105
C   6.0672690617   -1.2337482911   0.1566514409
C   2.3564029825   2.0616269023   0.0007346966
C   1.4519620769   1.027099431   -0.0359101684
C   2.0410149785   -0.3056777769   -0.0104497955
C   1.0776950422   -1.2987369614   -0.049799881
C   1.3531952618   -2.668934288   -0.0375833397
C   2.7346694178   -2.9970350863   0.0198365187
C   -0.1419279587   -0.6057016599   -0.1009966639
C   -0.0111106615   0.7825385462   -0.0965505087
C   -1.1067588213   -3.5212363597   -0.1392855274
C   0.2612684171   -3.6503355011   -0.0823169668
H   -3.9032533674   -3.3862981445   -0.2557163718
H   -1.5962491015   3.4442962843   -0.1642897479
H   -6.1982734523   -2.1792723082   -0.3519886589
H   -7.4429350623   -0.0865431551   -0.4051691086
H   -7.4835273638   2.4713729947   -0.4085453917
H   -6.2291090812   4.5960320248   -0.357780407
H   -3.7737823455   4.6297578989   -0.2556815964
H   4.4039529836   3.8580440608   0.0841516275
H   7.6451289767   1.0437118632   0.2202628439
H   6.8097245739   3.3655824425   0.1840256214
H   5.5137484925   -3.284513849   0.1351005346
H   7.1274133273   -1.4615088589   0.2006400275
H   2.0433208709   3.1008320567   -0.0130644529
H   3.0336918957   -4.040921563   0.0328221153
H   -1.6383262301   -4.4699858162   -0.1607867037
H   0.6062263983   -4.6818050293   -0.0673254094
      Core      RigidRotor
        SymmetryFactor       2
      End
      Frequencies[1/cm]       150
 11.6374               49.1959               89.3396
 91.7737              111.1395              144.0668
166.6757              181.5155              191.5934
209.9651              251.1395              255.5281
268.6653              278.3163              299.3796
333.6484              340.7444              352.9457
369.2857              380.8479              411.2825
428.9340              429.1387              449.2900
466.5714              500.2079              501.0175
508.7656              515.8929              522.7408
```

```
 524.5882              531.8820              547.1736
 551.6450              559.4509              563.5608
 594.4588              595.8223              597.8894
 609.5408              613.7612              617.4468
 620.1095              689.7875              694.8241
 696.0687              720.3125              747.6203
 748.0233              756.6632              757.9106
 767.6494              771.3855              776.0548
 776.8146              802.8423              806.3178
 812.1580              817.4905              824.6865
 832.2981              837.5835              838.6539
 877.0522              881.2378              890.3550
 891.2758              894.8622              901.2559
 927.2538              929.0296              974.0917
 974.5982              974.8305              974.9858
 986.1623              986.2812              996.0423
1003.9997             1019.2830             1043.7638
1083.3820             1091.9476             1106.2251
1129.4256             1138.8501             1161.2163
1164.6928             1185.3636             1195.2945
1199.8876             1202.7847             1218.2166
1241.7148             1247.0870             1247.2381
1261.3440             1276.1997             1288.0940
1300.3139             1302.7679             1309.4815
1332.9658             1345.0247             1368.4394
1389.0897             1393.3336             1399.6231
1407.5965             1431.8906             1441.0267
1441.1478             1443.5975             1451.1048
1456.3440             1456.4830             1470.9852
1480.8752             1509.0655             1511.1200
1523.4233             1547.1264             1557.9542
1576.0856             1586.4795             1596.2704
1617.6848             1618.6747             1637.2786
1638.7393             1659.8369             1665.6352
1693.2062             1696.1651             3122.7674
3143.4593             3156.0863             3156.0973
3158.7322             3158.8571             3159.5309
3159.5985             3163.4421             3164.3715
3168.6490             3168.7427             3174.6585
3174.7693             3183.8510             3183.9855
       ZeroEnergy[kcal/mol]          0.
       ElectronicLevels[1/cm]      1
         0    1
       End
     Fragment     H
       Atom
         Mass[amu]   1
         ElectronicLevels[1/cm]      1
            0    2
       End
```

```
    GroundEnergy[kcal/mol]             -36.6
  End
  Bimolecular      p2    # p2 + H
    Fragment        C36H16
      RRHO
      Geometry[angstrom]      52
C   -3.4093789831   -1.9223250782   -0.5179016654
C   -4.1799903662   -0.7495678999   -0.5025142034
C   -3.4909407012   0.4959239737   -0.4891742719
C   -2.1079038711   0.4903334166   -0.4915611265
C   -1.3167226377   -0.6729950711   -0.5065159197
C   -1.9939644303   -1.9138420727   -0.5203384001
C   -4.1640936252   1.744590266   -0.4731731122
C   -3.3998534786   2.9527649526   -0.4603588296
C   -1.9551532227   2.8988727662   -0.4637941895
C   -1.3100833446   1.6898804596   -0.4789536547
C   -5.6166057897   -0.6991179418   -0.49910637
C   -6.2770334799   0.4942996117   -0.4837914504
C   -5.5814549724   1.7588958294   -0.4702430006
C   -6.2343123439   3.0010385247   -0.4542665212
C   -5.499193555   4.1846710769   -0.4417051663
C   -4.1055623091   4.1668284276   -0.444671449
C   4.2310137906   4.1716180431   -0.4545457566
C   3.5249867023   2.9571802862   -0.4679646217
C   4.2902161956   1.7485206178   -0.4806137344
C   5.7080353465   1.7638072747   -0.4795708024
C   6.3599150848   3.0064341043   -0.4659127029
C   5.6243416851   4.1900266954   -0.4536410366
C   3.6182119541   0.4996145327   -0.494309669
C   4.3050864826   -0.7470188119   -0.5066839637
C   5.7403104224   -0.6944090826   -0.5053203952
C   6.4024373338   0.4992486455   -0.492496237
C   2.0805119672   2.9051526455   -0.469031718
C   1.4351183785   1.6954972152   -0.482203312
C   2.2344133666   0.4983915878   -0.4950166666
C   1.4512615427   -0.6690926404   -0.5071663876
C   2.1255619711   -1.8976694919   -0.5193258883
C   3.5260174178   -1.922552683   -0.5190025739
C   0.0659382245   -0.1929838601   -0.5024589058
C   0.0628688504   1.2110318125   -0.4871239304
C   -1.3127410969   -3.1666317515   -0.5366632913
C   -0.7372679056   -4.2249391831   -0.5504866835
H   -3.9090362587   -2.8842046346   -0.5283023999
H   -1.4099247876   3.8371364673   -0.4542973441
H   -6.1752478377   -1.629182912   -0.5088890071
H   -7.3619678389   0.5099019585   -0.481539137
H   -7.3187176783   3.0361699217   -0.4517236077
H   -6.0193683834   5.1358549036   -0.429468692
H   -3.554790867   5.1017167212   -0.4347280062
H   3.6800098008   5.106423104   -0.4447581334
```

```
H   7.4444059783   3.0423199556   -0.4649215969
H   6.1442501506   5.1414155313   -0.4431698658
H   6.3002696499   -1.6240002943   -0.5146620025
H   7.4874691307   0.514360545   -0.4917843234
H   1.5365241488   3.8443468288   -0.4589042323
H   1.5740180383   -2.8287507868   -0.5291217493
H   4.0322537023   -2.8824761503   -0.5285241006
H   -0.2450335514   -5.1663723563   -0.5619481944
      Core      RigidRotor
        SymmetryFactor       1.0
      End
      Frequencies[1/cm]       150
  29.2087              38.7508              56.7818
  78.6613              94.3774             115.5072
 128.2767             153.2958             159.3319
 184.8970             209.6304             211.9815
 241.5386             255.5738             267.6972
 284.8553             299.0893             324.3367
 351.1047             369.2069             400.2868
 400.6100             411.7942             421.1481
 421.9259             452.9888             471.2830
 483.4642             497.2214             504.1755
 508.2020             524.7136             525.7998
 540.3373             546.0249             551.9719
 559.8021             590.3548             596.3114
 597.6598             607.8568             607.8912
 618.4099             625.6778             636.7813
 658.6319             687.6623             693.2565
 694.4395             697.0749             724.4929
 746.7352             754.6936             759.3045
 767.6430             767.7100             774.9047
 778.5477             795.0434             801.6767
 815.8621             818.6370             826.4339
 831.9830             832.3471             858.4047
 871.6331             890.1866             893.7980
 901.7145             917.5535             925.8725
 929.1821             960.9467             976.2249
 977.0361             978.6776             986.4454
 987.6593             991.7394            1000.7327
1033.1701            1070.8846            1093.9732
1107.7028            1118.3504            1141.1339
1154.3498            1165.0860            1176.0774
1194.6713            1201.1342            1206.7716
1222.8737            1226.3014            1245.4920
1250.4274            1251.8569            1264.9412
1285.7786            1297.0085            1310.7549
1329.1542            1347.1102            1357.6794
1367.0210            1369.6828            1391.2549
1402.3589            1406.1716            1427.8439
1436.3349            1447.9051            1455.8542
```

```
1460.3049            1462.1864            1464.8960
1468.9445            1487.7485            1516.3514
1519.0948            1536.8884            1542.0116
1579.9203            1582.3564            1609.6912
1616.2676            1632.8235            1634.7487
1644.7810            1650.1041            1665.1970
1671.4390            2200.9498            3156.4654
3158.9111            3158.9241            3160.2205
3163.3517            3163.4379            3166.3985
3167.4700            3168.8855            3174.9304
3176.9143            3181.0479            3184.3944
3185.2313            3205.5185            3474.9456
       ZeroEnergy[kcal/mol]           0.
       ElectronicLevels[1/cm]      1
         0    1
       End
     Fragment     H
       Atom
         Mass[amu]   1
         ElectronicLevels[1/cm]      1
            0    2
       End
     GroundEnergy[kcal/mol]            -9.4
   End
   Barrier      B1   R   i1    #  ts1
     RRHO
       Geometry[angstrom]     53        #
 C   -3.4390416656   -1.8695997319   -0.0388958778
 C   -4.2268035543   -0.6926951582   -0.0174086215
 C   -3.5439406845   0.5581768086   -0.0067924587
 C   -2.1594214782   0.5724462781   -0.0124246625
 C   -1.3530891342   -0.5901358321   -0.0285129697
 C   -2.0568786303   -1.7846661568   -0.0524753612
 C   -4.2260952667   1.8024075748   0.0106953064
 C   -3.4712861485   3.0175765886   0.0190131929
 C   -2.0267284276   2.9790319153   0.0103627379
 C   -1.3679590418   1.7766560015   -0.0041583064
 C   -5.661751773   -0.6503151785   -0.0072955107
 C   -6.3306313495   0.5394686781   0.0103406314
 C   -5.6441092555   1.807776769   0.0195252216
 C   -6.3057245793   3.0451624015   0.0365249004
 C   -5.5799690281   4.2346023451   0.0444003564
 C   -4.1866924781   4.2264483541   0.035787448
 C   4.1797173228   4.27189969   0.0099514777
 C   3.474468794   3.0574375752   0.0029785115
 C   4.2408481109   1.8496623058   0.0018049853
 C   5.6583108061   1.8652992021   0.0086779822
 C   6.3098277635   3.1082784424   0.0151649189
 C   5.5733936895   4.2910931209   0.0155811244
 C   3.5692461051   0.600324537   -0.0049837503
```

```
C   4.2557409432   -0.6470484448   -0.0016963619
C   5.6911701528   -0.5934824335   0.0052695323
C   6.3526912958   0.6004958235   0.0095958522
C   2.0288027955   3.0046709518   -0.001098849
C   1.3837325786   1.7956281263   -0.008614239
C   2.1861868147   0.5993277406   -0.0124132295
C   1.4050936176   -0.5693279936   -0.0171785798
C   2.0765615995   -1.7987125441   -0.0073740147
C   3.4777115162   -1.823485352   -0.0016886076
C   0.021992085   -0.0945073759   -0.0229255275
C   0.010250047   1.307535666   -0.0129780662
C   -1.0334050196   -3.8658400543   -0.3203843171
C   -1.0067977253   -4.4949429383   0.7184423898
H   -3.9281064504   -2.838683018   -0.0475060497
H   -1.492017289   3.9235687568   0.0162704132
H   -6.2150427294   -1.5836960457   -0.0143620361
H   -7.4157246926   0.5478633566   0.0175013416
H   -7.3903779562   3.0720393087   0.0434238777
H   -6.1070996781   5.1818522764   0.0575972412
H   -3.6426855796   5.165310217   0.0424183466
H   3.6286923729   5.206654205   0.0112777618
H   7.3942104799   3.1447510556   0.02023344
H   6.0924353071   5.2429261211   0.02088597
H   6.2515671603   -1.5227880606   0.0073596449
H   7.4376784185   0.6158054761   0.0147992638
H   1.4850312269   3.9439756555   0.002806145
H   1.528050525   -2.7331733616   0.0046031477
H   3.9840531856   -2.7832523581   0.005897633
H   -0.9006558007   -3.6300629745   -1.3510354123
H   -1.057683298   -4.8978123127   1.7015610135
      Core     RigidRotor
        SymmetryFactor    0.5
      End
     Tunneling      Eckart
       ImaginaryFrequency[1/cm]    315.0302
       WellDepth[kcal/mol]         2.3
       WellDepth[kcal/mol]         44.3
     End
      Frequencies[1/cm]     152
 20.5429               29.0219
 43.9935               45.0211               75.8005
 93.2696               94.3600              113.4338
132.9787              157.6137              183.0828
199.3274              208.7023              212.7307
245.6812              256.5469              267.4101
288.8286              300.1109              331.2429
380.5227              396.0183              397.9516
409.6573              415.8712              426.6630
453.5837              461.0973              494.8595
496.4606              503.6886              509.8033
```

```
 522.4983                525.4395                529.6036
 544.0691                552.7910                573.1196
 583.8755                591.8545                597.1897
 603.2883                611.1445                611.8448
 618.1825                657.1621                661.7943
 688.7573                688.7840                694.2433
 726.3696                740.0084                751.8882
 754.4404                758.3079                758.7040
 769.6199                770.2433                774.0519
 776.9907                795.8839                813.1911
 813.9594                817.1193                826.7097
 828.3020                836.2457                859.6726
 870.0821                875.2985                889.4094
 900.0619                925.0864                927.1297
 927.9916                966.9412                974.9754
 976.1953                978.3723                985.6746
 985.9701                996.7305               1012.9142
1055.1077               1091.5260               1101.9148
1117.0521               1130.8021               1147.6907
1156.5266               1163.7666               1180.7215
1200.1807               1201.3636               1215.7937
1224.2729               1238.5572               1246.5493
1249.1913               1254.4023               1266.5942
1283.6146               1305.8727               1331.4743
1337.3736               1354.0892               1358.2245
1369.1062               1389.6056               1397.2802
1405.6487               1424.6697               1431.8198
1444.6705               1451.9037               1457.8645
1458.3384               1463.7824               1476.1950
1487.2733               1507.8384               1518.0678
1523.0044               1541.1423               1554.6561
1582.4626               1608.0155               1616.3920
1631.4611               1635.0086               1642.6359
1650.0914               1654.6905               1670.7787
1940.4677               3156.5772               3156.7227
3158.1464               3159.4096               3159.6594
3162.9825               3163.6014               3164.9313
3167.7630               3168.1397               3175.1931
3175.5494               3184.8413               3185.2338
3189.2323               3394.2479               3481.8551
        ZeroEnergy[kcal/mol]          2.3
        ElectronicLevels[1/cm]         1
           0   2
   End
     Barrier    B2   i1   p2    #  ts2
     RRHO
       Geometry[angstrom]     53        #
 C   -3.4849798024   -1.8231078005   0.0768156423
 C   -4.2562536787   -0.6496010746   0.0561672518
 C   -3.5680893003   0.59584503   0.0273371558
```

C	-2.1853539615	0.5910045037	0.0211195469
C	-1.3953886888	-0.5725137042	0.0387730705
C	-2.0722476934	-1.8099062856	0.0702795145
C	-4.2412672993	1.8443730184	0.0063784141
C	-3.4766945171	3.0519570091	-0.0200389917
C	-2.0316506187	2.9981399387	-0.0247970238
C	-1.3860852359	1.7897419738	-0.0039545288
C	-5.6926707002	-0.5992659486	0.0622622527
C	-6.3533291173	0.5940107816	0.0415004713
C	-5.6584096536	1.8586043391	0.013177139
C	-6.3112487397	3.1007345221	-0.0076841729
C	-5.5759846446	4.2838688144	-0.0339739026
C	-4.1822012503	4.2658271112	-0.0400547006
C	4.1632801031	4.2575904964	-0.0408290307
C	3.4540797487	3.0452031889	-0.0206687036
C	4.2158078008	1.8346444111	0.0046450765
C	5.633581217	1.8460659441	0.0096278448
C	6.288842916	3.086691743	-0.0112876802
C	5.5566420732	4.2721771516	-0.0361366755
C	3.5404154391	0.5877901109	0.0255500001
C	4.2236742058	-0.6607103145	0.051949628
C	5.6591326057	-0.6118932727	0.0561413189
C	6.3244139483	0.5797461791	0.0359565202
C	2.0092672635	2.9970226143	-0.0246070997
C	1.3608321982	1.789428951	-0.0043635744
C	2.156828601	0.5901793636	0.0205497537
C	1.3705519501	-0.5750914657	0.0398835485
C	2.0410538761	-1.8053183039	0.066035751
C	3.4416228163	-1.8339112305	0.0717413882
C	-0.0127308921	-0.0944649613	0.0265692401
C	-0.0132010203	1.3085025496	0.0012592903
C	-1.3868016134	-3.0753412275	0.1106445482
C	-1.0100148392	-4.1771891606	0.4663312781
H	-3.9836485097	-2.7850291131	0.0983276266
H	-1.4871934571	3.9366735307	-0.0452412203
H	-6.251238229	-1.5290942528	0.0835146213
H	-7.4382139303	0.6096858268	0.0462687367
H	-7.3955724	3.1362718768	-0.0032433333
H	-6.0958831389	5.2350405753	-0.0498569835
H	-3.6317418991	5.2006388872	-0.0604159747
H	3.6151465303	5.1938302143	-0.0602979763
H	7.373345739	3.1195871907	-0.008080428
H	6.079185185	5.2219392994	-0.0520771869
H	6.21638829	-1.5428539303	0.0755605458
H	7.409423749	0.5920026387	0.0394491977
H	1.4680113901	3.9375798645	-0.0438535473
H	1.4893493598	-2.7363232108	0.080266075
H	3.9447672317	-2.7951679548	0.0911781877
H	-0.6256452528	-2.9365813342	-1.6155776709
H	-0.5986281542	-5.1451651039	0.6225227696

```
        Core     RigidRotor
          SymmetryFactor    0.5
        End
       Tunneling      Eckart
         ImaginaryFrequency[1/cm]    790.0144
         WellDepth[kcal/mol]         39.8
         WellDepth[kcal/mol]         7.2
       End
        Frequencies[1/cm]     152
  28.9729               38.8273
  57.0000               75.0480                94.1703
 104.7545              118.3845               155.8907
 159.8407              174.6010               184.6528
 209.7114              215.5509               242.9654
 255.7519              272.1414               289.1803
 299.0644              326.1223               346.5812
 375.0202              400.1908               401.8681
 412.1283              422.1535               431.4131
 453.1611              462.3681               474.1052
 484.4453              497.0592               504.2124
 508.3716              525.7300               526.0072
 542.8409              548.1233               551.9464
 559.8535              591.7835               596.0119
 597.6647              606.1969               608.0873
 618.4006              625.8537               641.2126
 664.0332              687.6205               692.6299
 693.5933              694.8995               724.9813
 748.0014              754.5332               759.3484
 767.1742              768.9377               776.7270
 779.6744              794.4542               803.2610
 815.6978              818.4882               826.2981
 832.1429              832.6424               858.1060
 871.8300              890.3925               895.6123
 901.9085              916.5701               925.8181
 929.2625              962.3736               976.1530
 976.7732              978.4717               986.2690
 987.4971              990.1388              1000.5954
1031.3203             1070.4472              1093.7724
1107.7279             1118.2221              1141.1811
1154.1785             1165.1255              1175.5391
1193.6539             1201.1196              1205.7872
1222.7160             1226.3258              1245.0528
1250.3565             1251.7505              1264.4453
1283.5992             1297.6280              1309.2630
1329.6424             1347.2032              1357.9611
1367.2863             1370.3348              1391.5352
1402.2137             1406.4480              1428.2798
1437.9474             1447.6498              1456.2616
1460.0858             1461.6429              1465.0740
1469.2102             1487.6975              1516.4726
```

```
1518.9914             1536.8466             1542.2313
1580.1544             1583.1470             1610.6711
1616.5435             1633.0885             1635.1567
1645.1225             1650.4110             1665.6477
1671.9132             2098.1908             3157.1166
3159.4587             3159.7218             3160.9270
3163.8622             3164.6332             3166.6924
3168.1859             3169.3572             3175.5653
3177.4806             3183.6272             3185.1696
3186.0248             3204.4071             3459.5601
       ZeroEnergy[kcal/mol]           -2.2
       ElectronicLevels[1/cm]        1
           0   2
   End
   Barrier      B3   i1   i2    #  ts3
     RRHO
       Geometry[angstrom]     53        #
 C   -3.4975097851   -2.4335698788   -0.6800936578
 C   -4.2887331067   -1.264633153   -0.6626007065
 C   -3.6464383532   -0.0180469807   -0.4039912444
 C   -2.302873281   -0.0270054277   -0.0898857339
 C   -1.5514878368   -1.2022602732   -0.0051667222
 C   -2.1117251573   -2.4294943624   -0.4010874412
 C   -4.2967044288   1.2342434727   -0.519806305
 C   -3.5319365666   2.4336407653   -0.3566072576
 C   -2.104604851   2.3707330296   -0.1090743095
 C   -1.4754625486   1.1580348683   0.0220591863
 C   -5.6931305396   -1.2009628258   -0.9640160037
 C   -6.3494425916   -0.0044738661   -1.0275134688
 C   -5.6797062262   1.2581791992   -0.8250542507
 C   -6.3128966469   2.5066306017   -0.9335584978
 C   -5.5847643791   3.6816274468   -0.760363306
 C   -4.2173791618   3.6522187303   -0.484426635
 C   4.3930109153   3.0035446104   -0.4802951804
 C   3.5419469942   1.9059832925   -0.2701644359
 C   4.1446180935   0.6062323424   -0.2562436916
 C   5.5347320652   0.4192313424   -0.4591680761
 C   6.3338442678   1.5524125831   -0.6648336326
 C   5.7624854262   2.8249391229   -0.6714748591
 C   3.3283544513   -0.5281043078   -0.0412578218
 C   3.8229298353   -1.8746726627   -0.0362838399
 C   5.2417110819   -2.0194463495   -0.2455091888
 C   6.0440876628   -0.9336789321   -0.4426638372
 C   2.1111490122   2.0616855608   -0.0918803403
 C   1.3115348643   0.9623741825   0.0947510869
 C   1.9754490967   -0.3262768097   0.1382898609
 C   1.0712974856   -1.3713713069   0.3243087549
 C   1.5274955519   -2.7134248846   0.4534461345
 C   2.9312693211   -2.9245063733   0.23194487
 C   -0.2126666238   -0.7623351537   0.2642671508
```

```
C   -0.1164551931   0.6386318229   0.2000441032
C   -1.2898614732   -3.6161734497   -0.7122364682
C   0.0294430513   -3.8054926112   -0.6987280315
H   -3.964440326   -3.3690633814   -0.9736650377
H   -1.5589631812   3.3072501219   -0.0514928355
H   -6.2339542722   -2.1231903927   -1.1501952565
H   -7.4098490178   0.0141397037   -1.2571057439
H   -7.3726841524   2.5537991942   -1.1612553696
H   -6.0872656281   4.6381179416   -0.8502448891
H   -3.6725844559   4.5836999291   -0.3703706554
H   3.9750047083   4.004783941   -0.4963381432
H   7.4014837271   1.4369511428   -0.8198896626
H   6.3937641948   3.6916622351   -0.8314286091
H   5.6726484416   -3.0153639609   -0.2443841053
H   7.108814689   -1.0757173082   -0.5972357337
H   1.7039719732   3.0671924375   -0.128062399
H   1.0368238884   -3.3834562052   1.1509570095
H   3.329992666   -3.9275608349   0.344505496
H   -1.8734142264   -4.4498787336   -1.1182968366
H   0.5457185453   -4.6999851949   -1.0247314323
      Core     RigidRotor
        SymmetryFactor    0.5
      End
      Tunneling      Eckart
        ImaginaryFrequency[1/cm]    540.3372
        WellDepth[kcal/mol]         19.0
        WellDepth[kcal/mol]         30.0
      End
      Frequencies[1/cm]     152
 31.5568               45.9789
 80.6904               91.4103               103.2022
141.6299              158.7300              177.1841
184.8941              206.6607              236.3002
252.4022              255.8834              273.7568
288.6781              299.9055              317.3279
347.8105              361.3616              383.7876
395.4310              402.8706              411.7250
422.1847              459.1745              468.3761
489.2636              499.6404              500.7572
507.0303              515.3347              525.3437
533.3542              539.0566              554.1021
566.4321              583.1914              587.1781
597.8988              603.2082              605.5830
613.4157              618.2033              668.5769
674.1957              690.5590              694.7120
708.8594              723.2660              742.6621
745.7294              759.5300              765.1544
770.1472              772.2086              781.0560
786.8890              799.9800              803.0720
808.9433              825.6629              829.6760
```

```
 831.9777             851.8237             865.8107
 876.7482             879.8486             891.3420
 898.8064             904.6126             912.1073
 924.4399             926.8539             969.0766
 973.2159             974.3445             979.2621
 983.6806             983.8195             996.8379
1009.2369            1059.9195            1079.8333
1092.9579            1107.6947            1112.9013
1123.1180            1142.3833            1158.5802
1163.8219            1182.8235            1199.4444
1201.7040            1203.1687            1217.5042
1233.8464            1246.8707            1247.6880
1255.3398            1270.1969            1284.3293
1294.4621            1306.7618            1317.6267
1338.6986            1346.2103            1365.7394
1370.0819            1391.0552            1397.0442
1409.6029            1418.7071            1428.4709
1439.9417            1447.9073            1454.5531
1455.5716            1460.1640            1466.3158
1509.5818            1512.4522            1517.4100
1529.4733            1538.5139            1573.1320
1587.1624            1594.3759            1598.3294
1618.6537            1624.2097            1625.5757
1633.6262            1644.6515            1659.2146
1673.1047            3030.1631            3136.4238
3155.1054            3156.3459            3157.1670
3159.1364            3159.7707            3162.9545
3164.4452            3164.5537            3167.7483
3168.1339            3174.7562            3174.8425
3184.4391            3185.3447            3191.5264
        ZeroEnergy[kcal/mol]          -23.0
        ElectronicLevels[1/cm]        1
          0   2
   End
   Barrier      B4   i2   p1    #  ts4
     RRHO
       Geometry[angstrom]     53        #
 C   -3.3726174308   -1.7971700655   0.0509434336
 C   -4.1976014267   -0.6472723285   -0.0069333954
 C   -3.6102946383   0.6484354343   0.1581629577
 C   -2.259440487   0.7209079083   0.4100959854
 C   -1.4853717188   -0.4244893001   0.4969440755
 C   -1.9751537348   -1.7164545561   0.2842367275
 C   -4.3134104459   1.8655595989   0.0313645276
 C   -3.5872507592   3.0971889838   0.1472509207
 C   -2.1504849821   3.1094196928   0.3682416405
 C   -1.4539690091   1.9314469363   0.4977874804
 C   -5.6143337619   -0.6474393162   -0.2633081831
 C   -6.320485125   0.5192184138   -0.3592482018
 C   -5.7050133415   1.8201217846   -0.2272567809
```

```
C   -6.3924561124   3.036246931   -0.3502702091
C   -5.7055206145   4.2440245972   -0.2288570289
C   -4.3316613563   4.2808920307   0.0100562953
C   4.6123379566   3.6071900498   0.3449751502
C   3.692825075   2.54866003   0.4428102194
C   4.2340631012   1.2211245435   0.3953588336
C   5.6175660068   0.9673611948   0.2272690414
C   6.4839313671   2.0648059448   0.1386527664
C   5.9777329587   3.3640292827   0.2025612142
C   3.3513462625   0.1242507287   0.4882893167
C   3.7510926033   -1.2506436853   0.3626813096
C   5.1680578771   -1.4610421423   0.1920562125
C   6.0415477418   -0.4131058692   0.1392883379
C   2.2618223146   2.7769340592   0.5491226397
C   1.3900080698   1.7177080007   0.6328511565
C   2.0111757536   0.3994749132   0.6330050149
C   1.0660249107   -0.613363297   0.6543584328
C   1.3766813798   -1.9884991268   0.5845839895
C   2.7769428878   -2.2645358799   0.3761407389
C   -0.169504454   0.0468562576   0.665212087
C   -0.06513025   1.4403780097   0.6558284769
C   -1.0613981427   -2.8681122453   0.2381206194
C   0.2983309917   -2.9817560584   0.3461551252
H   -3.8283583593   -2.7687008296   -0.115470737
H   -1.6580199382   4.0758532232   0.4083350698
H   -6.126839292   -1.5960325542   -0.3866853381
H   -7.3874715254   0.4815940545   -0.5536377655
H   -7.4598454719   3.036633708   -0.5446234672
H   -6.2497290458   5.1765503959   -0.3279545879
H   -3.8275761925   5.2386081444   0.0882475424
H   4.2509841347   4.6298840386   0.3754876405
H   7.5495527037   1.9037316342   0.0139428344
H   6.6598377498   4.203739129   0.1310896524
H   5.541070906   -2.4762257332   0.1040550645
H   7.1015565729   -0.6089931009   0.0140431079
H   1.9161453044   3.8057429227   0.5359191966
H   1.4226818266   -2.374539741   2.3423735415
H   3.0942169685   -3.2959541784   0.2596146589
H   -1.5675450579   -3.8130001286   0.0536228492
H   0.6728172493   -3.9973104403   0.24478781
      Core     RigidRotor
        SymmetryFactor    0.5
      End
      Tunneling      Eckart
        ImaginaryFrequency[1/cm]    968.3680
        WellDepth[kcal/mol]         24.9
        WellDepth[kcal/mol]         8.5
      End
      Frequencies[1/cm]     152
 22.6987                48.9809
```

87.9745	92.3962	114.7286
147.0110	164.3349	182.0918
189.1300	209.6600	252.2863
255.6744	266.2702	275.6696
298.3645	329.7773	335.5960
350.6951	364.6252	379.0620
390.2345	411.4578	417.4001
432.1277	437.9933	465.6541
469.4026	500.5160	502.2992
507.4980	516.1460	523.2641
525.3436	532.7812	546.7074
551.4770	558.9453	566.2188
593.9433	595.3007	603.2609
607.7218	614.7519	617.9638
621.1998	692.3194	694.8926
695.7540	719.9539	743.5651
746.0303	757.5141	758.8276
766.7023	768.9040	772.6400
778.9985	801.4598	806.3384
812.1226	814.1811	823.6873
832.0485	835.5880	838.3176
875.8025	879.8236	887.1755
892.9121	893.5733	899.8438
925.2470	926.9679	973.1582
974.2578	974.6085	975.2776
985.1721	985.2503	999.8474
1002.0678	1019.0253	1037.8543
1083.2006	1091.7916	1106.4633
1129.0581	1137.9610	1157.7633
1165.0631	1180.5991	1191.3116
1199.6924	1202.5820	1218.5305
1237.0753	1246.4163	1247.3201
1258.2016	1272.0773	1291.2781
1300.3703	1302.9314	1308.3867
1331.9679	1344.8096	1363.2791
1387.4181	1388.9138	1396.9952
1407.6693	1430.4469	1432.4113
1438.6646	1442.0123	1449.0763
1455.6594	1457.0568	1462.8592
1477.2149	1510.3439	1511.2754
1520.9995	1544.2693	1566.6090
1572.5118	1585.4704	1594.4218
1616.0462	1617.8020	1632.8636
1636.7823	1643.5698	1658.1528
1683.6944	1689.3660	3125.5589
3146.8813	3155.6377	3156.6426
3158.4398	3159.4606	3160.0206
3163.5177	3163.7305	3164.5457
3168.3078	3168.7624	3174.1015
3174.9015	3184.1922	3184.8725

```
      ZeroEnergy[kcal/mol]          -28.1
      ElectronicLevels[1/cm]        1
         0   2
  End
  Barrier      B5   i1   i3    #  ts5
    RRHO
      Geometry[angstrom]     53        #
C   -3.4102682955   -1.9301687437   -0.0103115432
C   -4.213086567   -0.7763010227   -0.0064493049
C   -3.5596406307   0.4874971842   -0.0018499011
C   -2.1777052125   0.5115445075   -0.0014170415
C   -1.370382946   -0.6391625627   -0.0052535367
C   -1.9948678422   -1.9090157869   -0.0098843015
C   -4.2608722187   1.7188487993   0.0022988074
C   -3.5230759761   2.9436751618   0.0067797583
C   -2.0767311973   2.923383242   0.0070671001
C   -1.4006271643   1.7310072805   0.0030550987
C   -5.6504141898   -0.7599766696   -0.0067451252
C   -6.3407879593   0.41728883   -0.00278234
C   -5.6780949325   1.6993159437   0.0018805934
C   -6.3596084566   2.9260203452   0.0060166208
C   -5.6513087427   4.1259984452   0.0104019813
C   -4.2572415822   4.1406105248   0.0107976281
C   4.2503861658   4.1320996656   0.0132251778
C   3.5072088961   2.9400607322   0.0087900761
C   4.2420861623   1.7108446823   0.0047194558
C   5.6588427065   1.6828067686   0.0051049624
C   6.3468807398   2.9057505818   0.0096293232
C   5.6442201292   4.109299741   0.013614698
C   3.5349101894   0.483266626   0.0001737179
C   4.1769649866   -0.791475774   -0.0040604371
C   5.6135644187   -0.7792686802   -0.0035280563
C   6.3109177236   0.3953799906   0.0008177178
C   2.0584625877   2.9361273736   0.0083008379
C   1.371474675   1.7485287537   0.0039024103
C   2.1550720651   0.5380716487   -0.0001023078
C   1.3485948878   -0.6069597304   -0.0043636187
C   1.9744729236   -1.8415848119   -0.0084710432
C   3.3625374656   -1.9534851858   -0.0083901112
C   -0.0139865158   -0.1229152063   -0.0030919982
C   -0.0150346056   1.2792534436   0.001827421
C   -1.3179697704   -3.2171875295   -0.0143387169
C   -0.0365314864   -3.583304882   -0.0152579655
H   -3.9034785508   -2.8972373667   -0.0138408805
H   -1.5547114229   3.8749372851   0.0105290162
H   -6.1874328137   -1.7028714935   -0.010185834
H   -7.4257931208   0.4055733885   -0.0030897211
H   -7.4445134752   2.936751948   0.0057860642
H   -6.1929358866   5.0651393491   0.0135631639
H   -3.7292112893   5.0885221078   0.0142535946
```

```
 H   3.7288296756   5.0835902031   0.0163908099
 H   7.4318523515   2.9114226001   0.0100178553
 H   6.1907591414   5.0456073179   0.0170849673
 H   6.1474993829   -1.7239493089   -0.0066500278
 H   7.3958841276   0.3754128538   0.0011152392
 H   1.5492969812   3.8946975036   0.0115097823
 H   1.0515167253   -2.7977013253   -0.0121408559
 H   3.8386122496   -2.9290248374   -0.0116376289
 H   -2.0277942343   -4.0489339813   -0.0175073072
 H   0.2880767275   -4.61912593   -0.0188492751
       Core     RigidRotor
         SymmetryFactor    0.5
       End
       Tunneling      Eckart
         ImaginaryFrequency[1/cm]    1655.1684
         WellDepth[kcal/mol]         13.7
         WellDepth[kcal/mol]         12.3
       End
       Frequencies[1/cm]     152
  27.4671               44.8762
  72.7408               93.5014               98.0882
 130.5218              153.5760              161.9140
 183.5762              210.2845              228.6895
 237.0014              246.7185              267.9752
 293.8779              297.9927              313.0376
 315.3164              347.1223              368.6683
 392.0423              394.7189              410.4656
 420.0405              458.1880              465.5539
 465.9102              491.3750              500.3046
 503.6914              505.4844              510.7574
 525.6614              535.4555              542.3105
 554.4683              568.5044              581.1160
 598.1259              605.8832              606.6275
 609.0601              617.2065              619.2069
 675.2119              690.6779              693.4462
 699.9677              709.1827              735.8181
 746.4852              752.9262              767.7262
 768.7473              770.2289              776.7001
 789.7507              797.8813              802.0133
 808.7002              825.9779              829.1298
 832.6427              834.6451              869.1880
 876.4760              882.7283              891.2726
 901.1560              912.3897              926.0508
 928.7582              932.0303              974.8576
 974.8634              975.3563              986.0212
 986.2653              990.1025              999.6267
1020.0491             1049.5136             1071.1549
1090.3865             1099.4070             1109.3830
1117.1185             1149.4532             1163.0643
1165.2694             1189.7387             1199.5925
```

```
1201.5342            1205.3192            1223.8596
1243.9143            1247.8134            1249.9146
1258.7769            1280.1721            1286.5240
1297.9033            1308.6165            1337.3816
1343.1092            1351.7504            1367.3711
1370.2342            1389.1391            1399.0739
1403.4524            1416.4244            1426.0455
1444.8904            1451.4002            1454.7328
1462.8162            1464.9678            1469.4709
1481.9722            1501.7188            1516.0322
1518.5540            1532.5053            1542.0512
1576.0692            1582.0349            1608.0789
1614.6556            1628.6735            1631.1824
1641.4260            1649.3993            1654.7406
1663.7346            1672.2191            3056.3099
3156.9266            3157.2793            3159.3626
3159.5883            3159.8005            3160.1217
3162.9546            3164.9456            3165.5986
3168.2844            3168.5741            3175.2919
3176.0880            3185.0303            3185.4380
        ZeroEnergy[kcal/mol]          -28.3
        ElectronicLevels[1/cm]        1
           0   2
   End
   Barrier      B6   i3   i4    #  ts6
     RRHO
       Geometry[angstrom]     53        #
 C   -3.7629798399   -2.4408129287   0.1543093578
 C   -4.4685382993   -1.2225618721   0.2265651566
 C   -3.7318881013   -0.0166246261   0.4350401345
 C   -2.362873971   -0.1003610951   0.5792201775
 C   -1.671955138   -1.3143576552   0.5384751766
 C   -2.3590509061   -2.5235742952   0.3205077924
 C   -4.3241529923   1.2693221999   0.4464324161
 C   -3.4849872791   2.42086558   0.5846352403
 C   -2.0448769342   2.279298738   0.6820855725
 C   -1.4684871136   1.0349442824   0.6702096222
 C   -5.8923331753   -1.0738730025   0.0804789398
 C   -6.4839995034   0.1558985606   0.1232376105
 C   -5.7289308469   1.3747266786   0.2974788722
 C   -6.3032058402   2.6548273821   0.3143062184
 C   -5.5000213242   3.7841436737   0.4626908386
 C   -4.1154689884   3.6755587599   0.590783446
 C   4.4886665308   2.7011939947   0.4989907564
 C   3.5924897612   1.6202480514   0.4927308892
 C   4.1532990356   0.3081213992   0.3669513464
 C   5.5487936466   0.0938560656   0.2513463259
 C   6.3950971852   1.21397316   0.2635147487
 C   5.8637677725   2.4958898387   0.3860195636
 C   3.2880138253   -0.8133455082   0.3562858988
```

```
C   3.7449973289   -2.1602397801   0.2341010737
C   5.1659346009   -2.3364383773   0.1194196071
C   6.0156767567   -1.2657695791   0.128113435
C   2.1586550138   1.8081249426   0.6084469056
C   1.3148001741   0.7264571354   0.5956188612
C   1.9329375739   -0.5753143382   0.4664484629
C   0.9860578385   -1.6078478887   0.471501653
C   1.431091612   -2.9099149269   0.3637465849
C   2.7812665919   -3.2097634908   0.2414278527
C   -0.2842263919   -0.9474323636   0.5934302633
C   -0.1252270757   0.4414249611   0.6678895918
C   -1.6151539671   -3.7795298425   0.2070175171
C   -0.4860479981   -4.0964026301   0.9246103603
H   -4.3208183301   -3.3544205163   -0.027494562
H   -1.4508546987   3.1855475605   0.7452029724
H   -6.5006308436   -1.9601553284   -0.0680558718
H   -7.5603437272   0.2373810542   0.0119271815
H   -7.3773042495   2.763736721   0.2063714156
H   -5.9584365992   4.7665734391   0.4722237943
H   -3.5137959583   4.5727818336   0.6928170693
H   4.1029121538   3.7110428893   0.5932014333
H   7.468044501   1.0776163284   0.1771889305
H   6.5304017914   3.3508439556   0.3939573936
H   5.5656752824   -3.340798044   0.0241345246
H   7.084764328   -1.4297746397   0.0395490649
H   1.7882916496   2.8240850937   0.703340555
H   3.1253452406   -4.2361280665   0.156992705
H   -1.9811393762   -4.495546304   -0.5249536913
H   -0.2717382864   -3.5749453998   1.8478937641
H   -0.0546454395   -5.08429178   0.8228840516
      Core     RigidRotor
        SymmetryFactor    0.5
      End
      Tunneling      Eckart
        ImaginaryFrequency[1/cm]    350.9306
        WellDepth[kcal/mol]         6.0
        WellDepth[kcal/mol]         37.7
      End
      Frequencies[1/cm]     152
 27.6285              46.9654
 78.9304              92.0621             112.1357
141.1394             153.8516             166.6640
182.8273             210.2533             238.6182
246.4848             258.1661             268.3961
287.0771             292.1478             314.0418
334.2019             362.9621             388.0596
390.4474             398.4637             408.2809
419.1017             448.5899             469.0379
471.5037             497.6275             503.8144
508.4100             523.8855             525.4247
```

```
 533.3381              538.4693              554.5615
 567.3585              579.3521              589.4587
 595.8822              606.1893              609.4613
 613.5346              617.3254              649.9770
 661.4622              688.6913              694.4726
 722.0736              730.3190              745.7264
 749.8929              762.4652              766.8354
 769.1513              773.7349              780.5524
 785.2280              798.5609              800.7345
 807.9249              827.9383              831.8107
 833.7155              865.5935              872.7292
 886.4879              892.9959              897.1028
 900.9136              920.3064              926.0282
 928.4557              941.3716              973.6853
 974.6785              977.9128              984.6428
 984.9861              986.6692              998.7512
1015.3013             1065.7152             1090.6566
1099.3424             1110.1240             1114.1768
1144.2698             1155.6321             1163.3698
1180.4071             1198.7946             1199.9599
1202.5603             1218.1074             1236.9263
1246.4564             1248.7160             1251.7275
1266.3544             1283.2320             1288.1838
1304.0767             1313.1439             1334.1821
1343.5200             1356.6487             1372.5402
1385.0902             1396.0084             1403.2461
1423.8301             1424.0176             1431.0136
1443.9169             1450.1608             1455.1052
1459.7085             1461.2735             1471.0679
1499.5761             1510.8228             1514.6064
1526.5695             1537.8032             1542.7498
1573.0057             1585.5818             1610.4728
1612.9094             1626.3973             1628.2203
1642.1617             1646.2062             1660.3117
1674.1541             3136.4522             3148.2773
3153.6661             3156.2746             3156.3400
3158.9705             3159.0708             3159.6391
3162.1624             3164.2266             3167.7051
3168.1494             3174.2831             3174.9672
3184.5395             3184.8992             3236.5199
        ZeroEnergy[kcal/mol]          -34.6
        ElectronicLevels[1/cm]        1
           0   2
   End
   Barrier      B7   i4   p1    #  ts7
     RRHO
       Geometry[angstrom]     53        #
 C   -3.3160267457   -2.1225860483   -0.0820334207
 C   -4.0988932299   -0.9439303808   -0.0659443277
 C   -3.4534358163   0.3318817413   0.0406671144
```

C	-2.0823304053	0.3582351143	0.141977438
C	-1.3440970112	-0.8129301907	0.1355701383
C	-1.896260243	-2.0923379418	0.00818594
C	-4.1250576658	1.5725172125	0.0211579065
C	-3.3495754919	2.7769268772	0.0966310544
C	-1.8978270833	2.7396888323	0.1794945422
C	-1.230299487	1.5387067446	0.2003056974
C	-5.5348193634	-0.8927363162	-0.1683793625
C	-6.2062818057	0.2978216102	-0.1736567197
C	-5.5370223227	1.5763540266	-0.0850769367
C	-6.1926188532	2.8155108551	-0.1039570185
C	-5.4563739099	3.9977541508	-0.0247593954
C	-4.0647613448	3.9859828462	0.0712378123
C	4.9287783345	2.964512378	0.053099361
C	3.9604774488	1.9467063104	0.0814030426
C	4.4459662442	0.5989388609	0.0072851591
C	5.8226842111	0.2854839919	-0.0989757697
C	6.7394949985	1.3460832652	-0.1211490366
C	6.2873284721	2.6635318136	-0.0442734044
C	3.5133332758	-0.4594839277	0.0302840369
C	3.8557594534	-1.8473631047	-0.0676516261
C	5.2658600284	-2.1203767448	-0.1711002309
C	6.1876548716	-1.1107691667	-0.1826072802
C	2.5378397672	2.2360224016	0.168538497
C	1.617899697	1.2151312791	0.1914828233
C	2.183169351	-0.1260852902	0.130587334
C	1.2019463512	-1.1031397474	0.133095002
C	1.4551925762	-2.4715299228	0.022806874
C	2.827092933	-2.8213493697	-0.0699138878
C	-0.0068418495	-0.3897008397	0.1995660745
C	0.1506455302	0.995334024	0.2331185143
C	-1.0368777689	-3.2723044826	-0.0590628953
C	0.3405872973	-3.4386005341	-0.0066652612
H	-3.8219088973	-3.0785297731	-0.1787636838
H	-1.3742787173	3.6898886296	0.2132246924
H	-6.0901440065	-1.8218696734	-0.2454455192
H	-7.2885995458	0.2982097835	-0.252681151
H	-7.2740144236	2.8543543167	-0.1822694926
H	-5.9765737877	4.9489171659	-0.0412653998
H	-3.5243174232	4.9253685464	0.1249824297
H	4.6130008186	4.0012915652	0.106030587
H	7.8020110373	1.1412137888	-0.1997161846
H	7.007692565	3.4736726969	-0.063119868
H	5.5985054097	-3.1508231681	-0.2427763274
H	7.2423238903	-1.3538243798	-0.2617471584
H	2.2408588639	3.2793759484	0.2052759153
H	3.1060629582	-3.8676572074	-0.15009678
H	-1.5843133107	-4.2073579886	-0.1508887753
H	0.4644039833	-4.0519385126	1.9917751979
H	0.6623571416	-4.4655120662	-0.1557262713

```
       Core     RigidRotor
         SymmetryFactor    0.5
       End
       Tunneling      Eckart
         ImaginaryFrequency[1/cm]    536.8812
         WellDepth[kcal/mol]         41.1
         WellDepth[kcal/mol]         5.4
       End
       Frequencies[1/cm]     152
  15.8114                49.1715
  88.4862                90.5700                112.3836
 143.0791               162.7227                177.6143
 181.8836               208.9130                231.6260
 252.2390               255.3557                269.5613
 278.4002               289.7222                307.0676
 343.1373               346.9034                362.6304
 370.3440               381.7760                410.8017
 428.9394               429.6350                454.8629
 466.5444               500.4964                501.2823
 508.3237               515.6775                523.0243
 524.7260               534.7744                547.3616
 552.3101               559.4802                563.8218
 595.8489               597.2596                601.9207
 608.9196               614.3873                617.8187
 620.5976               686.9242                694.5680
 695.9040               720.1574                746.1229
 746.8924               755.1407                757.8106
 767.8296               773.8466                774.7993
 778.7491               802.5784                805.5395
 812.3738               820.1917                825.2602
 832.2531               836.7433                840.6505
 877.1405               881.6113                890.1183
 891.5483               894.7870                900.8648
 926.8848               928.6744                972.4236
 974.9441               975.1003                975.1785
 986.0945               986.2002                995.8216
1003.7544              1018.9189               1043.3261
1082.9794              1091.9595               1106.5297
1129.0220              1137.8821               1160.6699
1165.0580              1184.1144               1195.3455
1199.8185              1202.6061               1218.2770
1240.5631              1245.6501               1247.2505
1256.1255              1274.9078               1288.4167
1296.6024              1302.5820               1309.5820
1333.2803              1345.0147               1368.1929
1389.0853              1393.0307               1399.4088
1407.4151              1425.5811               1439.8111
1441.0008              1443.5077               1450.6700
1456.2199              1456.5539               1468.0346
1480.6736              1502.9608               1511.2872
```

```
1523.1228              1529.3770              1549.3620
1574.1425              1586.4100              1595.9566
1617.6245              1618.8805              1636.4164
1638.3740              1655.6641              1663.6753
1691.5915              1693.6212              3130.9454
3150.6165              3156.2042              3156.2558
3159.4661              3159.6071              3159.8194
3160.0187              3163.6237              3164.5840
3168.5316              3168.7100              3174.5650
3174.6564              3184.0766              3184.2488
        ZeroEnergy[kcal/mol]          -31.2
        ElectronicLevels[1/cm]        1
           0   2
   End
   Barrier      B8   i2   i4    #  ts8
     RRHO
       Geometry[angstrom]     53        #
 C   -3.5833549698   -2.4912375151   -0.0610829812
 C   -4.3559009487   -1.3040124117   -0.0670078039
 C   -3.7018137241   -0.0372948018   0.0579506931
 C   -2.3319941832   -0.0291933718   0.206906432
 C   -1.6040464504   -1.2040344161   0.2325297517
 C   -2.1663914744   -2.4808618827   0.0693906985
 C   -4.359491763   1.2102423284   0.0051917959
 C   -3.5764692128   2.4088947025   0.0926294114
 C   -2.1265255069   2.3550738002   0.2182173188
 C   -1.4753143312   1.1484346835   0.2738146226
 C   -5.7860738659   -1.2394997959   -0.2172288772
 C   -6.4460334558   -0.0420125545   -0.2504961235
 C   -5.766943533   1.2285623667   -0.1483196123
 C   -6.4090529216   2.4759970408   -0.1995308953
 C   -5.6651743151   3.6503065028   -0.1077620877
 C   -4.2754674994   3.6237536115   0.0324548804
 C   4.6457204306   2.6102572758   0.0181499821
 C   3.6910649418   1.5817958131   0.1031460942
 C   4.1848795129   0.2358274577   0.0285788447
 C   5.5602922041   -0.0587734279   -0.1491717284
 C   6.46165219   1.0092180705   -0.224795702
 C   6.0012419859   2.3254801424   -0.1370149411
 C   3.2681903417   -0.8298935665   0.1069654672
 C   3.6195925459   -2.221326363   -0.0302468346
 C   5.0313804471   -2.4723296535   -0.210768889
 C   5.9366365007   -1.4521977136   -0.2572344256
 C   2.2706202999   1.8555582056   0.2278829321
 C   1.3628862041   0.8255202642   0.2983639755
 C   1.9362134568   -0.5149561116   0.2619657326
 C   0.9540665678   -1.487245405   0.2861253177
 C   1.2157125392   -2.8849621098   0.1781601074
 C   2.6247419136   -3.2072049554   -0.0216821575
 C   -0.2570518836   -0.7881678156   0.3328430668
```

```
C   -0.0997284329   0.5996996988   0.3479439115
H   -4.0963118921   -3.4405828601   -0.1832524531
H   -1.5918421164   3.2991187616   0.2524196864
H   -6.3478289669   -2.1635278392   -0.3094776355
H   -7.5251364907   -0.0317009418   -0.3656810124
H   -7.4869785866   2.5239690364   -0.3137848623
H   -6.1738397561   4.6068904947   -0.1500250928
H   -3.7262999535   4.5577337556   0.0931275509
H   4.3183995016   3.6436918056   0.0692702644
H   7.5207592707   0.8138996118   -0.3573502324
H   6.7111427329   3.1425045047   -0.1985842791
H   5.3728953254   -3.497424646   -0.312449959
H   6.9889652643   -1.6817827191   -0.3913663025
H   1.9597041257   2.8955428663   0.2420258952
H   2.9125331201   -4.2457088763   -0.1516369725
C   -1.3286051576   -3.6694239285   0.0022163765
C   0.07050724   -3.8475880037   0.031163447
H   -1.871818862   -4.6049127155   -0.0928198884
H   0.7616689678   -3.512076517   1.2319562256
H   0.404879623   -4.8715238828   -0.1063917332
      Core     RigidRotor
        SymmetryFactor    0.5
      End
      Tunneling      Eckart
        ImaginaryFrequency[1/cm]    1611.9527
        WellDepth[kcal/mol]         29.4
        WellDepth[kcal/mol]         48.7
      End
      Frequencies[1/cm]     152
 22.6684                48.7284
 89.5140                91.4407               113.3537
145.6372               162.4465               181.1350
193.0223               206.8813               251.0804
254.0919               261.9079               276.1167
300.9573               330.8603               339.1093
350.4413               366.8803               379.8916
404.5603               419.8911               427.0677
428.3048               463.5700               482.3075
498.7022               503.8540               509.2547
510.3472               524.0755               533.5652
541.8516               544.7462               550.2445
559.9023               591.5394               594.7402
599.4691               603.8683               607.1529
613.9194               618.8884               679.3032
686.7064               692.4184               718.4315
732.2406               740.8818               743.9810
746.1132               756.6407               766.7727
769.4693               770.5197               781.9123
788.8839               798.1474               802.9604
817.8439               819.7247               830.8514
```

```
 833.5899               839.7326               874.0619
 874.6804               886.4553               890.6734
 892.8873               898.8752               924.8511
 926.5562               963.2944               969.9005
 971.2675               971.8464               980.6616
 982.3887               997.9564              1010.5862
1029.2723              1075.3503              1078.9184
1091.6875              1105.0282              1120.4459
1132.9052              1156.9891              1158.3542
1175.7825              1188.3660              1197.2602
1200.4125              1215.5989              1221.8645
1243.3961              1245.7190              1249.9974
1260.7384              1286.9377              1288.6021
1301.9028              1304.7258              1320.3155
1343.8100              1354.6745              1381.4763
1386.3970              1396.1579              1406.2965
1411.4688              1422.6660              1430.2489
1432.8705              1445.1291              1451.2314
1455.2142              1459.4368              1472.8695
1481.6137              1509.1839              1515.1925
1517.7721              1543.1141              1561.1556
1583.8846              1589.6965              1600.4604
1613.5412              1622.6048              1629.0432
1633.4655              1640.6015              1659.0309
1675.6045              1698.2791              3142.9043
3153.8425              3154.0575              3156.5288
3158.5295              3158.6516              3159.6589
3162.2106              3162.4106              3163.2779
3166.6744              3167.1969              3172.4608
3172.8211              3183.5711              3184.3719
        ZeroEnergy[kcal/mol]          -23.6
        ElectronicLevels[1/cm]        1
           0   2
   End
End
```

Input file for RRKM-ME calculations for the $C_{36}H_{15}$ + C_2H_2 reaction

```
TemperatureList[K]                    500. 600. 700. 800. 900. 1000. 1125.
1250. 1375. 1500. 1650. 1800. 2000. 2250. 2500.
PressureList[atm]                    0.01 0.03 0.03947368 0.1 0.3 1. 3. 10.
30. 100.
EnergyStepOverTemperature            0.2          #Ratio of discretization
energy step to T
ExcessEnergyOverTemperature          250
ModelEnergyLimit[kcal/mol]           1500
WellCutoff                           10
ChemicalEigenvalueMax                0.2
ChemicalEigenvalueMin                1.e-6          #only for direct
diagonalization method
CalculationMethod                    direct
EigenvalueOutput                                 eigenvalue.out
Reactant        #ground energy of bimolecular species will be used as a
reference.
Model
  EnergyRelaxation
    Exponential
      Factor[1/cm]                  247    ! Jasper universal
      Power                          0.85
      ExponentCutoff                15
    End
  CollisionFrequency
    LennardJones
         Epsilons[1/cm]          101.5  1283.3  ! N2 chrysene-Frenklach
         Sigmas[angstrom]        3.6154 9.26  ! N2 chrysene-Frenklach
        Masses[amu]            28.  449.13303
    End
OutputTemperatureStep[K]  100
OutputTemperatureSize  20
OutputReferenceEnergy[kcal/mol]  0.
  Well       i1  # i1
  Species
    RRHO
      Geometry[angstrom]    55
 C   -3.5809831688   -2.3309799946   0.8105287137
 C   -4.3461607278   -1.1399697115   0.838767251
 C   -3.6828114269   0.1274363553   0.752571319
 C   -2.3132733754   0.1357237839   0.6214091657
 C   -1.594482111   -1.0472561474   0.5888703315
 C   -2.165155358   -2.3192955632   0.6990161502
 C   -4.3344259285   1.376367686   0.8260019519
 C   -3.5408121772   2.5696872927   0.7752851092
 C   -2.0922578194   2.5135415986   0.6619975506
 C   -1.4431712823   1.3039738688   0.5810064079
 C   -5.7782936274   -1.0705422356   0.9726090989
 C   -6.4312501244   0.1292605444   1.0272856635
```

```
C   -5.743638543   1.3990722324   0.9640665794
C   -6.3778406355   2.6471842166   1.0384091925
C   -5.6236699583   3.8196270299   0.9835558825
C   -4.2348512479   3.7888170969   0.8581331264
C   4.7651018169   2.6080023831   0.6907666465
C   3.7634611378   1.6221095122   0.669082462
C   4.2164438587   0.2609245064   0.7361800285
C   5.5855084028   -0.0873534828   0.8514672812
C   6.5318264573   0.9455312295   0.8860943865
C   6.114922141   2.2713389355   0.8005739109
C   3.2659432409   -0.7806432863   0.6837183117
C   3.579830793   -2.1759774798   0.7744142921
C   4.9814660513   -2.4813012273   0.8926766256
C   5.9218397941   -1.4910694482   0.9255015727
C   2.3326941175   1.9540885226   0.5720649752
C   1.4030189476   0.9303530081   0.5279364315
C   1.9444381234   -0.4197727246   0.5660664996
C   0.9441855156   -1.3784551379   0.5594705982
C   1.1678701337   -2.7541301645   0.6710078926
C   2.5339654754   -3.1298302496   0.7635773428
C   -0.2513391597   -0.6435107735   0.5144104715
C   -0.069495884   0.7399931732   0.5080755379
C   -1.3197443759   -3.5190999854   0.7273628719
C   0.0430277236   -3.6965373393   0.7171399821
C   1.0447847958   3.8798700305   -0.2984364803
C   1.9258995825   3.3824889987   0.5389114703
H   -4.0999115378   -3.2812055881   0.8944288828
H   -1.5520353796   3.4540451238   0.6616529435
H   -6.3460389528   -1.9933059505   1.0346442566
H   -7.511588034   0.1434297483   1.1291841477
H   -7.4565313595   2.7005699685   1.1419186295
H   -6.1274011316   4.7778600567   1.0440255159
H   -3.6792573408   4.7202767165   0.8269248765
H   4.4959850541   3.6534183301   0.6074309779
H   7.587349355   0.7103593836   0.9728839779
H   6.8542415361   3.0642709883   0.8158966261
H   5.290804474   -3.5192992413   0.9591409456
H   6.9709202753   -1.7539082206   1.0145649443
H   2.7925826352   -4.1811743691   0.8471556815
H   -1.8856394449   -4.4461723625   0.7856978949
H   0.3534012253   -4.7376787845   0.7694701573
H   0.4300967695   3.5275883661   -1.1141773545
H   2.4136236791   4.0495927808   1.2505812915
     Core   RigidRotor
       SymmetryFactor          0.5
     End
     Frequencies[1/cm]          159
 16.9900                47.6757                 71.2346
 89.4994                92.3394                104.9667
111.9144               151.7908                166.4371
```

185.1307	191.8439	208.6001
229.6521	246.6694	266.0073
274.0510	285.3889	307.3781
327.3773	336.6914	353.2687
370.9060	375.9061	384.2168
421.2403	430.9152	446.0983
450.9005	459.9942	488.9335
501.4816	507.0645	517.5466
525.0806	531.2049	537.1484
551.3156	555.5167	558.1017
572.8924	582.0252	593.6477
597.6503	609.0285	615.6168
618.1270	648.0359	689.1287
695.2968	696.2805	702.3157
720.9079	732.0296	752.8853
756.6101	758.0616	768.4604
771.8919	772.4648	778.7908
793.2691	801.6325	810.0056
818.3500	821.2178	833.3818
836.2401	838.0721	847.3115
873.3713	880.8060	891.0566
895.4114	907.1611	921.0678
923.4209	935.0829	958.9669
974.5988	974.7651	975.6706
986.4876	986.5667	996.1648
1002.7496	1008.2005	1034.4448
1084.2081	1090.3314	1103.2474
1115.0880	1142.4212	1158.0080
1163.8043	1169.0971	1186.7940
1200.4434	1205.3693	1211.2334
1230.0161	1244.6144	1247.8307
1251.9393	1260.8901	1277.8345
1280.2241	1293.5519	1304.4339
1308.7578	1332.9867	1345.5294
1348.3464	1373.0664	1391.6716
1399.8887	1407.2567	1432.8317
1439.2844	1441.3792	1443.3965
1449.5762	1455.8123	1456.4126
1471.3707	1477.5277	1509.7472
1511.0851	1523.0561	1544.4560
1559.4852	1571.2561	1584.7473
1596.0580	1607.5376	1617.2219
1635.4998	1637.6080	1639.9916
1658.8644	1661.3828	1690.2376
1693.1741	3089.3122	3123.3605
3143.9882	3155.7849	3156.4151
3158.6793	3159.0775	3160.3697
3163.0081	3168.5102	3173.2179
3174.2835	3174.9299	3178.4923
3184.5490	3196.1167	3235.4116

```
     ZeroEnergy[kcal/mol]            -42.5
     ElectronicLevels[1/cm]        1
          0   2
   End
 End
 Well       i2  # i2
 Species
   RRHO
     Geometry[angstrom]    55
C   -3.4959773381   -2.4283410378   0.716971562
C   -4.1336284368   -1.1666288132   0.8811177661
C   -3.4626758006   0.0042990928   0.4225487232
C   -2.334549602   -0.1650011173   -0.349513045
C   -1.7188111821   -1.4178982197   -0.5282354272
C   -2.2281052882   -2.5657164105   0.1034171627
C   -3.8246526189   1.3411346154   0.808733498
C   -2.9256099236   2.4429778234   0.6115233319
C   -1.4616131129   2.1280444872   0.2317939926
C   -1.3890499742   0.8881803779   -0.5971783282
C   -5.3774476094   -0.9389389223   1.5632701386
C   -5.8322838907   0.3298127594   1.7935548936
C   -5.0702654253   1.510430695   1.4573979661
C   -5.4787700871   2.8191389929   1.7934154576
C   -4.6485997617   3.891057826   1.5320581579
C   -3.367377064   3.7021827951   0.9742894811
C   3.6956900072   2.8905489809   0.7926422077
C   2.9818558162   1.7561158284   0.3738202663
C   3.5792435846   0.4728435823   0.6628525546
C   4.7959778962   0.3602548146   1.3872596277
C   5.47139708   1.5344672547   1.7505462743
C   4.9233256809   2.7768248871   1.4446527653
C   2.9083852174   -0.7337942994   0.3217585226
C   3.2654974786   -2.026359503   0.8210942603
C   4.5142907697   -2.092188086   1.5241923638
C   5.2450234792   -0.9607279225   1.7632451016
C   1.6526456478   1.8265864015   -0.243139656
C   1.1192419082   0.6354766034   -0.672420453
C   1.739194512   -0.623791095   -0.3958861506
C   0.8178858727   -1.6907078763   -0.526409259
C   1.0553189969   -2.9278621638   0.1093414685
C   2.3316100765   -3.0885472132   0.6977404132
C   -0.3960844605   -1.1297436601   -0.9779454666
C   -0.2342310669   0.306832733   -1.0490484648
C   -1.3882016263   -3.7620197856   0.2693656323
C   -0.0271858487   -3.9089024304   0.2751422626
C   -0.4970025101   3.2054851189   -0.2642936301
C   0.8425262438   3.0637091175   -0.394988468
H   -3.9577085531   -3.2984700741   1.1738388945
H   -1.031841543   1.7960920623   1.200010474
H   -5.9623097556   -1.7896738964   1.8975209619
```

```
H   -6.7843470501   0.4720333582   2.2945089525
H   -6.4391689805   2.9740611558   2.2736308073
H   -4.9661279896   4.8941778627   1.7939501312
H   -2.7130048511   4.5597112589   0.8657887746
H   3.282630995   3.8768944202   0.6183744843
H   6.410097611   1.4708793831   2.2905077112
H   5.449118304   3.6781064134   1.7396661204
H   4.8704862228   -3.050771243   1.8870409016
H   6.1836176875   -1.036946752   2.3024828186
H   2.5751765541   -4.0373398254   1.1669127175
H   -1.9402606664   -4.6589356792   0.5411815273
H   0.3177259799   -4.9033514042   0.5494397299
H   -0.9188276239   4.17590966   -0.5075381503
H   1.387522019   3.9558860684   -0.6949773603
     Core   RigidRotor
       SymmetryFactor          0.5
     End
     Frequencies[1/cm]         159
  37.3451               55.0144               82.8447
 103.0818              150.4632              156.8646
 168.7840              192.2928              202.2779
 205.8853              228.3796              250.8363
 281.3527              302.7581              304.3757
 318.2924              329.5218              345.5112
 350.4132              370.7879              393.6599
 397.6388              418.9789              437.4312
 443.0208              445.8201              463.3863
 468.4731              493.5867              495.4909
 512.2871              525.2287              530.6246
 544.4932              547.8115              550.9143
 555.7010              562.9876              572.6328
 576.0312              592.1083              600.9507
 621.2607              639.5952              643.5379
 665.6755              685.2410              692.8224
 713.8194              721.3408              726.5715
 744.2436              749.3416              755.9143
 761.8260              767.5233              773.3886
 777.6156              779.7525              801.5135
 808.8689              814.4837              817.0861
 825.7922              827.3400              838.3970
 857.0070              873.7037              882.1092
 891.5963              896.4567              911.3428
 914.9703              944.8986              967.4088
 971.0729              972.4412              981.9597
 982.6510              983.8492              990.0015
 997.0815             1005.6156             1012.5471
1056.7061             1080.4863             1090.2776
1105.5201             1124.3674             1138.5608
1148.6476             1162.9006             1169.5122
1181.3617             1192.4364             1206.1574
```

```
1212.9753            1220.5406            1229.5713
1234.3746            1240.8420            1248.0194
1257.8895            1268.9902            1272.4500
1282.7297            1288.1213            1297.7390
1323.1276            1324.3998            1329.7261
1344.5067            1352.9994            1369.1274
1373.8619            1384.6666            1404.4308
1410.8009            1423.9419            1436.6174
1441.8378            1442.5012            1452.4067
1457.3358            1462.0863            1483.6237
1512.3384            1512.9879            1532.7281
1544.2528            1554.1923            1572.7303
1581.6111            1599.5885            1607.1968
1611.0488            1619.1117            1633.5085
1634.8456            1641.2476            1648.5809
1660.0267            2853.5088            3122.9905
3126.2867            3143.3894            3155.6961
3156.0721            3157.9900            3158.5283
3158.8873            3161.4823            3162.8716
3172.6351            3174.8656            3175.2038
3177.0082            3185.9402            3190.1709
      ZeroEnergy[kcal/mol]            -57.3
      ElectronicLevels[1/cm]        1
           0   2
    End
  End
  Well       i3  # i3
  Species
    RRHO
      Geometry[angstrom]    55
 C   -3.1615168453   2.5589767413   -0.5817318555
 C   -4.0748808369   1.4761449477   -0.6046313559
 C   -3.5822830168   0.1334001758   -0.5218300418
 C   -2.2223211352   -0.0473766372   -0.4037615964
 C   -1.3538117181   1.0303433032   -0.3735855987
 C   -1.7596090885   2.3661102839   -0.4772276918
 C   -4.3994845536   -1.0155143928   -0.5792249488
 C   -3.7727637827   -2.3119311634   -0.5196421461
 C   -2.3451192101   -2.3773042733   -0.4119757422
 C   -1.4926540703   -1.3240008713   -0.356741364
 C   -5.5050920482   1.5955015148   -0.726565112
 C   -6.3133339411   0.4939277206   -0.7700578533
 C   -5.7997592478   -0.855665864   -0.7033600405
 C   -6.5916079404   -2.0118458485   -0.7592426401
 C   -5.999584241   -3.273741205   -0.6975563842
 C   -4.6183272861   -3.4317810899   -0.5817587798
 C   4.487438392   -3.404806102   -0.5295065557
 C   3.6169430044   -2.3014294685   -0.4930181911
 C   4.2405930753   -1.0085787882   -0.5442798338
 C   5.6428209208   -0.8354694624   -0.662895608
```

```
C   6.4508857363   -1.9790000518   -0.7119877013
C   5.8690655455   -3.2414951934   -0.6384964302
C   3.4323888736   0.146151226   -0.4757028869
C   3.9211689901   1.4903085371   -0.5613631036
C   5.3495191887   1.6159099255   -0.6836220207
C   6.1555726247   0.514018678   -0.7267603249
C   2.1529399159   -2.4557432062   -0.3937063668
C   1.3693099251   -1.3146163412   -0.3222020482
C   2.0760716279   -0.0445409799   -0.3496070028
C   1.2067476012   1.0351245072   -0.3401230971
C   1.6038143524   2.3712353176   -0.4502430808
C   3.0057374521   2.5704380076   -0.5438168851
C   -0.0731414554   0.4605227878   -0.2990387923
C   -0.0645614759   -0.9349809431   -0.2945151852
C   -0.7668055476   3.4476077893   -0.5046095719
C   0.607334523   3.4489379264   -0.4948581675
C   0.5028997954   -4.192394452   0.2960856775
C   1.5663984984   -3.8045091862   -0.4129004744
H   -3.5548292209   3.5679252426   -0.6617807151
H   -5.9453874169   2.5854305122   -0.7880411547
H   -7.3867066689   0.6231831904   -0.8636499203
H   -7.668902085   -1.9238195324   -0.8526573756
H   -6.6278318678   -4.156114349   -0.7433782551
H   -4.1870387432   -4.4250973485   -0.541576599
H   4.0907899317   -4.4093701245   -0.4591325692
H   7.5274247258   -1.878286983   -0.801001987
H   6.5011939259   -4.1220544906   -0.6644262041
H   5.7881983678   2.6065432334   -0.7449178013
H   7.2293881066   0.6411505194   -0.8182468449
H   3.3964787915   3.5803697537   -0.6245540193
H   -1.209536272   4.439485106   -0.5612140218
H   1.0477743975   4.4421639438   -0.5460314659
H   -0.0008081335   -3.5284982252   0.9881887823
H   0.1136724036   -5.2002149206   0.2105917198
H   2.046139156   -4.5320193972   -1.061889767
     Core   RigidRotor
       SymmetryFactor          0.5
     End
     Frequencies[1/cm]         159
 16.0007               47.2786               64.5330
 88.5250               91.0359              103.6243
126.2946              153.3189              166.8292
186.5574              191.6452              207.8616
228.0618              246.4106              267.3719
273.3426              285.2717              310.9695
332.3848              337.8641              352.2931
368.1251              370.6042              380.7668
419.0194              427.8579              446.8071
449.8718              462.2352              491.1535
500.0445              510.3411              524.3817
```

```
 526.6554                529.8254                537.6163
 542.0985                554.7053                556.5263
 567.5216                573.9608                590.3372
 597.6502                604.1635                611.8651
 626.2139                653.7437                677.0233
 689.9050                692.4441                697.7332
 723.5154                732.7655                751.9789
 756.6020                762.6312                768.0394
 769.9903                774.2685                780.0294
 798.8891                799.7967                806.2157
 819.9013                823.9483                829.0631
 834.0083                838.2993                852.3704
 880.1851                890.7364                904.6338
 923.2217                923.3200                938.8766
 959.1326                974.3216                974.5174
 974.6138                986.7217                988.1538
 996.3044                998.6186               1002.2921
1025.7147               1031.6121               1050.7629
1083.6530               1097.7110               1112.1110
1132.0208               1147.5039               1159.1665
1164.6399               1170.9250               1186.2985
1203.4027               1211.2968               1214.6979
1237.2061               1244.7350               1257.7439
1260.2524               1271.1077               1285.1955
1288.8771               1301.0860               1322.8595
1328.8316               1334.3143               1345.2670
1349.5707               1373.7168               1398.6897
1404.9902               1429.5596               1434.1043
1436.5858               1440.6147               1447.7744
1453.4321               1455.1524               1460.3293
1467.9934               1470.8708               1509.3779
1511.5038               1520.1540               1545.4665
1558.9363               1570.0795               1580.0989
1593.3459               1600.7967               1609.5708
1632.5401               1636.3055               1652.8961
1660.0281               1677.0757               1684.0436
1692.0224               3123.3669               3138.0171
3144.0059               3148.3235               3156.2707
3156.5298               3159.0573               3159.6163
3162.7303               3163.5934               3174.7379
3174.8375               3177.3250               3178.7369
3190.9227               3199.7475               3230.7469
      ZeroEnergy[kcal/mol]            -41.2
      ElectronicLevels[1/cm]        1
           0   2
    End
  End
  Well       i4  # i4
  Species
    RRHO
```

Geometry[angstrom] 55

C	-3.418998063	-2.3881382557	0.772968059
C	-4.0427648028	-1.1256482985	0.9257467861
C	-3.3990163045	0.0315835313	0.3822694167
C	-2.2781569452	-0.1635412741	-0.3912972275
C	-1.6551405667	-1.4195653398	-0.5284083745
C	-2.1685344627	-2.5538524567	0.1265803281
C	-3.7195268034	1.3688049397	0.7394295578
C	-2.8354725426	2.4579184316	0.3844865201
C	-1.5947660548	2.1854913392	-0.3367161473
C	-1.3835853633	0.8981572077	-0.7409776095
C	-5.2258495241	-0.8681472735	1.6985099041
C	-5.6269105686	0.4118652889	1.9601631811
C	-4.8780134795	1.5729118506	1.534696475
C	-5.2101268378	2.8810468671	1.9135559562
C	-4.3898703342	3.9436336912	1.5442407558
C	-3.2210489875	3.7401475239	0.8122260403
C	3.8177611214	2.7383375468	0.7668500267
C	3.0639969498	1.6284842059	0.3858423296
C	3.6450784244	0.3382285632	0.6184640047
C	4.872543058	0.1895152605	1.3132844436
C	5.5805320632	1.3460631642	1.6842589029
C	5.0623438961	2.5977716942	1.3944035865
C	2.9591736459	-0.854097682	0.2252187304
C	3.3079646336	-2.1554491942	0.7006052218
C	4.5625193828	-2.2544240924	1.3946493933
C	5.3129313444	-1.143741347	1.6516831975
C	1.689106242	1.7535239628	-0.2026388992
C	1.1651446125	0.5514523063	-0.7835133151
C	1.8043986432	-0.7190273972	-0.5155074072
C	0.8755602737	-1.7711942584	-0.62552694
C	1.0912531899	-3.0121664331	-0.0031958623
C	2.3713760889	-3.2097174903	0.566014618
C	-0.3479551994	-1.158764162	-1.0158519894
C	-0.1567788479	0.2510874556	-1.1374436023
C	-1.3630375992	-3.7739615952	0.2619055143
C	-0.0095696842	-3.9634721806	0.203091118
C	-0.4685667859	3.1810702758	-0.6455459139
C	0.9016142858	2.8733409911	-0.0338050222
H	-3.8707990182	-3.2446861444	1.2645503878
H	-5.7927109957	-1.7034476323	2.0964824322
H	-6.5197714606	0.5804478844	2.5534222927
H	-6.0981251438	3.0601820826	2.5104033472
H	-4.6542068116	4.9508950071	1.8463486354
H	-2.6010642269	4.5957739781	0.5739622654
H	3.443798813	3.7358983183	0.5700188855
H	6.5288800004	1.2519417073	2.2026625669
H	5.6185110487	3.487105153	1.6689123124
H	4.9053226777	-3.2281971587	1.7289044508
H	6.2583794964	-1.2442776235	2.1745931076

```
 H   2.6068115527   -4.1682862529   1.0184053186
 H   -1.931970396   -4.6522885935   0.5582653613
 H   0.3177631257   -4.9680283987   0.4616393888
 H   -0.7457237942   4.1882197696   -0.3350679633
 H   -0.3621529997   3.2163619386   -1.7387025045
 H   1.2925410334   3.6620085982   0.5997599578
      Core   RigidRotor
        SymmetryFactor          0.5
      End
      Frequencies[1/cm]         159
  37.1726               52.9917               81.6112
 103.9952              150.1184              158.0243
 173.7564              190.7762              199.1488
 207.4597              225.5687              248.4712
 261.2300              289.2086              307.6338
 315.8672              325.8495              345.6943
 351.7307              371.2529              376.8558
 394.2502              407.2010              441.2234
 442.4470              445.5958              458.1233
 471.0622              480.4522              500.2067
 510.9653              520.5344              530.5189
 543.4479              547.5651              550.5042
 556.5331              563.4580              574.5993
 585.1012              598.7365              606.8383
 612.8621              624.1488              642.2601
 670.0075              681.2364              695.5351
 704.5220              715.4684              722.6751
 744.5292              749.2798              757.4649
 759.5196              768.2438              771.4214
 778.1252              787.2954              794.0116
 802.0611              806.3204              813.6320
 821.4731              829.8700              834.1415
 840.2109              861.3761              877.9465
 889.4530              894.6504              910.2654
 917.8137              920.4767              961.6393
 971.9389              972.4450              972.9814
 982.9125              982.9678              992.4033
 997.0689             1002.7581             1011.2850
1039.8532             1088.9124             1091.7153
1104.5461             1133.3357             1147.0912
1150.8001             1160.6389             1169.8564
1185.3149             1198.4696             1207.3829
1209.0913             1232.3827             1235.8912
1239.6153             1244.1234             1256.2095
1271.9161             1276.0343             1287.8050
1299.1150             1317.3369             1320.5152
1323.7102             1330.2191             1339.6292
1353.0170             1363.0126             1382.8163
1387.3966             1395.0689             1411.0726
1430.0090             1441.4496             1442.0409
```

```
1445.1901              1451.6239              1460.3142
1462.5198              1469.5274              1475.4397
1498.4244              1513.8880              1515.8988
1537.3641              1546.0907              1550.4309
1569.7802              1586.5001              1598.8982
1608.3528              1618.7997              1634.6133
1638.0276              1645.1689              1651.6556
1666.3175              2986.6619              3101.5708
3123.2970              3143.6835              3156.0701
3156.6685              3157.9091              3159.2491
3161.3765              3162.7759              3164.0795
3175.0679              3175.1599              3176.7847
3177.1671              3189.3678              3191.3211
      ZeroEnergy[kcal/mol]            -73.2
      ElectronicLevels[1/cm]        1
           0   2
    End
  End
  Bimolecular       R   # C36H15 + C2H2
    Fragment     C36H15
      RRHO
        Geometry[angstrom]     51
 C   -3.4970021892   -2.3904173681   -0.1419119046
 C   -4.3069790006   -1.2287355964   -0.1763836898
 C   -3.6893617597   0.0641693253   -0.1515659232
 C   -2.3167479918   0.1250085207   -0.0945062878
 C   -1.5537890419   -1.02923047   -0.0619722573
 C   -2.0789489783   -2.3241249045   -0.0829694315
 C   -4.3899055421   1.2881218346   -0.1815491911
 C   -3.6412458492   2.5117066527   -0.1512456409
 C   -2.1880722014   2.5109387788   -0.0907994177
 C   -1.4931086544   1.3260026366   -0.0610788095
 C   -5.7458568862   -1.2136497722   -0.2362529301
 C   -6.4451226265   -0.0391968849   -0.2661425832
 C   -5.8043413563   1.2564502804   -0.2403701231
 C   -6.4893155327   2.4793733721   -0.269702692
 C   -5.779232143   3.6799025507   -0.2409862342
 C   -4.3857529197   3.7029562319   -0.1830348722
 C   4.6772270488   2.8611669908   0.1937391263
 C   3.7254476507   1.8287755469   0.1550673373
 C   4.225663169   0.477169732   0.1767551487
 C   5.6081804501   0.1814052098   0.2341942984
 C   6.5094654245   1.2549857328   0.2707461432
 C   6.0400441849   2.5689253216   0.2503772462
 C   3.3004436497   -0.587439428   0.1392006407
 C   3.6640629479   -1.9732610949   0.155169025
 C   5.0802577244   -2.2309278151   0.2139879336
 C   5.9913732224   -1.2125665687   0.2510240563
 C   2.3079527428   2.0316195028   0.0961941649
 C   1.3564766575   1.066306436   0.0574698189
```

```
C   1.9609037437   -0.2748180915   0.0835167304
C   0.9940138737   -1.2647297853   0.0440757132
C   1.2717708867   -2.6359581606   0.0564350838
C   2.6517181183   -2.9639658479   0.1138538938
C   -0.2261776456   -0.5745594054   -0.0070534922
C   -0.1018979062   0.8142880964   -0.0028696288
C   -1.1876682037   -3.4911460296   -0.0451623738
C   0.1802554789   -3.6183000629   0.0117437849
H   -3.9840347148   -3.3608050955   -0.1615312362
H   -1.6851628531   3.4724167069   -0.0705502245
H   -6.2814939075   -2.1571867343   -0.2578947163
H   -7.5290677058   -0.0661147594   -0.3112173529
H   -7.5732530319   2.491535776   -0.3148027635
H   -6.3222956203   4.6180735276   -0.2642212618
H   -3.8667054832   4.6555739759   -0.1620986275
H   4.3427745124   3.891743386   0.1791477732
H   7.5762854465   1.0627063457   0.31504758
H   6.7523634611   3.3857379772   0.2792874444
H   5.4260418938   -3.2593970034   0.2289988607
H   7.0503094057   -1.4456896108   0.2950305851
H   2.9502660526   -4.0078729782   0.1268829042
H   -1.7177131877   -4.4407411042   -0.0665618755
H   0.5269351873   -4.6491388763   0.0268102476
        Core     RigidRotor
        SymmetryFactor       1
        End
     Frequencies[1/cm]         147
 11.0763                49.3239                87.9820
 91.3473               111.4327               144.7693
165.9909               183.7099               191.7806
210.0939               251.0903               256.0623
266.7246               279.1522               300.7779
331.0986               342.8412               352.3211
366.4555               373.7269               411.4426
425.6702               428.7778               448.3411
466.4424               499.3696               500.9609
512.5829               515.4615               524.4224
529.4932               530.5882               546.6962
553.7806               556.9173               561.6811
591.1746               596.2415               597.8006
604.7652               614.7496               617.0860
627.3843               676.5700               691.1031
695.7318               721.1165               740.3853
741.9484               755.3592               756.9080
767.1473               771.9103               772.2518
777.5086               801.8323               804.0250
810.3806               818.4763               821.1514
833.4114               838.3988               840.3865
873.0817               881.3244               891.5541
891.7564               901.7533               925.0161
```

```
 930.2332              971.8471              973.6763
 975.1264              975.2923              986.9233
 989.0928              996.4190             1002.5846
1015.6837             1043.1817             1081.6748
1089.6305             1113.1373             1123.8380
1144.6420             1160.3206             1164.2922
1184.7271             1196.3236             1202.4008
1205.1392             1223.1613             1243.8572
1246.9986             1256.6021             1258.7498
1280.9075             1285.8665             1299.8979
1305.7548             1322.6316             1329.3456
1343.1368             1369.6929             1389.8468
1398.4323             1406.3845             1428.6480
1433.9318             1439.2413             1442.1701
1448.8014             1455.3435             1456.3368
1469.5332             1472.0619             1508.6253
1511.3188             1519.0292             1547.3534
1557.3905             1573.3935             1582.4114
1593.6786             1600.5943             1618.2330
1633.5109             1637.6868             1654.5555
1662.6569             1685.7404             1694.7836
3123.1985             3143.8386             3156.2980
3157.0139             3159.0342             3160.0414
3160.2270             3163.7391             3166.9562
3169.5696             3174.8998             3175.2940
3177.6665             3184.2895             3191.3672
         ZeroEnergy[kcal/mol]         0.
         ElectronicLevels[1/cm]        1
             0   2
    End
Fragment        c2h2
RRHO
Geometry[angstrom]   4
C     0.000000    0.000000    0.599070
C     0.000000    0.000000   -0.599070
H     0.000000    0.000000    1.661908
H     0.000000    0.000000   -1.661908
Core  RigidRotor
SymmetryFactor 2
End
Frequencies[1/cm]  7
642.0679              642.0679              772.6955
772.6955             2069.5209             3420.9273
3523.7963
ZeroEnergy[kcal/mol]     0.0
ElectronicLevels[1/cm]        1
0  1
End
      GroundEnergy[kcal/mol]       0.0
   End
```

```
  Bimolecular      p1    # p1 + H
    Fragment        C38H16
      RRHO
      Geometry[angstrom]      54
C   -3.313803079   -2.3975575784   0.112107059
C   -3.9766651778   -1.1468417488   0.2184573796
C   -3.3246450352   0.0163080363   -0.2852637718
C   -2.1551113603   -0.1597170465   -0.9942694784
C   -1.5116693891   -1.4039872171   -1.1049436576
C   -2.0286456019   -2.5418289525   -0.4674293353
C   -3.6864704256   1.3476667373   0.052970784
C   -2.7863237835   2.4460406996   -0.21488618
C   -1.4456454441   2.1831123939   -0.7866568098
C   -1.2301729303   0.8990139153   -1.2516290259
C   -5.2154198539   -0.9116916045   0.9108859388
C   -5.6636400964   0.3592251529   1.1323066596
C   -4.907373961   1.5318248045   0.7544814782
C   -5.2920900877   2.8364859946   1.0955172435
C   -4.4638862959   3.9097506992   0.7876312245
C   -3.2264441687   3.7196481967   0.1692700346
C   3.9659796212   2.8214421217   0.1571280979
C   3.2248354501   1.6953454398   -0.2249538414
C   3.8279790533   0.4092033166   0.040705451
C   5.0587980978   0.2871935121   0.7384219955
C   5.7537013543   1.4570324556   1.0775925206
C   5.214051662   2.7011317463   0.7716165463
C   3.1487317596   -0.7921266356   -0.295818005
C   3.4961055414   -2.0800424283   0.206614021
C   4.7567137779   -2.1570245658   0.8952445204
C   5.5045078474   -1.035502046   1.1146306438
C   1.858936801   1.7704393088   -0.7924196343
C   1.3326977694   0.5789176132   -1.2560383844
C   1.9697195235   -0.6748920078   -1.0012794103
C   1.0396043997   -1.7226288968   -1.1092167339
C   1.2627156313   -2.9528134483   -0.4726418904
C   2.5456328133   -3.1292414471   0.1029694039
C   -0.1818851262   -1.1239179025   -1.5427520583
C   -0.0094326563   0.2583067798   -1.6382580437
C   -1.1960482136   -3.742938198   -0.2755515701
C   0.1608944773   -3.9123510127   -0.2776965999
C   -0.3446407948   3.1324009035   -0.7138344314
C   1.0253832898   2.9612733218   -0.7161622623
H   -3.7734860081   -3.2599433229   0.586349053
H   -5.7895506594   -1.7578212831   1.2741829319
H   -6.6003230073   0.5130947474   1.6578533641
H   -6.2295082995   3.0032023724   1.6155506966
H   -4.7705217124   4.9150962361   1.053580481
H   -2.6037107904   4.5868295575   -0.012749438
H   3.5751964503   3.8152270313   -0.0234786656
H   6.704922278   1.3878639356   1.5946730968
```

```
 H   5.7594103735   3.6000943218   1.0360503414
 H   5.1060978784   -3.118493933   1.2572616973
 H   6.4518353843   -1.1168986197   1.6373048699
 H   2.780503306   -4.0782874781   0.5763311312
 H   -1.7596650655   -4.6283889042   0.0100649638
 H   0.4904033396   -4.9093091094   0.0064974617
 H   -0.6395532468   4.1575376497   -0.5137712015
 H   1.5640283903   3.8822773855   -0.5173836615
       Core      RigidRotor
         SymmetryFactor       1
       End
       Frequencies[1/cm]       156
  40.3511                53.7137                80.8957
 105.4098               140.9020               160.3125
 178.6662               185.6855               203.4701
 209.1036               229.1116               263.2816
 284.7489               305.9413               306.1341
 321.9052               331.9070               347.2739
 357.2503               376.3761               389.3394
 416.0148               438.5986               439.6412
 446.9716               451.1707               473.7007
 479.7323               496.5492               513.6309
 515.1675               527.5081               530.6508
 543.6867               550.7031               556.4208
 558.0007               559.6157               578.4261
 588.7205               593.7768               612.6028
 623.3788               638.9305               655.2340
 675.2816               688.2300               701.8436
 719.0085               729.3136               743.4554
 747.1882               748.1068               762.6392
 762.9785               769.5355               770.8389
 778.5681               792.7990               804.7451
 806.1405               810.9565               815.5445
 825.5709               827.9678               833.1623
 835.7398               887.2835               894.4547
 900.0950               907.5905               912.0874
 914.1813               950.6659               968.5215
 970.7204               973.2631               974.7209
 982.2650               985.8941               992.7223
 997.0312              1015.7199              1029.1676
1077.0190              1092.5361              1096.3047
1136.6308              1138.3387              1156.1973
1164.3077              1177.1119              1188.6032
1203.1713              1207.7236              1212.2592
1219.3611              1239.7752              1245.2195
1255.3410              1268.4840              1281.3945
1285.0629              1292.5302              1306.9512
1309.9668              1333.7008              1334.3706
1350.5498              1354.0036              1381.5015
1388.4764              1413.0887              1416.1166
```

```
1428.9142              1438.7872              1440.3700
1444.2884              1449.7433              1454.8012
1461.2171              1462.5207              1487.2038
1501.2416              1502.8375              1518.3719
1522.2119              1538.8546              1549.1800
1566.6384              1592.2103              1594.0221
1610.0402              1622.0877              1627.6315
1627.8120              1640.3804              1648.5746
1657.4177              1670.6863              3121.4830
3142.1404              3144.2868              3154.5906
3154.7608              3157.8530              3157.8855
3162.5772              3163.3409              3165.8687
3175.4881              3175.6532              3177.9644
3178.5858              3192.4849              3193.0634
       ZeroEnergy[kcal/mol]          0.
       ElectronicLevels[1/cm]      1
         0    1
       End
     Fragment     H
       Atom
         Mass[amu]   1
         ElectronicLevels[1/cm]      1
            0    2
       End
     GroundEnergy[kcal/mol]             -30.9
   End
   Bimolecular      p2    # p2 + H
     Fragment        C38H16
       RRHO
       Geometry[angstrom]      54
 C   -3.1989353892   2.9288684699   -0.3983339129
 C   -4.0903903722   1.8274321901   -0.4014927632
 C   -3.5685202693   0.4948503433   -0.3266102996
 C   -2.2051239927   0.3368113222   -0.2395719242
 C   -1.3609503931   1.4351183687   -0.231239733
 C   -1.790535906   2.7632801855   -0.3222143068
 C   -4.3537552159   -0.6763700667   -0.3598849756
 C   -3.694153247   -1.9495377639   -0.309949206
 C   -2.2454108478   -2.0518101851   -0.2364503776
 C   -1.4688134162   -0.9185913985   -0.2031630586
 C   -5.5248704438   1.914064006   -0.4917270161
 C   -6.3061311449   0.7920932475   -0.512730301
 C   -5.760645804   -0.5451399948   -0.452634697
 C   -6.5304931547   -1.7164217494   -0.4855644985
 C   -5.9083067248   -2.9639317607   -0.4321310366
 C   -4.5212151318   -3.0849621772   -0.3481409648
 C   4.5450613857   -2.9085826866   -0.335948546
 C   3.6691191553   -1.812710827   -0.3019578896
 C   4.2690072014   -0.511514789   -0.3480482803
 C   5.6705183049   -0.3195447568   -0.433262707
```

```
C   6.4929567953   -1.4536743607   -0.4632182545
C   5.9259942126   -2.7253778786   -0.4131407507
C   3.4396853708   0.6307877982   -0.3174914634
C   3.9095398069   1.9837873704   -0.3874603744
C   5.3394160526   2.1287564037   -0.4704157663
C   6.1627784351   1.0385209187   -0.4890304438
C   2.2049041043   -1.9800260404   -0.2333356665
C   1.3908910693   -0.8565737419   -0.2021585888
C   2.0844891918   0.4217059498   -0.2359433305
C   1.1982645451   1.4860971325   -0.2287961847
C   1.5743720355   2.8285462483   -0.3165281503
C   2.9767497977   3.0488375894   -0.3863371131
C   -0.0709825616   0.8885589225   -0.1811585999
C   -0.0426909668   -0.5076819567   -0.1669076423
C   -0.816407724   3.8614393106   -0.3567090924
C   0.5586713483   3.888360179   -0.3541007533
C   1.1816396837   -4.4006012335   -0.1875098431
C   1.6511618869   -3.2899917163   -0.2085673763
H   -3.6142015532   3.929834236   -0.4678629926
H   -1.8067884136   -3.0437737014   -0.2183905148
H   -5.9906945495   2.8926730991   -0.546250665
H   -7.3841942736   0.8957600378   -0.5816922924
H   -7.6113276202   -1.6518566184   -0.5543698662
H   -6.5160741171   -3.8614296303   -0.4591408035
H   -4.0713634774   -4.0717181975   -0.3136400563
H   4.1395973223   -3.9124648112   -0.3036229255
H   7.570013248   -1.3396578127   -0.5261432057
H   6.5709925836   -3.596560092   -0.4371559794
H   5.7658547046   3.1252917105   -0.5207771128
H   7.2364500615   1.1827965299   -0.5522814511
H   3.3535175274   4.0651147879   -0.4523984802
H   -1.276212164   4.8459787837   -0.4053796181
H   0.9801788866   4.8899148044   -0.4010271316
H   0.7666711572   -5.3781549985   -0.1684990163
      Core      RigidRotor
        SymmetryFactor       1.0
      End
      Frequencies[1/cm]       156
 14.4726               45.3064               64.5947
 89.8583               92.4908               95.4924
140.2900              149.6891              166.5271
187.1701              199.2716              217.0921
244.5561              254.6422              270.2670
278.5542              297.0457              333.0972
337.4147              348.0903              369.7438
379.4179              391.7976              400.8799
422.5562              429.6769              446.3853
467.3400              470.5422              477.8659
501.5499              506.2947              517.0139
524.4072              531.0414              534.5042
```

```
 552.4112              555.4073              562.8043
 572.1485              591.8310              595.8716
 598.5645              608.7570              614.5229
 614.9813              631.6876              686.9065
 688.1212              688.3953              692.3409
 695.8746              707.7303              726.0924
 751.6208              755.9639              758.0303
 768.3753              772.3111              773.7964
 781.0068              793.5351              799.3515
 810.3884              819.1600              824.1367
 834.1266              838.3222              839.8980
 850.8826              881.3363              891.6534
 903.5191              904.9821              928.1142
 930.8345              946.6922              974.9670
 975.1182              976.0638              986.3730
 990.3242              996.3331             1003.0988
1007.3941             1035.8833             1082.3737
1086.4448             1101.3806             1114.7737
1142.0195             1160.6517             1162.7197
1171.6764             1186.3876             1199.7335
1205.2223             1214.4748             1232.5261
1244.8629             1247.2352             1259.9957
1274.7908             1279.7639             1296.1952
1304.1463             1310.2491             1333.7349
1345.7311             1351.7893             1371.5776
1391.5530             1399.7694             1406.4441
1431.8590             1437.8992             1441.9341
1443.2845             1449.6402             1454.8415
1456.2823             1471.0269             1477.7690
1508.4935             1510.9042             1523.3398
1543.7726             1556.6497             1570.8315
1584.7606             1596.3917             1609.6302
1618.3364             1635.9434             1638.2351
1659.6698             1663.2222             1691.7678
1696.7988             2189.3020             3123.6556
3144.2264             3156.0225             3156.8628
3158.9494             3159.5571             3160.2893
3163.2355             3168.6919             3174.4670
3174.8796             3175.2829             3178.8247
3184.3997             3194.9632             3477.0183
       ZeroEnergy[kcal/mol]           0.
       ElectronicLevels[1/cm]      1
         0    1
       End
     Fragment     H
       Atom
         Mass[amu]   1
         ElectronicLevels[1/cm]      1
           0    2
       End
```

```
    GroundEnergy[kcal/mol]            -10.5
  End
  Barrier      B1   R   i1    #  ts1
    RRHO
      Geometry[angstrom]     55        #
C   -3.6335237923   -2.361144424   0.8224488195
C   -4.4067579187   -1.1743715819   0.8073761411
C   -3.7475604692   0.0980277394   0.7919333095
C   -2.3725932315   0.1151820865   0.798801586
C   -1.6460720598   -1.0627204542   0.8153704312
C   -2.2127357889   -2.3401579623   0.8223990945
C   -4.4092672488   1.3434851494   0.7596977534
C   -3.6208051974   2.5420312723   0.7314467215
C   -2.1668924198   2.4951322082   0.7322004466
C   -1.5099090337   1.2886093241   0.7663932763
C   -5.8456581177   -1.1131268989   0.7989795013
C   -6.5077082151   0.0828042835   0.7733620437
C   -5.8252315931   1.3571187111   0.75054698
C   -6.4713349256   2.6009534297   0.7173232562
C   -5.7224426706   3.7777307356   0.6921019788
C   -4.3276661045   3.7559886377   0.6978482239
C   4.7008878516   2.6273152919   0.5650712711
C   3.7138803582   1.6288566195   0.6247926553
C   4.1750464589   0.2627641507   0.6248951735
C   5.5476601613   -0.077864864   0.568305237
C   6.4832319498   0.9652457318   0.5121402171
C   6.0543702514   2.2924188225   0.5105869121
C   3.2203769564   -0.7750781732   0.6782408732
C   3.5411234518   -2.1715705504   0.6756783899
C   4.9483273081   -2.4732692523   0.6203286448
C   5.8889689985   -1.4825928528   0.570251134
C   2.3000786403   1.8949714433   0.684630463
C   1.3344255577   0.9373756318   0.7229304128
C   1.8915887887   -0.4200125458   0.7272906074
C   0.8951508439   -1.3798708463   0.7716431831
C   1.12888607   -2.7591737381   0.7656568964
C   2.4982482403   -3.1304010789   0.7194479558
C   -0.3033716777   -0.6507726022   0.7980724824
C   -0.1336048735   0.732779387   0.7689164069
C   -1.358260189   -3.5352604803   0.8199560132
C   0.0058818168   -3.7060881414   0.7968038531
C   1.4044535104   4.6066638704   -0.268416475
C   1.5527892385   4.1049504555   0.8283395139
H   -4.1515666573   -3.3155676424   0.8292446158
H   -1.6300092792   3.4375022591   0.69816093
H   -6.4117780743   -2.0389203333   0.8121048824
H   -7.5929106854   0.0903805984   0.767475809
H   -7.5552837958   2.647636372   0.7103952486
H   -6.2355847467   4.7325811759   0.6662709805
H   -3.7776726485   4.6910593856   0.6754869956
```

```
 H   4.3989903328   3.6675488298   0.5555830937
 H   7.5432780477   0.7381329365   0.4683658264
 H   6.7908916149   3.0868652495   0.464445904
 H   5.2631625967   -3.5118021443   0.6171341022
 H   6.9405809075   -1.7473606609   0.5282731965
 H   2.764673019   -4.1831089187   0.7124787105
 H   -1.9187389952   -4.4673953369   0.8347428774
 H   0.3195636495   -4.7476168634   0.7967993295
 H   1.335353481   4.8794602369   -1.2943399588
 H   1.5506923081   3.9995433211   1.8887730722
       Core     RigidRotor
         SymmetryFactor    0.5
       End
      Tunneling      Eckart
        ImaginaryFrequency[1/cm]    316.6798
        WellDepth[kcal/mol]         1.4
        WellDepth[kcal/mol]         43.9
      End
       Frequencies[1/cm]     158
  12.8065               29.3586
  41.8272               51.7176               82.8517
  88.5457               91.7464              118.9785
 143.0703              164.3412              176.9076
 188.8425              205.4757              217.7337
 250.1717              257.2828              268.8979
 281.7940              307.1879              333.1253
 346.1719              351.9477              368.2228
 376.6087              413.9508              423.2575
 428.0648              449.0021              459.7455
 491.5210              500.4875              503.9163
 514.0516              523.4363              530.2426
 532.8854              536.4756              551.1192
 554.2100              558.2517              567.7755
 592.2578              595.5835              602.0992
 606.2451              614.9115              617.8930
 628.1053              659.0078              680.6101
 692.5182              695.9836              713.1230
 723.2613              744.7768              756.0673
 756.8144              766.7889              771.3267
 771.9607              773.2539              776.4103
 778.2903              800.7950              805.9539
 810.4613              818.7980              824.2478
 833.6284              838.4380              840.8230
 872.9909              880.3217              890.6216
 891.9789              906.4387              928.2991
 933.0336              971.5382              973.5851
 974.4960              974.8053              985.9676
 989.9577              995.8815             1002.4819
1016.7730             1043.7221             1082.4521
1089.6385             1112.2925             1123.5228
```

```
1145.9436             1160.2991             1163.9363
1184.5739             1195.8668             1201.9626
1205.2771             1223.4830             1243.6270
1246.9873             1254.0871             1259.3004
1280.5459             1283.9076             1299.8076
1305.7530             1323.7614             1329.2252
1343.2848             1370.2898             1390.1516
1398.4445             1406.4943             1428.4277
1433.9817             1439.7867             1441.7897
1448.4792             1454.1897             1456.1198
1469.5028             1472.9162             1508.3659
1510.9541             1517.5001             1545.9186
1557.4231             1571.1339             1581.9433
1593.8525             1599.9510             1617.9116
1633.0190             1637.4724             1655.6540
1662.4809             1687.1362             1694.5790
1938.0628             3122.4272             3143.1808
3155.5223             3155.8281             3158.3567
3158.6985             3159.5876             3162.2783
3167.9313             3170.8173             3174.0894
3174.3791             3177.7275             3183.7153
3194.6737             3393.6289             3480.6406
        ZeroEnergy[kcal/mol]         1.4
        ElectronicLevels[1/cm]        1
           0   2
   End
     Barrier    B2   i1   p2    #  ts2
     RRHO
       Geometry[angstrom]     55         #
 C   -3.2196954828   2.9412236736   -0.0460037515
 C   -4.1111001666   1.839611037   -0.0440329034
 C   -3.5876016365   0.5056768236   -0.0245948182
 C   -2.2218476491   0.3458610107   -0.0052180042
 C   -1.3778996199   1.4439809533   -0.0065511215
 C   -1.8094898434   2.774332366   -0.0293195292
 C   -4.3747202849   -0.6645646608   -0.0291746491
 C   -3.7145690435   -1.9383048605   -0.0156851091
 C   -2.2641355384   -2.0419532126   0.0014381217
 C   -1.4850215501   -0.9099111691   0.0074090818
 C   -5.5481694736   1.9279036811   -0.0636190644
 C   -6.3305920696   0.8065129969   -0.065609461
 C   -5.7842587078   -0.5316444487   -0.0497500782
 C   -6.5559447173   -1.7021682439   -0.0548824809
 C   -5.9330107444   -2.9503722657   -0.0412629891
 C   -4.5436249015   -3.0728707935   -0.0226013292
 C   4.540126899   -2.8863419612   0.0105233271
 C   3.660533016   -1.7927813676   0.0053100832
 C   4.258834715   -0.4900449797   -0.0114651134
 C   5.6619674081   -0.2938429072   -0.0257663857
 C   6.4880356873   -1.4258405217   -0.0221660429
```

```
C   5.9226898904   -2.6990632331   -0.003907162
C   3.425789068   0.6495894645   -0.01324926
C   3.8941045868   2.004796057   -0.0327002687
C   5.3259617961   2.1541965861   -0.0474701099
C   6.1522860944   1.0660541003   -0.0436992135
C   2.1983230363   -1.9611022035   0.022994161
C   1.3777893731   -0.8450050854   0.015240922
C   2.0691560914   0.4361231707   0.0030028156
C   1.1807440996   1.4979107459   -0.0022585369
C   1.5556253897   2.8433211043   -0.0248953046
C   2.9590902899   3.0677903556   -0.0380344098
C   -0.0875786276   0.8970781728   0.0072928186
C   -0.0580830449   -0.4989727132   0.0161203378
C   -0.8365137859   3.8738759124   -0.0392455198
C   0.5386556276   3.902324236   -0.0376358979
C   1.0818059405   -4.300478217   0.4319381368
C   1.6368647077   -3.2832955371   0.0531755797
H   -3.6369986177   3.9435805961   -0.0634037965
H   -1.8269374751   -3.0346184405   0.0063555389
H   -6.0151276667   2.907366129   -0.0776635512
H   -7.4106187877   0.9114885674   -0.0808074655
H   -7.6387808766   -1.6365049373   -0.0701834106
H   -6.5422050418   -3.847289304   -0.0461744815
H   -4.0938615082   -4.0602401216   -0.0142145539
H   4.1386710586   -3.8921049181   0.0252840968
H   7.566547212   -1.3090263229   -0.0332275527
H   6.5702973162   -3.5686015893   -0.0009632403
H   5.7514227028   3.1522762913   -0.0621063572
H   7.2273425376   1.2133603503   -0.0552737884
H   3.3353895709   4.0862472661   -0.0549149572
H   -1.2974560052   4.8589795237   -0.0530652936
H   0.9588678531   4.9054240566   -0.0505766001
H   0.6265837061   -5.2495429987   0.5830059926
H   2.0122191926   -3.6832912145   -1.7379794506
      Core     RigidRotor
        SymmetryFactor    0.5
      End
     Tunneling      Eckart
       ImaginaryFrequency[1/cm]    817.4746
       WellDepth[kcal/mol]         39.5
       WellDepth[kcal/mol]         7.5
     End
      Frequencies[1/cm]     158
 13.2084              45.0990
 64.4297              80.1073              91.8873
 92.3960             109.0768             149.5900
166.7021             178.0018             187.8720
200.3001             218.8733             243.3163
256.6356             271.2807             281.0599
300.3262             334.9087             337.8640
```

```
     347.4295              369.2445              379.9922
     384.9624              404.9690              429.4141
     430.0725              447.8239              460.9578
     471.9240              475.7422              482.0078
     501.9179              506.6997              517.8126
     524.4253              531.2644              534.3343
     552.3045              555.6768              562.8024
     571.9245              592.2226              596.1855
     599.0938              610.1102              614.4442
     620.0888              638.4146              686.9839
     688.6735              690.3340              695.5775
     698.0198              716.8781              726.1393
     755.5035              757.7613              758.1589
     768.3256              773.5226              775.8863
     783.2310              793.6087              803.0229
     811.0260              820.2571              824.1545
     835.1882              839.5267              839.7846
     850.5356              882.1788              892.6057
     903.2312              904.1907              928.0374
     930.7426              945.7971              974.9671
     975.4874              976.6281              986.8138
     990.9669              996.5421             1002.9079
    1006.8228             1035.3724             1079.8818
    1086.1674             1100.0608             1114.8121
    1140.9056             1160.0802             1162.6181
    1170.7693             1186.4911             1199.5890
    1205.0107             1214.3970             1231.8614
    1244.9606             1247.2261             1260.2801
    1276.0508             1279.9070             1294.7965
    1304.0140             1308.8723             1333.1847
    1345.7252             1350.7471             1371.8323
    1391.6338             1399.8967             1406.5734
    1432.0823             1437.8231             1442.1515
    1443.6584             1449.7357             1455.0952
    1456.3408             1471.1847             1478.0403
    1508.5979             1510.9202             1522.7952
    1543.8579             1556.5432             1571.4167
    1584.8829             1596.7089             1609.7628
    1618.3062             1636.3255             1638.3879
    1659.9723             1664.2106             1692.3640
    1697.7699             2084.4965             3123.8990
    3144.4355             3156.5454             3157.2811
    3159.5336             3159.7271             3160.4163
    3163.8902             3168.9401             3174.9427
    3175.2367             3175.8352             3179.3764
    3184.4764             3196.0665             3457.3459
           ZeroEnergy[kcal/mol]          -3.0
           ElectronicLevels[1/cm]        1
               0    2
       End
```

Barrier B3 i1 i2 # ts3
RRHO
Geometry[angstrom] 55 #

C	-3.4046939758	-2.7342660831	0.7417525705
C	-4.1387697073	-1.5206773319	0.7412269101
C	-3.5580313395	-0.3566933955	0.1501888003
C	-2.3278128398	-0.4854057108	-0.4548899851
C	-1.624741084	-1.6930499826	-0.461302224
C	-2.1011931923	-2.838982706	0.1885128609
C	-4.0985021205	0.9515367803	0.2536609034
C	-3.3734460852	2.0698072326	-0.28268443
C	-2.0389634024	1.8991870477	-0.8839554087
C	-1.5020855199	0.6036624538	-0.9001330424
C	-5.4163136087	-1.317647005	1.371093878
C	-5.9907868779	-0.0785022399	1.4253408922
C	-5.3585533293	1.102614269	0.8835617503
C	-5.9185384088	2.3891756173	0.947602392
C	-5.2437137096	3.4751200834	0.3998143048
C	-3.99336091	3.3220613014	-0.2071350643
C	4.0781340507	2.5134880248	0.1891233511
C	3.2558669844	1.3999011889	-0.0528758318
C	3.8694442056	0.1120188428	0.1270319041
C	5.1915413123	-0.0374953287	0.6201658453
C	5.9585080022	1.1149095843	0.8349027129
C	5.403639313	2.3691794943	0.5991429265
C	3.1088247721	-1.0612728295	-0.0968672163
C	3.5070184041	-2.3664141752	0.3370749034
C	4.8579719634	-2.4734544971	0.8189625783
C	5.6576071236	-1.3715026499	0.9228174316
C	1.8278991846	1.5132076865	-0.4153980652
C	1.1883414921	0.3659241446	-0.8502536954
C	1.8506956076	-0.9070874087	-0.6338877585
C	0.9215360687	-1.9541696846	-0.608880665
C	1.2013419921	-3.2008550435	-0.0295468922
C	2.5490807226	-3.4062693838	0.3599676772
C	-0.3228634771	-1.3563657776	-0.9061644899
C	-0.2005839489	0.030420669	-1.1140533464
C	-1.2317697647	-4.0064400678	0.3799514272
C	0.1279915956	-4.1533875055	0.283311543
C	-0.2213571048	2.9878786151	0.002482547
C	1.08587948	2.7367318834	-0.0633283925
H	-3.8323621975	-3.5941179034	1.2485544925
H	-1.723303321	2.6470264952	-1.5985922884
H	-5.9248011446	-2.1653862507	1.8187895784
H	-6.9566668764	0.0411854815	1.9051989021
H	-6.883517553	2.5330191012	1.4222802838
H	-5.6918603224	4.4611781326	0.4473663778
H	-3.4887392257	4.1917235371	-0.6154240498
H	3.693393593	3.5148902147	0.0420600987
H	6.977931741	1.0271514123	1.195354925

```
H   6.0048921064   3.2576303732   0.7564295968
H   5.2367411097   -3.4455430148   1.1173848971
H   6.6729518107   -1.4794193225   1.2901302066
H   2.8407676098   -4.3676559998   0.7721859733
H   -1.7543064782   -4.8924347977   0.7333546844
H   0.4917060997   -5.1359418955   0.5758545451
H   -0.6807917909   3.8843107499   0.4013254529
H   1.7059689711   3.5420865741   0.3376257213
      Core     RigidRotor
        SymmetryFactor    0.5
      End
      Tunneling      Eckart
        ImaginaryFrequency[1/cm]    493.0831
        WellDepth[kcal/mol]         20.4
        WellDepth[kcal/mol]         35.2
      End
      Frequencies[1/cm]     158
 44.0987                54.6762
 87.1279                89.2768                106.5713
157.6762               167.9626               177.9885
185.0567               207.6190               223.0384
231.2459               256.0818               279.3976
282.4153               301.6796               318.0014
342.5014               344.9112               366.3980
374.3636               381.6235               396.3324
416.7870               434.4961               447.5663
452.6951               467.2810               499.6596
502.0968               510.6547               519.9706
527.3557               533.5397               538.7113
548.2772               553.4170               554.2142
568.3215               578.8211               586.9770
593.1768               610.5561               617.1812
626.1977               666.5543               677.9308
682.0160               699.2379               709.8112
720.8521               723.3914               749.9575
752.9908               759.5057               762.3346
768.2505               772.4299               775.8265
785.5054               801.6652               804.5411
809.9737               816.9801               823.7080
835.2768               840.0522               846.8154
861.2579               872.7177               878.0574
888.0057               900.2251               917.4823
924.1131               952.1930               973.1913
973.7109               974.4642               984.0033
984.9296               990.8089               997.8601
999.4784              1032.6765              1037.9862
1086.1817             1093.0247              1107.9901
1112.3812             1144.7006              1162.7107
1165.3533             1176.4133              1186.1432
1196.5006             1201.0711              1209.8647
```

```
1232.5021              1241.1095              1244.0662
1256.8149              1266.2592              1266.7579
1282.4956              1292.8875              1302.6487
1318.1205              1335.8744              1341.4029
1355.3889              1359.4520              1371.4653
1391.8362              1395.8076              1399.9963
1422.6475              1440.5813              1444.1756
1446.5351              1448.9165              1455.3105
1462.8662              1468.3886              1503.3162
1509.6447              1519.5838              1533.3078
1536.7678              1553.9118              1567.2010
1579.8288              1592.1644              1603.4869
1614.4319              1621.1127              1624.0045
1637.2194              1646.0162              1666.4105
1673.4206              3060.5160              3123.2421
3143.8065              3156.1399              3156.3450
3158.2299              3158.8731              3159.2840
3163.0927              3167.1547              3174.6481
3175.3051              3177.3530              3181.1660
3184.7347              3186.4745              3191.3062
        ZeroEnergy[kcal/mol]          -22.1
        ElectronicLevels[1/cm]        1
           0   2
   End
   Barrier      B4   i2   p1    #  ts4
     RRHO
       Geometry[angstrom]     55        #
 C   -3.3091067233   -2.4014535477   0.1154309976
 C   -3.9735287376   -1.1536408983   0.2384026502
 C   -3.3233606299   0.0170263885   -0.2524235185
 C   -2.1637684938   -0.1509253725   -0.9771287112
 C   -1.5204506962   -1.3922324939   -1.1095098115
 C   -2.0262195124   -2.5359047693   -0.4748999028
 C   -3.6925580163   1.3452390075   0.0986936054
 C   -2.7937626251   2.4471130901   -0.1435357106
 C   -1.4216718291   2.17180698   -0.6632785089
 C   -1.2404987196   0.9099929956   -1.2367230967
 C   -5.2162019092   -0.9276835615   0.9270972469
 C   -5.6733811907   0.339991697   1.1505075143
 C   -4.9206451244   1.5193008637   0.7860401387
 C   -5.3149980918   2.8224777792   1.1290401404
 C   -4.486648897   3.8986429998   0.8392048804
 C   -3.23530232   3.7148318375   0.2410377972
 C   3.9246286981   2.8760442625   0.1429419775
 C   3.1973955982   1.7448953466   -0.2540352448
 C   3.8086629781   0.4622300442   0.0116110682
 C   5.0392380468   0.3480282537   0.710940913
 C   5.723141078   1.5218585962   1.0575742843
 C   5.1710412999   2.763313356   0.7607865665
 C   3.1365287265   -0.7434482951   -0.3232320335
```

```
C   3.4943535394   -2.0310566068   0.176208867
C   4.7567267256   -2.0998651825   0.8617006079
C   5.4951799129   -0.9723952879   1.0832263106
C   1.8388067219   1.8126803017   -0.830331775
C   1.3114047748   0.6177759615   -1.2751857178
C   1.9535709888   -0.6330887353   -1.0212574087
C   1.0307680366   -1.6887668024   -1.1308813665
C   1.2651980051   -2.9193377937   -0.4968212694
C   2.5517907749   -3.0868683753   0.0739759005
C   -0.1950094302   -1.0997981351   -1.5575072648
C   -0.0326856778   0.2885509913   -1.6561667328
C   -1.1852973723   -3.7313476072   -0.2931784078
C   0.1736949222   -3.8887860823   -0.3015643204
C   -0.3491661415   3.1749886883   -0.7639132056
C   1.0120879853   3.0208378252   -0.8272364983
H   -3.7638744671   -3.2702296054   0.5824542935
H   -0.8830677885   1.5828444416   1.0367336351
H   -5.788958394   -1.7783737642   1.2816924758
H   -6.6156955592   0.4853813478   1.6683816127
H   -6.2592833733   2.9830143608   1.6384017953
H   -4.7986594666   4.9014818867   1.1082510972
H   -2.6018020252   4.5806864929   0.0924438273
H   3.5187249613   3.8664499617   -0.022102934
H   6.6736959353   1.4583476717   1.5765667308
H   5.7052599644   3.665359868   1.0371689139
H   5.114401639   -3.0591456001   1.2212866158
H   6.4432093298   -1.0476543714   1.6055922818
H   2.7954358176   -4.0349044639   0.5447673578
H   -1.7400756136   -4.6221208481   -0.0069944073
H   0.5131013286   -4.8833576929   -0.0208221771
H   -0.673578915   4.2068390822   -0.676557089
H   1.5586479516   3.9579225134   -0.7863439907
      Core     RigidRotor
        SymmetryFactor    0.5
      End
      Tunneling      Eckart
        ImaginaryFrequency[1/cm]    917.3848
        WellDepth[kcal/mol]         34.0
        WellDepth[kcal/mol]         7.6
      End
      Frequencies[1/cm]     158
 38.9068               53.9609
 81.4665              105.1901              145.1482
159.2373              175.8020              188.8395
203.2934              206.8495              229.5639
259.8951              283.9617              303.7381
306.8865              321.7801              332.0808
347.0189              356.4083              375.9400
395.0688              408.8796              420.4064
432.8587              441.2236              444.6643
```

```
 462.1299              468.8354              476.8606
 493.5964              507.3027              514.6972
 518.1469              528.6139              529.8984
 544.5640              551.7144              557.1901
 558.3526              567.7947              576.9225
 585.9148              593.1324              612.3326
 624.7486              642.1869              653.9242
 674.4893              688.0246              700.3319
 719.0361              733.0556              736.3260
 746.2444              748.2033              761.5742
 764.3545              770.8716              774.0671
 778.6080              785.6753              804.9048
 807.9173              812.1249              817.2542
 826.6311              827.8313              835.7284
 849.2511              886.1115              893.6903
 898.3487              907.5711              911.9758
 913.6432              948.1543              969.8084
 971.1551              973.2053              975.6826
 982.5739              985.1692              992.5219
 997.1327             1014.2077             1027.5089
1076.1743             1091.8011             1095.0425
1131.0203             1137.6533             1155.3154
1165.0414             1174.1916             1187.5447
1200.8265             1206.3695             1211.3958
1218.8789             1238.7507             1244.6176
1254.4184             1267.1508             1278.1072
1283.0799             1291.5086             1295.6308
1310.2984             1332.0427             1333.5552
1348.8634             1355.6410             1379.5599
1386.7622             1405.6379             1411.2431
1416.4822             1437.2010             1437.9789
1443.1525             1449.1689             1451.0565
1458.9119             1459.8596             1486.1897
1490.2627             1511.3364             1520.4600
1525.9105             1535.9082             1550.0349
1560.5962             1586.5176             1591.9808
1602.1319             1613.7070             1621.3250
1629.7155             1638.6523             1646.8312
1657.8901             1670.2561             3122.2684
3142.7937             3144.4967             3155.3550
3155.8158             3158.1671             3158.2211
3162.9528             3163.6354             3166.1755
3175.7045             3175.8634             3178.2347
3178.7611             3192.4960             3193.4425
        ZeroEnergy[kcal/mol]          -23.3
        ElectronicLevels[1/cm]        1
           0   2
   End
   Barrier      B5   i1   i3    #  ts5
     RRHO
```

Geometry[angstrom]	55	#	
C	-0.3604460581	2.5486855216	-0.1563645529
C	0.4837593959	1.4176516633	-0.0267623694
C	-0.0452060908	0.1162394738	-0.3036155487
C	-1.3314843208	0.0412678075	-0.7856478462
C	-2.1478966982	1.1628406146	-0.9059790922
C	-1.7258126592	2.4460146927	-0.5371147627
C	0.6034687993	-1.098315001	0.0288174142
C	-0.1148783902	-2.3487551808	-0.078713499
C	-1.4974199342	-2.3326849194	-0.4982387877
C	-2.1114851826	-1.1756441968	-0.8570546115
C	1.8377322158	1.4360600537	0.4630408993
C	2.5171346972	0.2759858846	0.7082784068
C	1.9251847106	-1.0323623377	0.5346672132
C	2.566023907	-2.2320728644	0.8760395359
C	1.8966428144	-3.4483307734	0.7458201503
C	0.5795942502	-3.5116163782	0.2917045067
C	-7.7578487564	-3.2899026116	0.2373071674
C	-6.9587941626	-2.1809325644	-0.0940718672
C	-7.5971514172	-0.8941468733	0.030700108
C	-8.9323100089	-0.7425528082	0.4873519582
C	-9.6729844706	-1.8940370299	0.7811110111
C	-9.0824559224	-3.1466674032	0.649507286
C	-6.8663233038	0.2833854813	-0.2536542502
C	-7.3328557882	1.6155250732	-0.0052045399
C	-8.6968843932	1.7154453081	0.4407447039
C	-9.4492758618	0.5969257502	0.6562688588
C	-5.5366809782	-2.2993527026	-0.4852200092
C	-4.8630146757	-1.1299102808	-0.794272892
C	-5.5749030166	0.1264457747	-0.6945543866
C	-4.7193383161	1.2149790503	-0.8507683803
C	-5.0749816925	2.5229322999	-0.5084769151
C	-6.4337457554	2.7005591912	-0.1331095331
C	-3.4508359366	0.6487321408	-1.1075407294
C	-3.4856939662	-0.7459692695	-1.0771570892
C	-2.6881881858	3.5531892204	-0.4412135063
C	-4.0607180606	3.5844797913	-0.4341663325
C	-4.8869086244	-3.6197770375	-0.5184564138
C	-3.6032131526	-3.9945629344	-0.5030604921
H	0.038041279	3.52045977	0.11992362
H	2.3167079177	2.3903699408	0.6565307737
H	3.5341935674	0.3228892755	1.0837992989
H	3.5819774681	-2.2109652073	1.2561521898
H	2.4053321661	-4.3659897591	1.0190440584
H	0.0781000482	-4.4708256492	0.2290664101
H	-7.350192025	-4.2908180958	0.1917699297
H	-10.6987551058	-1.8060042641	1.1230393023
H	-9.6564063376	-4.035410173	0.8867636156
H	-9.1248322316	2.6965709466	0.6187778696
H	-10.474693134	0.6995194215	0.9960787083

```
 H   -6.7868780329   3.694857289   0.1237179702
 H   -2.2216327879   4.5232323083   -0.2846408047
 H   -4.4803738991   4.575418173   -0.2757985819
 H   -5.5788018568   -4.4605030849   -0.5712966345
 H   -3.2999818155   -5.0366033073   -0.5484505447
 H   -2.4523282311   -3.2671042099   -0.4462619934
       Core     RigidRotor
         SymmetryFactor    0.5
       End
       Tunneling      Eckart
         ImaginaryFrequency[1/cm]    1776.9055
         WellDepth[kcal/mol]         19.9
         WellDepth[kcal/mol]         19.6
       End
       Frequencies[1/cm]     158
  27.0994               53.4598
  80.5891               89.2284               105.4271
 146.0749              158.5428               169.6831
 188.3937              201.1465               219.4175
 239.2139              261.3832               272.9032
 283.3930              295.3275               322.8503
 334.4475              340.8749               363.7296
 372.9121              374.8878               382.3738
 386.7784              423.4480               436.0367
 449.3818              452.2020               469.2853
 502.9339              503.0902               515.6172
 520.8129              528.6620               532.5815
 544.6139              547.9968               554.5187
 563.7292              572.6298               584.9589
 592.1385              606.0684               615.1617
 631.0248              634.1667               669.1621
 682.7184              690.3112               694.2961
 709.3942              726.8729               733.3682
 752.7406              755.2961               761.4799
 765.7379              769.3976               772.1078
 777.3879              802.4772               805.0517
 807.4608              813.8677               825.8537
 832.9569              835.6299               852.0985
 863.5550              882.1188               891.2961
 899.3410              907.9703               918.3333
 920.7369              956.0241               973.6848
 974.1109              974.8154               984.4944
 986.5917              998.1111               999.1658
1007.4948             1039.6273              1045.9346
1073.9686             1087.3029              1099.8294
1124.0495             1135.6040              1151.6659
1161.9952             1171.9712              1181.3477
1186.7430             1204.1149              1210.1149
1215.0372             1241.4787              1245.3212
1256.8986             1267.3823              1272.1548
```

```
1280.7037              1297.6263              1315.3341
1322.7798              1334.0174              1335.0495
1351.4319              1362.3144              1369.4455
1398.3331              1400.3376              1425.1441
1433.9558              1437.5171              1443.6416
1445.4766              1453.0793              1454.7919
1463.9602              1467.9429              1502.1904
1512.1285              1512.9328              1529.9430
1545.3691              1551.0391              1557.4940
1587.1304              1593.0703              1602.4689
1619.3969              1625.8137              1630.6302
1639.0392              1649.8833              1654.1696
1675.7890              1685.3833              3093.2277
3122.8833              3143.4593              3156.0749
3156.8740              3157.8709              3157.9478
3158.7887              3162.5761              3163.8399
3173.1808              3175.4335              3175.6880
3179.7423              3187.1334              3201.6706
        ZeroEnergy[kcal/mol]          -22.6
        ElectronicLevels[1/cm]        1
           0   2
   End
   Barrier      B6   i3   i4    #  ts6
     RRHO
       Geometry[angstrom]     55        #
 C   -3.4932718794   -2.3853958976   0.4472158975
 C   -4.1742985511   -1.145750492   0.5251853225
 C   -3.529395965   0.034331277   0.0353681409
 C   -2.3119873469   -0.1155554687   -0.5901381834
 C   -1.6541091369   -1.3459676864   -0.6677692996
 C   -2.1843558038   -2.5079409597   -0.0874604388
 C   -3.9825782855   1.3525708154   0.2880128299
 C   -3.1426304076   2.4808838856   -0.0551126983
 C   -1.8521612538   2.2350298154   -0.6476059711
 C   -1.416842561   0.9795331361   -0.9086394182
 C   -5.4486947697   -0.9321943537   1.1587980227
 C   -5.9482304631   0.3274825197   1.335770929
 C   -5.2301149029   1.5184565862   0.9400622244
 C   -5.6751000487   2.8222358987   1.2024068596
 C   -4.8880362317   3.9186471651   0.8524156022
 C   -3.6428528104   3.7582097695   0.2457193355
 C   4.168532496   2.6542339322   0.2036998111
 C   3.3300749214   1.5711329084   -0.0983407406
 C   3.8725315429   0.2577195733   0.1196942615
 C   5.1536499001   0.058880788   0.6953893793
 C   5.9415450319   1.1840371318   0.9770721599
 C   5.4513886091   2.4600008199   0.7186770813
 C   3.087827477   -0.8905864106   -0.1655061874
 C   3.4218858139   -2.2134323715   0.2687297135
 C   4.7386707384   -2.3691241849   0.8282902052
```

```
C   5.5605685472   -1.2936572642   1.0035710631
C   1.947592184   1.7522396203   -0.5811736224
C   1.2537276702   0.6080706693   -0.9651328192
C   1.8571449546   -0.6912298329   -0.7497461729
C   0.8901576439   -1.7019598148   -0.7808560834
C   1.1095087454   -2.968416652   -0.2245401045
C   2.4331874348   -3.2225724641   0.2217640794
C   -0.331430476   -1.0406457436   -1.0757298444
C   -0.142931795   0.3428239139   -1.2063789886
C   -1.3580343531   -3.7112100027   0.0745836821
C   -0.0018198029   -3.8995425114   0.0155790883
C   0.1464814322   3.3956945023   -1.2136280093
C   1.2539646358   3.0197616121   -0.4851069262
H   -3.9637947571   -3.2582460143   0.8900201846
H   -6.0103907801   -1.7885616058   1.5175086314
H   -6.9082288521   0.4568132592   1.8250563928
H   -6.6307417858   2.9760614207   1.6925371452
H   -5.2455318144   4.9192415783   1.0681457517
H   -3.0420045664   4.6279179178   0.006456393
H   3.8242408898   3.6657175348   0.0251856476
H   6.9303494989   1.056081204   1.4047422131
H   6.0709905862   3.3238527792   0.9316884078
H   5.0704457954   -3.357190305   1.1301408457
H   6.5475472449   -1.4380799606   1.4307488812
H   2.6749635779   -4.2005176315   0.6272617859
H   -1.919951643   -4.5926859856   0.3750342985
H   0.3210862513   -4.9045119424   0.2780442319
H   -0.2503088671   4.3947212677   -1.0883230984
H   -0.0928886386   2.899275242   -2.1435182771
H   1.613087926   3.7117860118   0.2712163846
      Core     RigidRotor
        SymmetryFactor    0.5
      End
      Tunneling      Eckart
        ImaginaryFrequency[1/cm]    340.3757
        WellDepth[kcal/mol]         8.9
        WellDepth[kcal/mol]         40.9
      End
      Frequencies[1/cm]     158
 39.5868              54.3623
 86.5724              95.0496              124.3571
160.2225             166.8533              179.0638
198.6928             203.0634              224.2965
251.5728             275.4417              277.5813
294.5205             304.7119              327.1683
340.3706             346.0688              372.3341
373.9694             382.1202              388.3255
418.4763             434.5292              443.3431
451.4068             459.1809              497.1002
501.0620             505.1115              522.1221
```

```
 529.9387              534.6857              537.8340
 548.1052              552.1234              557.9236
 562.9785              578.9876              589.4978
 599.3332              606.1197              617.6186
 634.6537              659.1868              673.1193
 689.8627              695.9548              705.8316
 717.2761              739.7565              750.5669
 752.9840              760.3888              766.5321
 771.6746              773.0838              777.8503
 802.2814              805.3048              805.5902
 811.9337              820.6314              827.7219
 834.4561              838.5862              845.6689
 881.2531              889.8089              899.6258
 907.3288              920.3452              922.6187
 945.4828              969.5247              973.8217
 974.4842              975.4331              985.9964
 986.3909              996.6109              998.8098
1011.0207             1040.7437             1057.8384
1085.6448             1094.5074             1117.1753
1137.8633             1156.0538             1160.3278
1169.0173             1182.0155             1186.1871
1201.8764             1209.0054             1214.0312
1240.1264             1242.3214             1248.3135
1260.7683             1269.0487             1274.7957
1284.7548             1296.1824             1316.8799
1320.6607             1335.3221             1346.8476
1349.6168             1367.8286             1393.2244
1395.8151             1400.4082             1420.2866
1426.7181             1432.8300             1441.5936
1447.3998             1451.8998             1458.3815
1462.5828             1468.0293             1498.6673
1509.7333             1516.7956             1528.8374
1538.0851             1542.4042             1557.9128
1582.9654             1589.5683             1604.1377
1609.2725             1619.3986             1623.8608
1637.4241             1645.4510             1663.8509
1670.9459             3123.4134             3143.9000
3145.7280             3155.8840             3156.4256
3156.7202             3157.7023             3158.3029
3162.6889             3163.0413             3174.4843
3175.4493             3175.8801             3177.5211
3189.0942             3191.5604             3246.5669
        ZeroEnergy[kcal/mol]          -32.3
        ElectronicLevels[1/cm]        1
           0   2
   End
   Barrier      B7   i4   p1    #  ts7
     RRHO
       Geometry[angstrom]     55        #
C                  -2.939457    2.961402   -0.314757
```

C	-3.755791	1.801583	-0.377196
C	-3.256728	0.584929	0.172883
C	-2.073759	0.637399	0.879398
C	-1.277218	1.793352	0.945957
C	-1.646265	2.963410	0.264571
C	-3.786131	-0.701131	-0.114162
C	-3.034603	-1.894109	0.202872
C	-1.672973	-1.781264	0.769284
C	-1.292769	-0.519926	1.183006
C	-5.014689	1.699478	-1.065445
C	-5.621623	0.488244	-1.239021
C	-5.021647	-0.755564	-0.811800
C	-5.571808	-2.013032	-1.099267
C	-4.889391	-3.170277	-0.741348
C	-3.637126	-3.115834	-0.126112
C	3.627194	-3.132934	-0.130922
C	3.030317	-1.909637	0.199542
C	3.789656	-0.720373	-0.108499
C	5.028508	-0.779018	-0.799917
C	5.573495	-2.038383	-1.089274
C	4.882756	-3.192750	-0.739586
C	3.266033	0.568403	0.181297
C	3.774894	1.783622	-0.363717
C	5.036727	1.676479	-1.046033
C	5.637912	0.462696	-1.219949
C	1.661371	-1.791842	0.757425
C	1.289381	-0.526094	1.181489
C	2.082236	0.626878	0.885711
C	1.293319	1.787545	0.951775
C	1.669976	2.955669	0.272933
C	2.965712	2.947781	-0.301892
C	0.005369	1.363569	1.399576
C	0.001528	-0.025472	1.548438
C	-0.668869	4.041747	0.033318
C	0.698726	4.038666	0.037055
C	-0.701333	-2.870053	0.743473
C	0.689486	-2.863425	0.703549
H	-3.286431	3.856735	-0.822547
H	-5.476515	2.596993	-1.463768
H	-6.570791	0.434889	-1.761952
H	-6.523741	-2.079419	-1.615367
H	-5.324869	-4.137944	-0.963552
H	-3.134434	-4.047704	0.101569
H	3.116850	-4.062879	0.087545
H	6.527792	-2.107909	-1.600546
H	5.313406	-4.162208	-0.963404
H	5.505413	2.572733	-1.439139
H	6.589636	0.405504	-1.737784
H	3.319738	3.842209	-0.806374
H	-1.115928	4.979904	-0.287921

```
H                   1.151798    4.974730   -0.281837
H                  -1.117085   -3.844107    0.511668
H                  -0.932118   -3.664894    2.789738
H                   1.102778   -3.847036    0.505729
       Core     RigidRotor
         SymmetryFactor    0.5
       End
       Tunneling      Eckart
         ImaginaryFrequency[1/cm]    393.2213
         WellDepth[kcal/mol]         48.0
         WellDepth[kcal/mol]         5.7
       End
       Frequencies[1/cm]     158
  40.6835               54.2117
  81.3852              105.1217              129.6345
 159.9140              173.0243              183.3132
 195.2255              204.2243              208.9961
 220.2802              236.8231              263.7102
 286.3524              306.7862              308.0764
 325.2345              334.3992              348.9928
 359.7455              376.3711              389.9256
 417.2615              438.6612              441.3680
 447.5503              452.7104              474.1061
 482.2292              495.2130              512.7747
 516.0525              527.6538              531.3152
 543.8873              550.5524              556.5334
 557.9680              564.8082              578.5214
 589.5833              594.9890              612.6817
 624.3732              636.1495              655.2683
 674.9524              690.5940              702.0058
 719.4417              728.6159              744.9414
 747.3164              749.2428              761.6583
 763.5873              768.5442              771.2621
 778.6954              796.5243              805.3270
 806.6142              811.5915              816.3363
 826.1037              828.1002              833.5649
 836.1443              887.7723              894.7711
 899.7967              906.7026              913.4057
 915.1284              949.9911              969.7280
 971.2931              973.3252              975.3787
 983.0879              986.0916              992.6513
 997.4264             1015.9845             1029.2501
1074.7248             1092.6198             1096.1815
1136.2724             1138.3315             1156.3023
1164.1683             1177.1118             1188.6031
1202.3236             1207.5035             1212.4497
1218.6542             1239.8034             1244.9566
1254.9940             1266.3820             1280.9879
1283.1446             1292.1041             1302.4183
1310.2289             1333.7804             1334.3423
```

```
1348.9858              1353.6987              1381.3866
1387.7914              1412.5344              1415.6426
1425.8379              1438.7010              1440.2541
1444.2277              1448.9501              1453.6173
1461.3097              1462.5599              1482.9759
1489.1553              1501.1147              1516.4154
1522.2707              1537.8164              1548.0385
1565.4768              1589.9632              1593.9378
1608.6541              1621.1190              1622.0500
1625.6435              1639.9299              1647.4498
1657.3469              1670.5011              3122.1172
3142.6810              3151.9322              3155.0875
3155.3252              3158.2499              3158.3334
3163.5775              3163.9132              3172.1739
3175.8710              3176.0922              3178.4174
3179.8566              3192.7288              3193.7906
        ZeroEnergy[kcal/mol]          -25.2
        ElectronicLevels[1/cm]        1
           0   2
   End
   Barrier      B8   i2   i4    #  ts8
     RRHO
       Geometry[angstrom]     55        #
 C   -3.3272039694   -2.3816977398   0.1553870111
 C   -4.006274274   -1.1328007048   0.2482440067
 C   -3.3607639816   0.0375338417   -0.2381790828
 C   -2.173276784   -0.1175393904   -0.924266414
 C   -1.522084289   -1.3597846004   -1.0352039058
 C   -2.031839831   -2.5059342807   -0.4072789095
 C   -3.7717820007   1.3682581476   0.0643660751
 C   -2.9022729046   2.490531881   -0.1887879857
 C   -1.4813066922   2.2216518277   -0.6115177717
 C   -1.2641101514   0.9439334809   -1.1957998242
 C   -5.2747637063   -0.9129193843   0.8900596487
 C   -5.7695306938   0.3500547806   1.0621063418
 C   -5.0336892557   1.5357969429   0.6870544802
 C   -5.4817700642   2.8431979175   0.9510720714
 C   -4.683023206   3.9311865734   0.6353025786
 C   -3.3995911303   3.7591454202   0.0951365222
 C   4.0444917035   2.8662564136   0.0801148501
 C   3.2728965968   1.7469209948   -0.2609066318
 C   3.8779057419   0.4567498791   -0.0271568713
 C   5.1409457842   0.3235944233   0.6068796808
 C   5.8633841661   1.4862123931   0.9107198137
 C   5.3213224088   2.7356407411   0.6297007392
 C   3.1752882111   -0.7388391375   -0.336511409
 C   3.5381110831   -2.0349281163   0.1413405254
 C   4.8299179788   -2.1209084232   0.7692621741
 C   5.593926825   -1.0045415859   0.9570321983
 C   1.8758394801   1.8406433562   -0.751416338
```

```
C   1.3285957617   0.6527723019   -1.2185581576
C   1.9708372913   -0.6068214822   -0.9903180677
C   1.0416561499   -1.6591287155   -1.0743644752
C   1.2793029118   -2.8939750595   -0.459049512
C   2.585382272   -3.0816533495   0.0682838294
C   -0.1945635558   -1.0573895359   -1.4538910719
C   -0.0346087951   0.3284706846   -1.5442254216
C   -1.1797292174   -3.6929147401   -0.2148977757
C   0.1805396841   -3.8512725406   -0.23688741
C   -0.3446291471   3.174736949   -0.4469387419
C   1.0620239407   3.024432425   -0.5713620279
H   -3.7873806759   -3.2502678966   0.616870051
H   -0.8310627186   2.3159545286   0.5154852535
H   -5.8440510454   -1.7658715293   1.2452722819
H   -6.7350471203   0.4861667665   1.538070992
H   -6.4527572243   2.9954099771   1.4104493482
H   -5.0422762799   4.9355276413   0.8286912898
H   -2.7952083957   4.6379224861   -0.0992166145
H   3.6555519215   3.8629144679   -0.0889189423
H   6.8390865408   1.4078723573   1.3786245133
H   5.8889286382   3.6296291573   0.8623812901
H   5.1913319382   -3.0858210003   1.1097270937
H   6.5649751282   -1.0946283435   1.4328670393
H   2.8381744397   -4.037183871   0.5184226178
H   -1.7283038432   -4.5825732293   0.086748612
H   0.5187030398   -4.8445291155   0.0504519763
H   -0.6536599792   4.1626098102   -0.121880662
H   1.5884272947   3.9222332048   -0.2718108816
      Core     RigidRotor
        SymmetryFactor    0.5
      End
      Tunneling      Eckart
        ImaginaryFrequency[1/cm]    1639.4327
        WellDepth[kcal/mol]         38.8
        WellDepth[kcal/mol]         54.7
      End
      Frequencies[1/cm]     158
 45.9733              55.9214
 87.1750              104.8667              134.0341
161.4110              170.6894              182.5621
203.4743              209.3547              231.2515
255.5641              280.1205              300.0582
308.2900              309.4346              336.8817
348.3739              353.2304              373.8647
387.0852              409.3301              424.5457
438.5066              445.0506              454.7468
467.1462              475.4238              480.8918
501.1533              514.4399              528.6908
529.2648              542.4563              550.9905
555.8325              556.9462              572.0353
```

```
 576.2546               585.4977               591.7778
 607.3765               619.7850               626.5364
 653.0268               671.5488               680.1871
 697.2139               711.6604               723.7938
 728.0493               741.8082               744.9366
 749.2039               757.3125               758.1005
 766.9615               768.9089               776.0819
 800.0280               802.3967               805.6364
 812.8672               817.8093               825.9027
 832.9493               839.7418               869.9132
 883.2891               893.9350               898.2256
 904.7823               915.6691               917.7320
 943.2629               969.6244               970.3866
 970.8121               980.3961               980.8474
 991.8375               996.9833              1012.3207
1015.6946              1061.6121              1089.0022
1093.5702              1109.3311              1125.9062
1135.4529              1154.3754              1161.9763
1172.3325              1182.6709              1191.8672
1202.5782              1208.5385              1213.2496
1232.3479              1241.4362              1245.6500
1257.9888              1270.8189              1273.7645
1279.9879              1294.0801              1308.4273
1330.2623              1331.4968              1341.3592
1351.2229              1373.7107              1382.0274
1389.8522              1408.3387              1414.7118
1428.8451              1433.6468              1441.2941
1442.6954              1447.4392              1452.2387
1457.2611              1461.7339              1476.4023
1491.5393              1509.3039              1517.3429
1519.5321              1532.4967              1553.7839
1564.6136              1578.7325              1595.7040
1604.8070              1609.8836              1620.0136
1634.8884              1641.6165              1652.2227
1665.6648              1834.7983              3120.4284
3141.3036              3155.6173              3156.1571
3157.4273              3158.3953              3161.4138
3162.4709              3164.1424              3173.1071
3174.6333              3175.1036              3176.6024
3184.0147              3187.8181              3193.9864
        ZeroEnergy[kcal/mol]           -18.5
        ElectronicLevels[1/cm]         1
           0   2
   End
End
```
